# A cyclopeptide and three oligomycin-class polyketides produced by an underexplored actinomycete of the genus *Pseudosporangium*

**DOI:** 10.3762/bjoc.16.97

**Published:** 2020-05-25

**Authors:** Shun Saito, Kota Atsumi, Tao Zhou, Keisuke Fukaya, Daisuke Urabe, Naoya Oku, Md Rokon Ul Karim, Hisayuki Komaki, Yasuhiro Igarashi

**Affiliations:** 1 Biotechnology Research Center and Department of Biotechnology, Toyama Prefectural University, 5180 Kurokawa, Imizu, Toyama 939-0398, Japan; 2 Biological Resource Center, National Institute of Technology and Evaluation (NBRC), Kisarazu, Chiba 292-0818, Japan

**Keywords:** DFT-based calculation, oligomycin, peptide, polyketides, *Pseudosporangium*, rare actinomycetes

## Abstract

Aside from the well-studied conventional actinomycetes such as *Streptomyces*, the less investigated genera of actinomycetes also represent a promising source of natural products. Genome mining indicated that members of the underexplored genus *Pseudosporangium*, from which no secondary metabolites have been reported to date, may harbor the biosynthetic machinery for the formation of novel natural products. The strain RD062863, that is available at a public culture collection, was obtained and subjected to metabolite analysis, which resulted in the discovery of a novel cyclopeptide, pseudosporamide (**1**), along with three new oligomycin-class polyketides, pseudosporamicins A–C (**2**–**4**). The unusual structure of compound **1**, featured by a biaryl-bond bridging across a tripeptide scaffold, *N*-acetyl-ʟ-Tyr-ʟ-Pro-ʟ-Trp, was determined by a combination of spectroscopic analyses, chemical derivatization, ECD calculation, and DFT-based theoretical chemical shift calculation, revealing the presence of an (*S*_a_)-axial chirality around the biaryl bond. Compounds **2**–**4** lacked hydroxylation on the side chain of the spiroacetal rings, which showed clear contrast to other oligomycin congeners and related polyketides with ring-truncation or expansion. The new macrolides **2**–**4** displayed potent antimicrobial activity against the Gram-positive bacterium *Kocuria rhizohpila* and the plant pathogenic fungus *Glomerella cingulata*. All compounds showed moderate cytotoxicity against P388 murine leukemia cells with IC_50_ values in the micromolar to submicromolar ranges. These results exemplified the validity of phylogeny-focused strain selection combined with biosynthetic gene-directed genome mining for the efficient discovery of new natural products.

## Introduction

Microbial secondary metabolites have been used as therapeutic drugs [1], veterinary medicines [2], agrochemicals [3], food preservatives/colorings [4–5], medium supplements for selective microbial/cell culture [6–8], or biochemical reagents for pharmacological/chemical biology studies [9] and continue to be indispensable to support and improve human welfare and social life. In recent years, in accordance with the advancement of genome and in silico analytical technologies, the searching process for new microbial secondary metabolites became faster and more efficient [10]. However, there existed a substantial number of unstudied bacterial genera for which secondary metabolic ability is still unknown at the genus level. The 16S rRNA gene sequences are widely used as an indicator of the taxonomic position of prokaryotes. It was believed that a high similarity of the 16S rRNA gene sequence implied the closeness or even the identity in other sets of genes including secondary metabolite biosynthetic genes. However, our recent analysis of *Streptomyces* species demonstrated that the distribution of secondary metabolite biosynthetic genes was not the same even in phylogenetically close species [11]. This finding became our starting point to explore the secondary metabolism in actinomycete genera from which no secondary metabolites were described.

**Figure 1:**
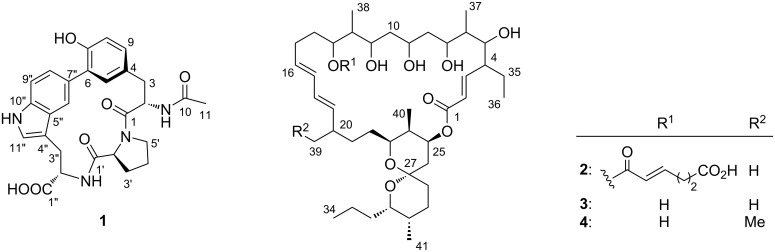
Structures of pseudosporamide (**1**) and pseudosporamicins A–C (**2**–**4**).

“Rare actinomycetes” refers to non-*Streptomyces* actinomycetes [12] and the representative genera, such as *Micromonospora*, *Actinomadura*, *Nocardia*, *Actinoplanes*, and *Saccharothrix*, which are no longer rare in terms of difficulties in isolation, already provided thousands of new metabolites [13–14]. Meanwhile, the number of genera within *Micromonosporaceae*, one of the major families in the phylum *Actinobacteria*, was still increasing, adding more than ten new genera over the past decade to index 35 names in the approved list [14]. While *Micromonospora*, the most intensively studied genus of *Micromonosporaceae*, gave over 400 natural products [15], no secondary metabolites are reported from at least 24 genera. One such genus, *Pseudosporangium*, originally discovered from a sandy soil in Bangladesh in 2008 by Takahashi and co-workers [16], appeared promising, as genome mining in the draft sequence of *P. ferrungineum* DSM 45348, using the AntiSMASH database [17], and identified in total 14 biosynthetic gene clusters for polyketides and non-ribosomal peptides. Intrigued by this, a strain available from the NBRC culture collections [18] was cultured and examined by HPLC–DAD analysis, which gave several peaks in the culture extract of the strain *Pseudosporangium* sp. RD062863. Consequently, HPLC–DAD analysis-guided purification led to the discovery of a novel cyclic peptide, pseudosporamide (**1**), and three new 26-membered macrolides, pseudosporamicins A–C (**2**–**4**, Figure 1).

## Results and Discussion

The producing strain was cultured in A3M liquid medium under shaking conditions at 30 °C for 6 days, and the whole culture broth was extracted with 1-butanol. The extract (14.6 g from 6 L) was fractionated by silica gel column chromatography, followed by ODS column chromatography. Final purification was achieved by reversed-phase HPLC separation to yield pseudosporamide (**1**, 16 mg) and pseudosporamicins A–C (**2**–**4**, 11 mg, 9.8 mg, and 16 mg, respectively).

Pseudosporamide (**1**) was obtained as a white amorphous solid. HRESITOFMS analysis gave a deprotonated molecular ion [M − H]^−^ at *m/z* 503.1936, corresponding to the molecular formula C_27_H_27_N_4_O_6_. ^13^C NMR and HSQC spectroscopic data revealed the presence of 27 resonances assignable to four carbonyl carbons, seven sp^2^ non-protonated carbons, seven sp^2^ methines, three sp^3^ methines, five sp^3^ methylenes, and one methyl carbon (Table 1).

**Table 1:** NMR spectroscopic data for pseudosporamide (**1**) in DMSO-*d*_6_.

	**1**		
	
No.	δ_C_^a^, type	δ_H_, mult (*J* in Hz)^b^	HMBC^c^

1	168.1, C		
2	50.9, CH	4.82, t (7.4)	1, 3, 4, 10
3a	35.1, CH_2_	2.80, d (13.2)	1, 4, 5, 9
3b		3.04, dd (7.2, 13.2)	1, 2, 4, 5, 9
4	126.1, C		
5	133.3, CH	7.27, d (1.9)	3, 7, 9
6	128.0, C		
7	152.8, C		
8	115.2, CH	6.77, d (8.2)	4, 6, 7
9	129.2,^d^ CH	6.68, dd (1.9, 8.2)	3, 5, 7
10	168.8, C		
11	22.5, CH_3_	1.86, s	10
1'	171.1, C		
2'	58.8, CH	4.62, dd (3.1, 8.4)	1', 3', 4', 5'
3'a	29.2, CH_2_	1.81, m	
3'b		2.06, m	1', 4'
4'a	24.3, CH_2_	1.92, m	2'
4'b		2.02, m	3'
5'a	46.7, CH_2_	3.63, m	3', 4'
5'b		3.69, m	3', 4'
1''	174.5, C		
2''	53.0, CH	4.53, brt (7.7)	1', 1'', 3'', 4''
3''a	30.6, CH_2_	2.80, d (14.8)	1'', 2'', 4'', 5'', 11''
3''b		3.33, dd (6.3, 14.8)	1'', 2'', 4'', 5'', 11''
4''	114.0, C		
5''	126.7, C		
6''	119.8, CH	7.36, s	6, 4'', 5'', 8'', 10''
7''	129.2,^d^ C		
8''	123.3, CH	7.62, dd (1.2, 8.6)	6, 6'', 10''
9''	109.7, CH	7.26, d (8.5)	5'', 7''
10''	135.3, C		
11''	122.4, CH	7.07, d (0.8)	3'', 4'', 5'', 10''
2-NH		7.15, d (7.6)	10
2''-NH		8.81, d (9.7)	1'
10''-NH		10.70, d (0.8)	4'', 5'', 10'', 11''

^a^Recorded at 125 MHz (reference δ_C_ 39.5 ppm). ^b^Recorded at 500 MHz (reference δ_H_ 2.50 ppm). ^c^HMBC correlations are from proton(s) stated to the indicated carbon. ^d^Overlapping signals.

**Figure 2:**
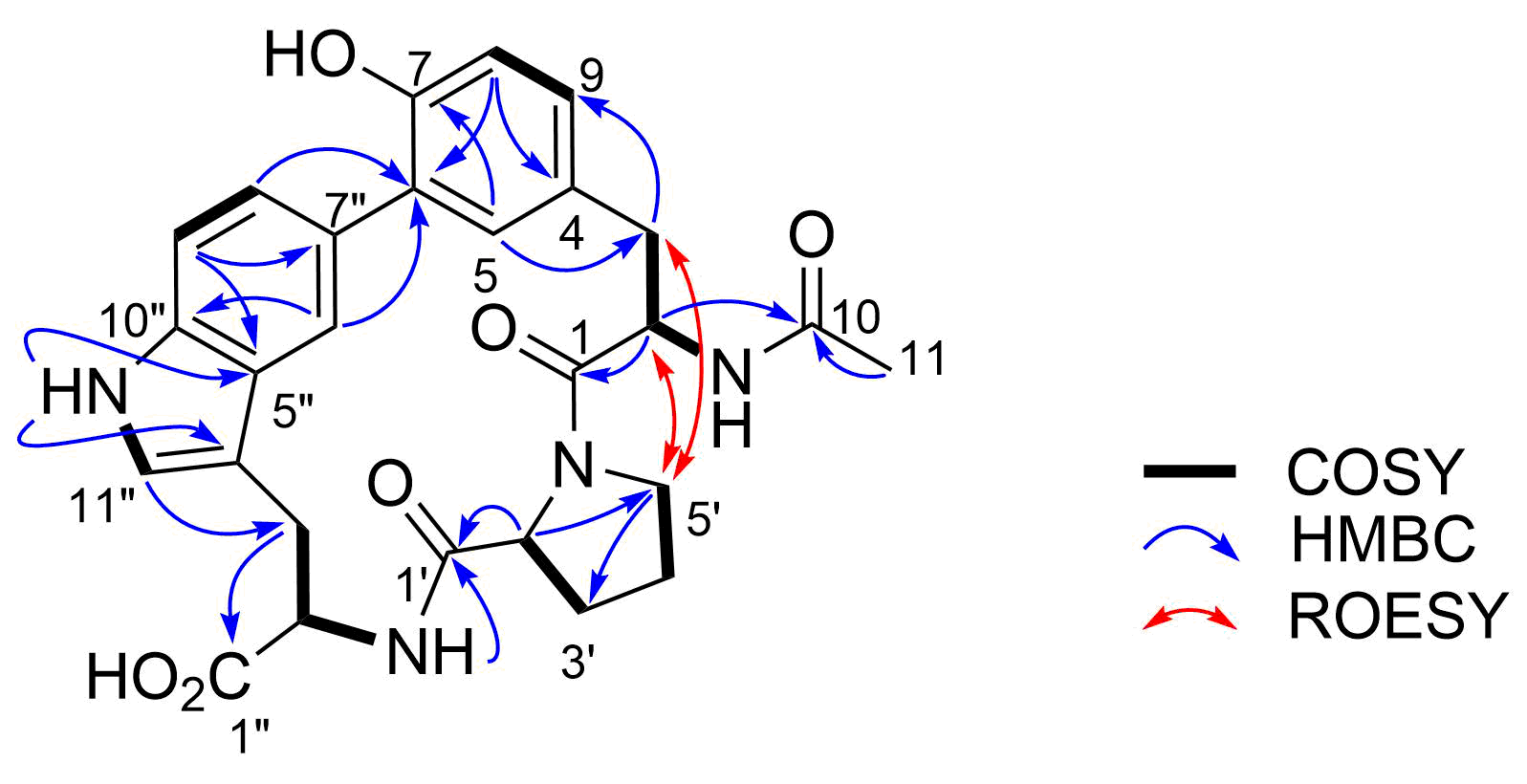
COSY, key HMBC and ROESY correlations of pseudosporamide (**1**).

The combined analysis of COSY and HMBC elucidated the presence of three amino acid residues, *N*-acetyltyrosine (AcTyr), proline (Pro), and tryptophane (Trp) (Figure 2). HMBC correlations from H-2' and H-3' to C-4' and from H-4' and H-5' to C-3' joined two COSY-defined fragments, H-2'/H-3' and H-4'/H-5'. Further HMBC correlations from H-2' to C-5' and C-1' (δ_C_ 171.1) connected C-2' and C-5' through a nitrogen atom and placed a carbonyl carbon next to C-2', establishing the Pro residue. In addition to these aliphatic portions, 14 aromatic carbons were assigned to one benzene and one indole ring by COSY and HMBC-based connectivity analysis. Protons H-8 and H-9 showed three-bond couplings (*J* = 8.2 Hz), while H-9 showed a weak four-bond correlation with H-5 (*J* = 1.9 Hz). Relatively intense cross peaks from H-5 and H-9 to the deshielded carbon C-7 (δ_C_ 152.8) suggested the oxygen functionality at the *meta*-position to C-5 and C-9. Intense HMBC cross peaks were also observed from H-8 to C-4 and C-6, indicating the *meta*-relationship among C-4, C-6, and C-8. The α-CH and β-CH_2_, whose connectivity was established by COSY spectrum analysis, were connected at C-4 on the basis of the HMBC correlations from H-5 and H-9 to C-3. The carbonyl carbon C-1 (δ_C_ 168.1) was confirmed by the long-range correlations from H-2 and H-3 to C-1. An acetamido group was then attached to C-2 on the basis of HMBC correlations from H-2, 2-NH, and H-11 to C-10 to complete the AcTyr residue, yet the connectivity from C-6 was not established at this moment.

A set of HMBC correlations from aromatic protons and an indole NH proton (δ_H_ 10.70) to the aromatic carbons established the indole unit (Figure 2, Table 1). C-7'' was not bearing a proton but was connected to C-6 of the AcTyr residue based on HMBC correlations from H-6'' and H-8'' to C-6. A COSY-defined fragment C-3''/C-2''/2''-NH was connected at C-4'' on the basis of the correlations from H-11'' to C-3'' and from H-3'' to C-5''. The connectivity between the carboxy carbon of Pro (C-1') and the amino group of Trp was confirmed by the HMBC correlations from H-2'' and 2''-NH to C-1'. Connectivity between the AcTyr and Pro residues was not proven by HMBC, but confirmed by ROESY correlations between H-5' and H-2/H-3 to give a planar structure of **1**.

**Figure 3:**
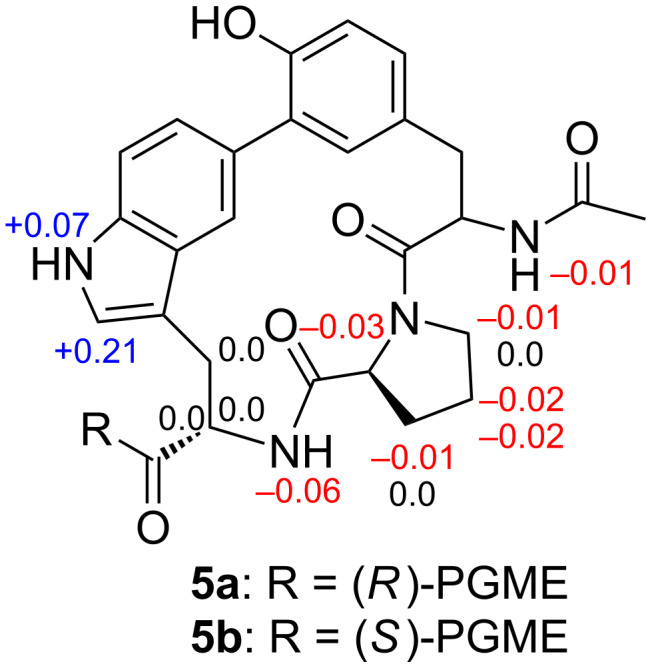
^1^H NMR Δδ*_S−R_* values for PGME amides **5a** and **5b** obtained from compound **1**.

The chirality of the Pro residue was determined by the advanced Marfey’s method. Compound **1** was acid-hydrolyzed and the hydrolysate was derivatized with 1-fluoro-2,4-dinitrophenyl-5-ʟ-leucinamide (L-FDLA). The chromatographical comparison of the reaction product with FDLA derivatives of authentic ʟ- and ᴅ-Pro [19–20], by reversed-phase LC–MS, revealed the elution of ʟ-Pro (Figure S8 in Supporting Information File 1). Thus, an (*S*)-configuration was concluded for C-2'. The absolute configuration of the Trp residue was determined using a chiral derivatizing reagent, phenylglycine methyl ester (PGME) [21]. Compound **1** was reacted with both enantiomers of PGME to give (*R*)- and (*S*)-PGME amides **5a** and **5b**, and the difference of the ^1^H NMR chemical shifts Δδ*_S_*_−_*_R_* was calculated around C2''. As positive Δδ*_S_*_−_*_R_* values were obtained for 10''-NH and H-11'', and negative values for 2''-NH, H-2', H-3', H-4', H-5', and 2-NH (Figure 3), an (*S*)-configuration was assigned to C-2''.

**Figure 4:**
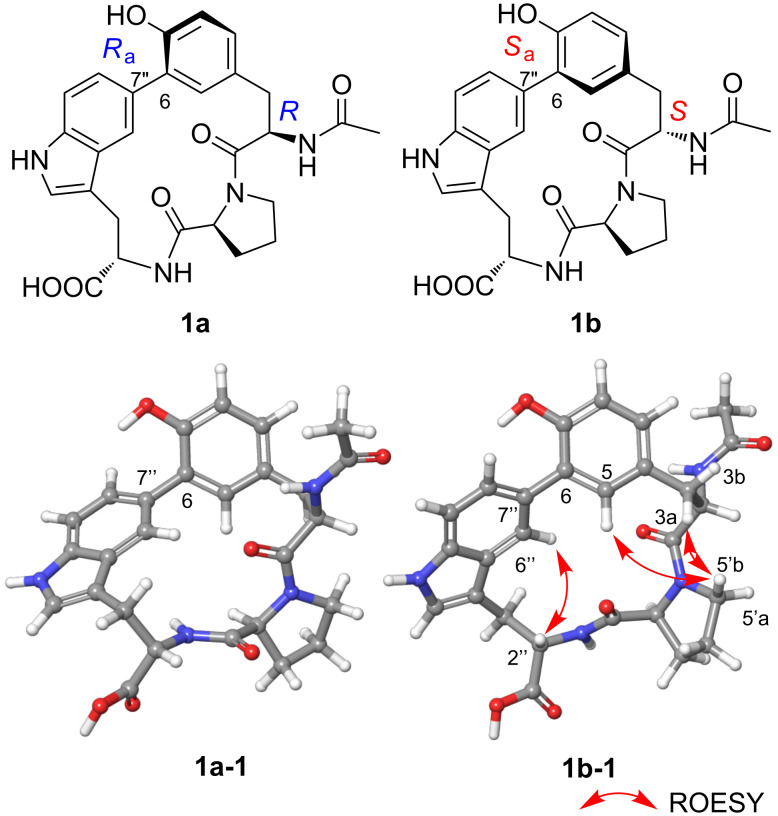
The opposite axial chirality around the biaryl C-6–C-7'' bond influenced by the C-2 configuration in compound **1**. 3D structures **1a-1** and **1b-1** are the most stable conformers of **1a** and **1b**, respectively.

**Figure 5:**
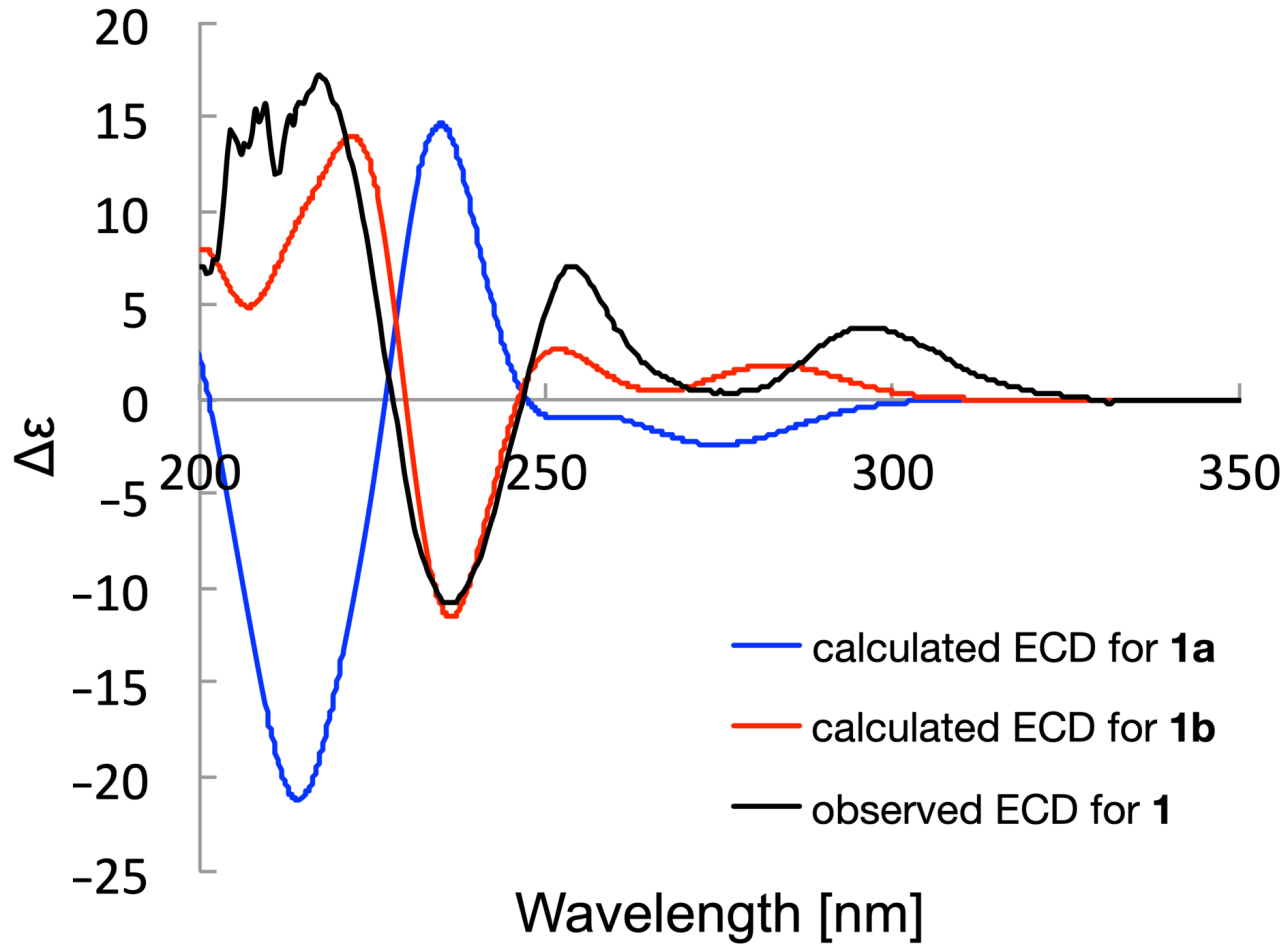
The experimental and calculated ECD spectra in MeCN.

The chirality of the AcTyr residue was investigated by computer-assisted conformational analysis and NMR chemical shift prediction [22–24]. ^1^H and ^13^C NMR chemical shifts were obtained for two possible diastereomers, (2*R*,2'*S*,2''*S*)-isomer (**1a**) and (2*S*,2'*S*,2''*S*)-isomer (**1b**), by calculation at the mPW1PW91/6-31G+(d,p)-PCM (DMSO) level of theory (Tables S1 and S2 in Supporting Information File 2). The experimental ^1^H and ^13^C NMR data of **1** presented a higher chemical shift similarity to those for **1b** and thereby the (*S*)-configuration was suggested for C-2. Indeed, the DP4+ analysis [25] gave 100% probability for structure **1b** and 0.0% probability for structure **1a** (Table S2, Supporting Information File 2). This result was consistent with the observation of a set of ROESY correlations for H-3a/H-5'b, H-5/H-5'b, and H-2''/H-6'' in the most stable conformer of (2*S*)-isomer **1b**, which were all incompatible with any conformers of the (2*R*)-isomer **1a** (Figure 4). Furthermore, the (2*S*)-configuration was supported by the comparison of experimental and simulated ECD spectra [26–27]. Quite interestingly, the DFT calculation suggested that **1a** and **1b** would possess the opposite axial chirality around the biaryl bond between C-6 and C-7'': *R*_a_ for **1a** and *S*_a_ for **1b** as dominant atropisomers (Figure 4). This conformational difference around the biaryl axis was likely caused by the steric repulsion between the acetamido group at C-2 and the H-5' methylene protons, by which the H-2 methine proton was directed in close proximity toward the H-5' protons. The experimental ECD spectrum of **1** displayed intense negative and positive Cotton effects at 236 nm and 217 nm, respectively, which was consistent with a Cotton effect pattern predicted for the *S*_a_-atropisomer of **1b**. Moreover, the overall spectral feature of the experimental and calculated ECDs were quite similar with each other (Figure 5). Based on these results, the absolute configuration of **1** was concluded to be 2*S*,2'*S*,2''*S*,6*S*_a_.

**Figure 6:**
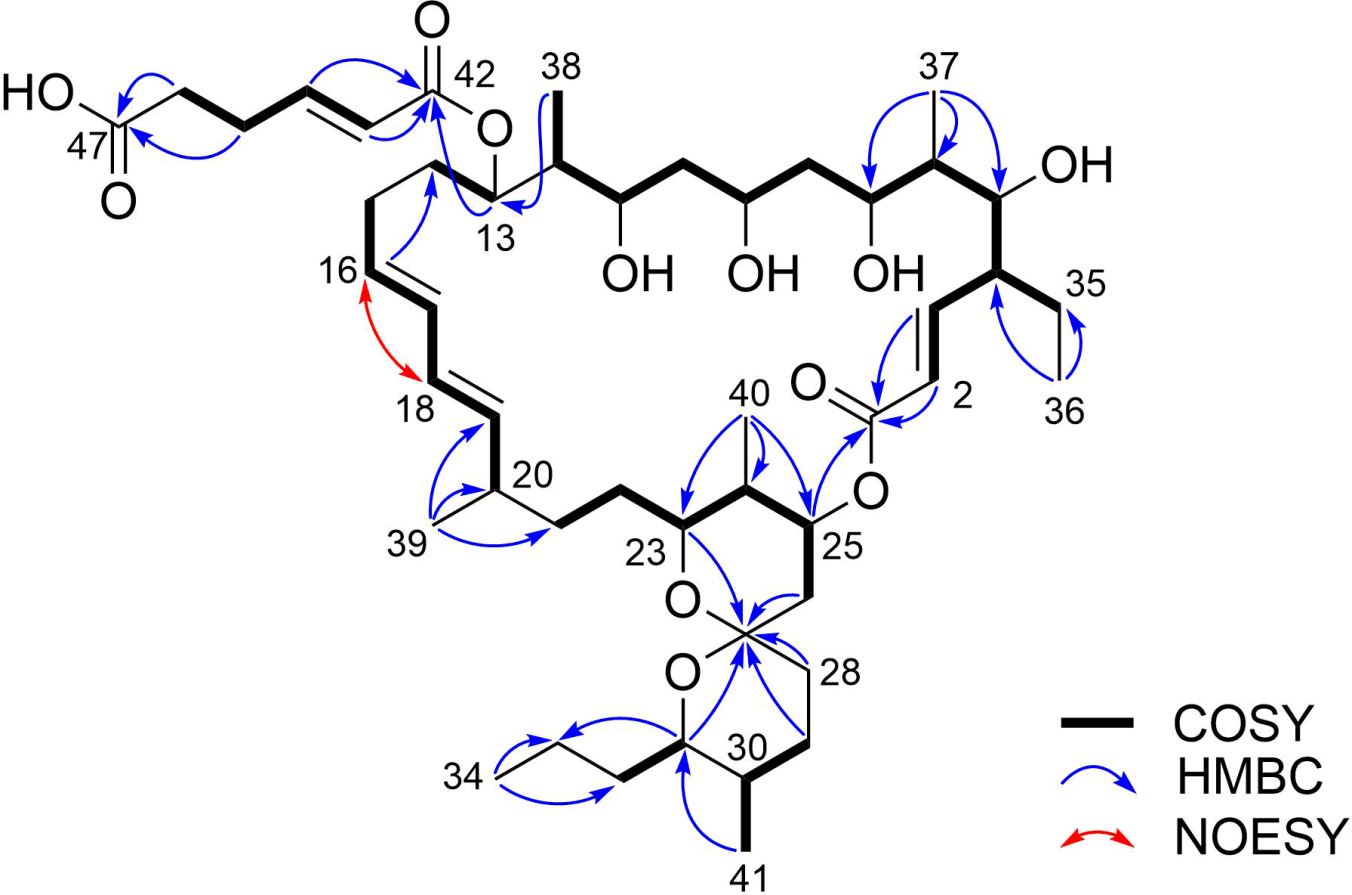
COSY, key HMBC and NOESY correlations of compound **2**.

Pseudosporamicin A (**2**) was obtained as white amorphous solid. An HRESITOFMS analysis gave a deprotonated molecular ion [M − H]^−^ at *m/z* 831.5255, which corresponded to the molecular formula having 10 degrees of unsaturation, C_47_H_76_O_12_. The ^1^H and ^13^C NMR resonances were sectionized into those at carboxy (δ_C_ 174.6–164.6), olefinic (δ_H_ 6.91–5.09; δ_C_ 148.9–122.3), acetal (δ_C_ 98.6), oxygenated (δ_H_ 5.40–3.66; δ_C_ 79.3–69.8), and aliphatic (δ_H_ 2.60–0.84; δ_C_ 48.9–3.9) regions, which obviously represented a characteristic feature of polyketides. The molecular components deduced from ^13^C NMR and HSQC spectra were three carbonyl carbons, eight olefinic methines, one acetal carbon, eight oxymethines, six aliphatic methines, 14 aliphatic methylenes, and seven aliphatic methyl groups (Table 2). The connectivity analysis of **2** started from the doublet olefinic methine H-2 which was correlated with another olefinic proton H-3 and a carbonyl carbon C-1 (δ_C_ 164.6) (Figure 6). This α,β-unsaturated carbonyl unit was extended to H-12 by sequential COSY correlations, providing a twelve-carbon chain from C-1 to C-12 with hydroxylation at the odd-numbered carbons (C-5, C-7, C-9, and C-11) and two methyl and one ethyl substituents on the even-numbered carbons (C-4, C-6, and C-12). Furthermore, via the two-carbon fragment C-13/C-14, a COSY-defined six-carbon chain from C-15 to C-20, containing a conjugated diene, was connected to C-12. Another six-carbon chain from C-21 to C-26 was then connected at C-20 by HMBC correlations from H_3_-39 to C-19, C-20, and C-21. The carbon chain was further extended from C-26, via the spiro carbon C-27 (δ_C_ 98.6) and a methylene C-29, to the terminal methyl C-34 on the basis of COSY and HMBC correlations. Finally, an HMBC correlation from the oxymethine H-25 to C-1 established a macrolactone structure, and a spiroacetal structure with two six-membered rings was deduced from a series of HMBC correlations of the protons in the spiro rings to C-27, illustrating the oligomycin-class structure. The remaining six carbons from C-42 to C-47 were assigned to constitute an (*E*)-2,3-dehydroadipic acid by COSY and HMBC analysis along with coupling constant analysis (*J*_H-43,H-44_ = 14.8 Hz), and the connection of this diacid portion to C-13 via an ester linkage completed the planar structure of compound **2**. An *E*,*E*-diene configuration for the C-16/C-17 and C-18/C-19 double bonds in *s*-*trans* conformation was determined by the NOESY correlation between H-16 and H-18 and the large vicinal coupling constants (*J*_H-16,H-17_ = 15.7 Hz, *J*_H-18,H-19_ = 14.9 Hz).

**Table 2:** NMR spectroscopic data for pseudosporamicin A–C (**2**–**4**) in CDCl_3_.

	**2**		**3**		**4**	
No.	δ_C_^a^, type	δ_H_, mult (*J* in Hz)^b^	δ_C_^a^, type	δ_H_, mult (*J* in Hz)^b^	δ_C_^a^, type	δ_H_, mult (*J* in Hz)^b^

1	164.6, C		164.7, C		164.8, C	
2	123.5, CH	5.75, d (15.8)	123.4, CH	5.75, d (15.8)	123.6, CH	5.76, d (16.2)
3	148.9, CH	6.48, dd (10.7, 15.8)	148.3, CH	6.48, dd (10.6, 15.8)	148.5, CH	6.48, dd (10.7, 16.2)
4	48.9, CH	2.16, m	49.0, CH	2.16, m	23.7c, CH	2.16, m
5	79.3, CH	3.74, m	79.5, CH	3.72, m	79.4, CH	3.73, m
6	40.7, CH	1.30, m	39.0, CH	1.36, m	39.6, CH	1.37, m
7	78.6, CH	4.11, m	77.7, CH	4.15, m	77.5, CH	4.15, m
8	42.0, CH_2_	1.23/1.69, m	41.9, CH_2_	1.33/1.67, m	41.9, CH_2_	1.36/1.66, m
9	75.1, CH	3.87, m	72.9, CH	4.03, m	72.7c, CH	4.01, m
10	37.0, CH_2_	1.35/1.78, m	39.2, CH_2_	1.49, m	39.4, CH_2_	1.50, m
11	73.3, CH	3.80, m	73.5, CH	3.34, m	73.4, CH	3.35, m
12	46.3, CH	1.75, m	46.1, CH	1.57, m	46.0^c^, CH	1.56, m
13	72.4, CH	4.64, m	75.0, CH	4.08, m	75.2, CH	4.04, m
14	32.1, CH_2_	1.60/1.82, m	33.9, CH_2_	1.76, m	33.8, CH_2_	1.41, m
15	28.7, CH_2_	2.08/2.22, m	28.3, CH_2_	2.26/2.29, m	28.2, CH_2_	2.25/2.31, m
16	129.6, CH	5.31, ddd (4.0, 11.0, 14.7)	130.4, CH	5.42, m	130.3, CH	5.40, m
17	132.5, CH	5.76, dd (10.7, 14.7)	132.3, CH	6.08, dd (10.7, 15.3)	132.5, CH	6.10, dd (10.7, 15.3)
18	129.3, CH	5.90, dd (10.7, 14.9)	128.9, CH	5.94, dd (10.5, 14.9)	130.4, CH	5.93, dd (10.7, 15.3)
19	137.9, CH	5.09, dd (9.6, 14.9)	139.1, CH	5.34, m	137.7, CH	5.25, dd (9.8, 15.3)
20	39.0, CH	2.10, m	38.3, CH	2.12, m	46.0^c^, CH	1.84, m
21	34.0, CH_2_	1.32/1.45, m	34.1^c^, CH_2_	1.38/1.41, m	32.3, CH_2_	1.41, m
22	30.7, CH_2_	1.10/1.68, m	31.0, CH_2_	1.07/1.57, m	31.0, CH_2_	1.04/1.58, m
23	71.6^c^, CH	3.85, m	69.6, CH	3.71, m	69.7, CH	3.72, m
24	35.8, CH	2.03, m	35.6, CH	2.04, m	35.8, CH	2.06, m
25	69.8, CH	5.40, dt (5.1, 11.9)	70.8, CH	5.31, m	71.2, CH	5.31, m
26	35.8, CH_2_	1.81/1.86, m	30.0, CH_2_	1.46/1.48, m	35.6, CH_2_	1.71, m
27	98.6, C		97.4, C		97.4, C	
28	29.0, CH_2_	1.69/1.77, m	34.1^c^ CH_2_	1.67/1.79, m	30.0, CH_2_	1.47, m
29	26.5, CH_2_	1.41/2.04, m	26.7, CH_2_	1.40/2.08, m	26.7, CH_2_	2.08, m
30	30.0, CH	1.64, m	30.3, CH	1.62, m	30.2, CH	1.61, m
31	71.6^c^, CH	3.66, m	71.2, CH	3.65, m	72.7^c^, CH	3.65, m
32	35.5, CH_2_	1.26/1.45, m	35.8, CH_2_	1.24/1.68, m	35.6, CH_2_	1.25/1.68, m
33	19.9, CH_2_	1.27/1.52, m	20.0, CH_2_	1.25/1.50, m	20.0, CH_2_	1.50, m
34	14.5, CH_3_	0.94, t (6.9)	14.5, CH_3_	0.94, t (7.0)	14.5, CH_3_	0.94, t (6.8)
35	23.6, CH_2_	1.23, 1.98, m	23.7, CH_2_	1.23/1.98, m	23.7^c^, CH_2_	1.22/1.98, m
36	9.9, CH_3_	0.84^d^	11.7, CH_3_	0.84^d^	11.7, CH_3_	0.84^d^
37	3.9, CH_3_	0.84^d^	3.8, CH_3_	0.85, d (5.8)	3.8, CH_3_	0.84^d^
38	11.7, CH_3_	0.88, d (6.8)	11.3, CH_3_	0.80, d (6.9)	11.5, CH_3_	0.80, d (6.8)
39	22.2, CH_3_	0.98, d (6.7)	21.8, CH_3_	1.01, d (6.7)	28.9, CH_2_	1.40, m
40	5.1, CH_3_	0.84^d^	5.4, CH_3_	0.72, d (6.9)	5.4, CH_3_	0.71, d (6.8)
41	11.1, CH_3_	0.94, d (7.0)	11.1, CH_3_	0.90, d (7.0)	11.1, CH_3_	0.90, d (7.0)
42	165.6, C				12.1, CH_3_	0.84^d^
43	122.3, CH	5.90, d (14.7)				
44	147.3, CH	6.91, m				
45	27.8, CH_2_	2.52/2.60, m				
46	33.4, CH_2_	2.44/2.53, m				
47	174.6, C					

^a^Recorded at 125 MHz (reference δ_C_ 77.2 ppm). ^b^Recorded at 500 MHz (reference δ_H_ 7.26 ppm). ^c^Overlapping signals. ^d^Coupling constants could not be determined due to signal overlapping.

**Figure 7:**
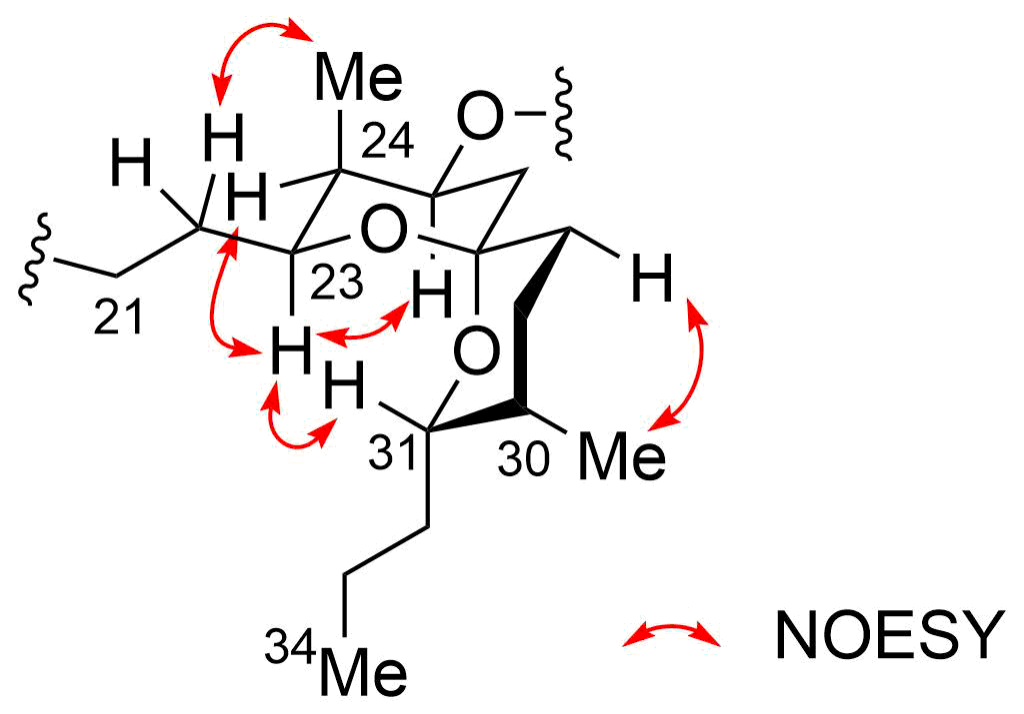
NOESY correlations for the spiroacetal moiety of compound **2**.

The relative configuration of the spiroacetal moiety in **2** was determined by NOESY analysis. Correlations between H-23/H-24, H-23/H-25, and H-40/H-22 established the axial positioning of H-23 and H-25 and the placement of H-40 methyl group on the opposite side of the chair-conformation ring. Additional correlations for H-23/H-31 and H-41/H-28_ax_ unequivocally confirmed the chair conformation of another six-membered ring and the axial orientations of H-31 and the H-41 methyl group. Therefore, the relative configuration of the spiroacetal ring was established to be (23S*,24R*,25S*,27R*,30S*,31S*) (Figure 7).

The molecular formula of pseudosporamicin B (**3**) was determined to be C_41_H_70_O_9_ based on a deprotonated molecular ion [M − H]^−^ at *m/z* 705.4942 in the HRESITOFMS analysis, which was six carbon, six hydrogen, and four oxygen atoms less compared to compound **2**. In the ^1^H and ^13^C NMR spectra, resonances for dehydroadipate, the acyl side chain part at C-13 were missing, whereas all other resonances were similarly observed, except for the more shielded H-13 with a chemical shift of 4.08 ppm (as compared to 4.64 ppm for **2**) due to the lack of an electron-withdrawing group. Thus, **3** was determined as a deacylation congener of **2** at C-13 (Table 2, Figure S19 in Supporting Information File 1).

The NMR spectra of pseudosporamicin C (**4**) were closely similar to those of **3** in overall (Table 2, Figure S26 in Supporting Information File 1). One noticeable difference was the presence of an ethyl group at C-20 instead of a methyl group, which was deduced from the HMBC correlations from a triplet methyl H-42 to C-20 and C-39. This one carbon increment was consistent with the result from HRESITOFMS analysis which gave a deprotonated molecular ion [M − H]^−^ at *m*/*z* 719.5101, corresponding to the molecular formula C_42_H_71_O_9_. Therefore, **4** was identified as a 20-ethyl congener of compound **3**.

The bioactivities of compounds **1**–**4** were examined in antimicrobial and cytotoxicity assays (Table 3). Pseudosporamide (**1**) was not active against all microorganisms tested in this study. Pseudosporamicin **2**–**4** were selectively active against the Gram-positive bacterium *Kocuria rhizophila* and the filamentous fungus *Glomerella cingulate,* but were inactive against *Staphylococcus aureus*, *Escherichia coli*, *Rhizobium radiobacter*, and *Candida albicans*. The antimicrobial potency of **2** and **3** were similar, implying that the acyl side chain at C13 of compound **2** had no influence on the activity. In addition, compounds **1**–**4** showed moderate cytotoxicity against P388 murine leukemia cells with IC_50_ values of 11, 0.12, 0.35, and 3.7 μM, respectively.

**Table 3:** Antimicrobial activity of pseudosporamide (**1**) and pseudosporamicin A–C (**2**–**4**).

	MIC (μg/mL)			

microorganism	**1**	**2**	**3**	**4**

*Kocuria rhizophila* ATCC9341	>100	0.78	0.78	6.25
*Staphylococcus aureus* FDA209P JC-1	>100	>100	>100	>100
*Escherichia coli* NIHJ JC-2	>100	>100	>100	>100
*Rhizobium radiobacter* NBRC14554	>100	>100	>100	>100
*Candida albicans* NBRC0197	>100	50	100	>100
*Glomerella cingulata* NBRC5907	>100	0.78	0.78	1.56

## Conclusion

**Figure 8:**
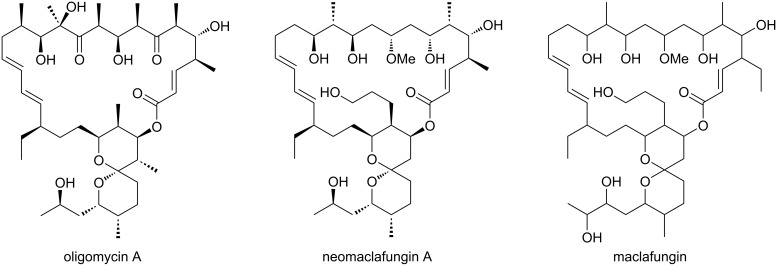
Selected examples of oligomycin-class metabolites from actinomycetes.

Many unique features differentiate compounds **1**–**4** from known metabolites: a tripeptide sequence Tyr–Pro–Trp in **1** is shared with several opiate class endogeneous neuropeptides [28], but not with secondary metabolites; the biaryl bonding between Tyr and Trp is only precedented by kistamicin A, a vancomycin-class glycopeptide produced by an actinomycete of the genus *Nonomuraea* (family *Streptosporangiaceae*) [29–30] and TMC95A–D from an ascomycetous fungus *Apiospora montagnei* [31]; the (*E*)-2,3-dehydroadipyl appendage in compound **2** is unprecedented in natural products, only to see its (*Z*)-isomer in a siderophore of *Mycobacterium avium* [32]; the side chain on the spiroacetal rings in compounds **2**–**4** lack hydroxylation at C-31, which is the common modification shared with all oligomycin class antibiotics (oligomycin [33], dunaimycins [34], rutamycins [35], ossamycin [36], neomaclafungins [37], and IB-96212 [38]) and related polyketides with ring-truncation (ushikulides [39], yokonolides [40–41], A59770A [42], dunaimycins [34]) or expansion (neaumycins [43]) (Figure 8). All these compounds, except for maclafungin [44], the producer of which was not identified, are metabolites of *Streptomyces* [33–36] or conventional rare actinomycetes [37–38].

Thus, a novel skeleton and new congeners with distinct structural features were discovered from an underexplored actinomycete of the genus *Pseudosporangium*, which was chosen with the aid of a database survey on the history of chemical studies and the presence of biosynthetic genes. The same approach is also applicable to other natural product resources and should help discover metabolites with higher degree of novelty, provided that support from bioinformatics and culture collections are available.

## Experimental

### General experimental procedures

Optical rotations were measured using a JASCO DIP-3000 polarimeter. The UV spectra were recorded on a Hitachi U-3210 spectrophotometer. The IR spectra were measured on a PerkinElmer Spectrum 100. NMR spectra were obtained on a Bruker AVANCE 500 spectrometer in DMSO-*d*_6_ or CDCl_3_, and referenced to the residual solvent signals (δ_H_ 2.50, δ_C_ 39.5 for DMSO-*d*_6_; δ_H_ 7.26, δ_C_ 77.2 for CDCl_3_). HRESITOFMS spectra were recorded on a Bruker micrOTOF spectrometer.

### Microorganism

*Pseudosporangium* sp. RD062863 was obtained from NBRC (Biological Resource Center, National Institute of Technology and Evaluation, Chiba, Japan). The strain was identified as a member of the genus *Pseudosporangium* on the basis of 99.7% similarity in the 16S rRNA gene sequence (1428 nucleotides; GenBank accession number LC512747) to *Pseudosporangium ferrugineum* strain 3-44-a(19)^T^ (AB302183).

### Fermentation

Strain RD062863 growing on double-diluted ISP2 agar medium consisting of yeast extract 0.2%, malt extract 0.5%, glucose 0.2%, and agar 2% (pH 7.3) was inoculated into test tubes (inner diameter, 15 mm; length 16.5 cm) each containing 5 mL of YG seed medium consisting of glucose 1% and yeast extract 1% (pH 7.0). The tubes were placed in a shaker TC-500R (Takasaki Scientific Instruments Corp.) (260 strokes/min) at 28 °C for 4 days. Then, the seed culture (1 mL) was transferred into 500 mL Erlenmeyer flasks each containing 100 mL of modified A3M production medium consisting of glucose 0.5%, glycerol 2%, soluble starch 2%, Pharmamedia (Traders Protein) 1.5%, dry yeast (Kamaishi Hamayuri Yeast, Nihon Tensai Seito Co., Ltd.) 0.3%. The pH of the medium was adjusted to 7.0 before sterilization. The inoculated flasks were placed on a rotary shaker TB98 (Takasaki Scientific Instruments Corp.) (120 rpm) at 30 °C for 6 days.

### Isolation

At the end of fermentation, 100 mL of 1-butanol were added to each flask, and the flasks were allowed to shake for 1 h. The mixture was centrifuged at 6,000 rpm for 10 min and the organic layer was separated from the aqueous layer containing the mycelium. The organic layer was concentrated in vacuo to give 14.6 g of extract from 6 L culture. The crude extract was subjected to silica gel column chromatography with a gradient of a CHCl_3_/MeOH mixture as eluent (1:0, 20:1, 10:1, 4:1, 2:1, 1:1, and 0:1 v/v). After evaporation, the fraction 5 (2:1) was further fractionated by ODS column chromatography with a gradient of MeCN/0.1% HCO_2_H aqueous solution (2:8, 3:7, 4:6, 5:5, 6:4, 7:3, and 8:2 v/v). The ODS fraction 2 (3:7) was evaporated to yield pseudosporamide (**1**, 16 mg). The fraction 3 (10:1) of silica gel column chromatography was evaporated and fractionated by ODS column chromatography with a gradient of MeCN/0.1% HCO_2_H solution (2:8, 3:7, 4:6, 5:5, 6:4, 7:3, 8:2 v/v, and MeOH). The fraction 8 (MeOH) was evaporated, and final purification was achieved by preparative HPLC using an isocratic condition of MeCN/0.1% HCO_2_H solution (MeCN concentration: 85% over 0–90 min) at 4 mL/min, yielding pseudosporamicin A (**2**, 11 mg, *t*_R_ 44.4 min), pseudosporamicin B (**3**, 9.8 mg, *t*_R_ 56.0 min), and pseudosporamicin C (**4**, 16.0 mg, *t*_R_ 77.0 min).

Pseudosporamide (**1**): white amorphous solid; [α]_D_^25^ −5.6 (*c* 0.08, MeOH); UV (MeOH) λ_max_ (log ε) 202 (2.47), 252 (1.53), 295 (0.48) nm; ECD (1 × 10^−4^ M, MeCN) λ_ext_ (Δε) 217.0 (+17.3), 235.5 (−10.8) nm; IR (ATR) ν_max_ 3328, 1722 cm^−1^; for ^1^H and ^13^C NMR data, see Table 1; HRESITOFMS (*m/z*): [M − H]^−^ calcd for C_27_H_27_N_4_O_6_, 503.1931; found, 503.1936.

Pseudosporamicin A (**2**): white amorphous powder; [α]_D_^25^ −23 (*c* 0.50, MeOH); UV (MeOH) λ_max_ (log ε) 218 (4.65) nm; IR (ATR) ν_max_ 3366, 1713, 1635 cm^−1^; for ^1^H and ^13^C NMR data, see Table 2; HRESITOFMS (*m/z*) [M − H]^−^ calcd for C_47_H_75_O_12_, 831.5259; found, 831.5255.

Pseudosporamicin B (**3**): white amorphous powder; [α]_D_^25^ −9.3 (*c* 0.50, MeOH); UV (MeOH) λ_max_ (log ε) 223 (4.56) nm; IR (ATR) ν_max_ 3385, 1716, 1643 cm^−1^; for ^1^H and ^13^C NMR data, see Table 2; HRESITOFMS (*m/z*): [M − H]^−^ calcd for C_41_H_69_O_9_, 705.4942; found, 705.4942.

Pseudosporamicin C (**4**): white amorphous powder; [α]_D_^25^ −10 (*c* 0.05, MeOH); UV (MeOH) λ_max_ (log ε) 224 (4.78) nm; IR (ATR) ν_max_ 3378, 1716, 1642 cm^−1^; for ^1^H and ^13^C NMR data, see Table 2; HRESITOFMS (*m/z*): [M − H]^−^ calcd for C_42_H_71_O_9_, 719.5098; found, 719.5101.

### Marfey’s analysis

In a similar manner as described in [45], a portion of **1** (0.1 mg) was hydrolyzed at 110 °C with 6 M HCl (200 μL) for 16 h, and the reaction mixture was concentrated to dryness. A 0.1 M NaHCO_3_ solution (100 μL) was added to the dried hydrolysate of **1**, as well as to the standard of ʟ- and ᴅ-proline (Pro). A solution of ʟ-FDLA in acetone (0.05 mg in 50 μL) was added to each reaction vial. Each vial was sealed and incubated at 50 °C for 3 h. To quench reactions, 2 M HCl (50 μL) was added and then diluted with MeCN/0.2% HCO_2_H aqueous solution (100 μL, 50:50). The Marfey’s derivatives of the hydrolysate and standards were analyzed by LC–MS using a Cosmosil 5C18-AR-II column eluted with MeCN/0.1% HCO_2_H aqueous solution at a flow rate of 1.0 mL/min, monitoring at 340 nm with a linear gradient of MeCN from 15% to 85% over 30 min. Retention times for the amino acid standards were 20.1 min for ʟ-Pro-ʟ-FDLA, 21.1 min for ᴅ-Pro-ʟ-FDLA, while the ʟ-FDLA-hydrolysate of **1** gave a peak at 20.1 min (Figure S8 in Supporting Information File 1).

### (*R*)- and (*S*)-PGME amides of **1** (**5a** and **5b**)

To a solution of **1** (1.0 mg, 2.0 μmol) in dry DMF (100 μL) and *N*,*N*-diisopropylethylamine (10 μL) were added (*R*)-phenylglycine methyl ester [(*R*)-PGME, 1.6 mg, 7.9 μmol], PyBOP (3.8 mg, 7.3 μmol), and HOBt (1.0 mg, 7.4 μmol) at room temperature. After stirring for 3 h, ice-water was added to the reaction mixture, which was then extracted with EtOAc. After removing the solvent, the residue was purified on a silica gel thin-layer plate (Kieselgel 60F_254_; Merck Co.) developed by a mixture of CHCl_3_/MeOH 1:1. Extraction of the collected fraction with MeOH and evaporation gave (*R*)-PGME amide **5a** (0.6 mg): ^1^H NMR (500 MHz, DMSO-*d*_6_) δ 4.67 (H-2'), 1.80 (H-3'a), 2.10 (H-3'b), 1.94 (H-4'a), 2.04 (H-4'b), 3.65 (H-5'a), 3.70 (H-5'b), 4.68 (H-2''), 2.81 (H-3''a), 3.35 (H-3''b), 6.91 (H-11''), 7.16 (2-NH), 9.07 (2''-NH), 10.67 (10''-NH); HRESITOFMS (*m/z*): [M − H]^−^ calcd for C_36_H_36_N_5_O_7_, 650.2615; found, 650.2612.

In the same manner as described for compound **5a**, **5b** was prepared from **1** and (*S*)-PGME: ^1^H NMR (500 MHz, DMSO-*d*_6_) δ 4.63 (H-2'), 1.80 (H-3'a), 2.09 (H-3'b), 1.92 (H-4'a), 2.02 (H-4'b), 3.64 (H-5'a), 3.70 (H-5'b), 4.68 (H-2''), 2.81 (H-3''a), 3.35 (H-3''b), 7.12 (H-11''), 7.15 (2-NH), 9.01 (2''-NH), 10.74 (10''-NH); HRESITOFMS (*m/z*): [M − H]^−^ calcd for C_36_H_36_N_5_O_7_, 650.2615; found, 650.2614.

### Computational procedure

#### General information

Conformational search was performed with MacroModel implemented in the Maestro 11.7 software package [46–47]. All DFT-based calculations were performed with the Gaussian 16 Rev B.01 program [48]. A part of these computations was conducted using the SuperComputer System, Institute for Chemical Research, Kyoto University. Molecular structures were visualized using the Maestro 11.7 software package. DP4+ analysis was performed with the Excel spreadsheet made by Sarotti et al. [25]. ECD spectra were visualized using GaussView 6.0.16 and Microsoft Excel. Cartesian coordinates of the structures described in this paper are included in Supporting Information File 2.

#### Procedure for the optimization, energy evaluation and simulation of the NMR and ECD spectra for structures **1a** and **1b**

The conformational search on structure **1a** began by applying 100,000 steps of the Monte-Carlo Multiple Minimum (MCMM) method with PRCG energy minimization using the OPLS3e force field (gas phase) to obtain 20 conformational isomers within 5.0 kcal/mol from the minimum energy conformer. The next optimizations were performed at the B3LYP/6-31G(d) level of theory. Frequency calculations were carried out at the same level of theory to confirm the absence of imaginary frequencies and to obtain thermal corrections to the Gibbs free energies at 1 atm, 298.15 K. The duplicate structures with RMSDs of 0.01 Å were removed. To evaluate the Boltzmann's population in the NMR solvent, single-point energies were calculated at the M06-2X/6-311+G(d,p) level of theory and solvation effects were included using the PCM solvation model (DMSO). The NMR chemical shifts were simulated by GIAO method at the mPW1PW91/6-31G+(d,p)-PCM(DMSO) level of theory. The chemical shifts (δ_calc_) were calculated using tetramethylsilane (TMS) as a reference standard according to δ_calc_ = σ_0_ − σ*_x_*, where σ*_x_* is the Boltzmann-averaged shielding tensor of the most stable 6 conformers within 3.0 kcal/mol and σ_0_ is the shielding tensor of TMS calculated at the same level of theory with σ*_x_*. For the ECD calculation, single-point energies were recalculated at the M06-2X/6-311+G(d,p) level of theory and solvation effects were included using the PCM solvation model (MeCN) to afford 6 conformers within 3.0 kcal/mol from the minimum energy. The ECD spectra of the 6 structures were simulated by the calculation of 25 states using TD-DFT at the ωB97X-D/Def2-TZVP-PCM(MeCN) level of theory, and then averaged based on their Boltzmann distribution. The calculated ECD spectra were red-shifted by 15 nm. The NMR and ECD spectra of structure **1b** were similarly simulated by using 44 structures as the OPLS3e-minimized structures and 7 conformers as the DFT-optimized structures.

### Antimicrobial assay

The antimicrobial assay was carried out in the same manner as reported previously [49]. MICs of reference antibiotics, kanamycin and amphotericin B, were 0.31 (against *K. rhizophila*) and 0.13 (against *G. cingulata*) μg/mL, respectively.

### Cytotoxicity assay

The cytotoxicity assay was carried out in the same manner as reported previously [49]. The IC_50_ of the reference drug doxorubicin was 0.13 μM.

## Supporting Information

File 1 Copies of NMR spectra for compounds **1**–**4**.

File 2 Copies of NMR chemical shifts calculation for compound **1**.
