# Supplementary material for: A cyclopeptide and three oligomycin-class polyketides produced by an underexplored actinomycete of the genus Pseudosporangium
Source: Beilstein J Org Chem. 2020 May 25;16:1100–10. doi: 10.3762/bjoc.16.97 (PMC7277628; doi:10.3762/bjoc.16.97)
Supplement: File 1 — Copies of NMR spectra for compounds 1–4. [file Beilstein_J_Org_Chem-16-1100-s001.pdf]

BEILSTEIN JOURNAL OF ORGANIC CHEMISTRY

Supporting Information

for

A cyclopeptide and three oligomycin-class polyketides produced by an underexplored actinomycete of the genus *Pseudosporangium*

Shun Saito, Kota Atsumi, Tao Zhou, Keisuke Fukaya, Daisuke Urabe, Naoya Oku, Md. Rokon Ul Karim, Hisayuki Komaki and Yasuhiro Igarashi

Beilstein J. Org. Chem. **2020,** *16,* 1100–1110. doi:10.3762/bjoc.16.97

Copies of NMR spectra for compounds 1–4

License and Terms: This is a supporting information file under the terms of the Creative Commons Attribution License (http://creativecommons.org/licenses/by/4.0). Please note that the reuse, redistribution and reproduction in particular requires that the authors and source are credited.
The license is subject to the *Beilstein Journal of Organic Chemistry* terms and conditions: (https://www.beilstein-journals.org/bjoc)

Table of contents

Figure S1. ^{1}H NMR spectrum of pseudosporamide (**1**) (500 MHz, DMSO-d_6) S3
Figure S2. ^{13}C NMR spectrum of **1** (125 MHz, DMSO-d_6) S4
Figure S3. DEPT135 spectrum of **1** (125 MHz, DMSO-d_6) S5
Figure S4. ^{1}H-^{1}H COSY spectrum of **1** (500 MHz, DMSO-d_6) S6
Figure S5. HSQC spectrum of **1** (500 MHz, DMSO-d_6) S7
Figure S6. HMBC spectrum of **1** (500 MHz, DMSO-d_6) S8
Figure S7. ROESY spectrum of **1** (500 MHz, DMSO-d_6) S9
Figure S8. HPLC chromatogram of hydrolysate from **1** and comparison to standard amino acids, derivatized with Marfey's reagent (FDLA) S10
Figure S9. ^{1}H NMR spectrum of **5a** (500 MHz, DMSO-d_6) S11
Figure S10. ^{1}H-^{1}H COSY spectrum of **5a** (500 MHz, DMSO-d_6) S12
Figure S11. ^{1}H NMR spectrum of **5b** (500 MHz, DMSO-d_6) S13
Figure S12. ^{1}H-^{1}H COSY spectrum of **5b** (500 MHz, DMSO-d_6) S14
Figure S13. ^{1}H NMR spectrum of pseudosporamicin A (**2**) (500 MHz, $CDCl_3$) S15
Figure S14. ^{13}C NMR spectrum of **2** (125 MHz, $CDCl_3$) S16
Figure S15. ^{1}H-^{1}H COSY spectrum of **2** (500 MHz, $CDCl_3$) S17
Figure S16. HSQC spectrum of **2** (500 MHz, $CDCl_3$) S18
Figure S17. HMBC spectrum of **2** (500 MHz, $CDCl_3$) S19
Figure S18. NOESY spectrum of **2** (500 MHz, $CDCl_3$) S20
Figure S19. ^{1}H NMR spectrum of pseudosporamicin B (**3**) (500 MHz, $CDCl_3$) S21
Figure S20. ^{13}C NMR spectrum of **3** (125 MHz, $CDCl_3$) S22
Figure S21. ^{1}H-^{1}H COSY spectrum of **3** (500 MHz, $CDCl_3$) S23
Figure S22. HSQC spectrum of **3** (500 MHz, $CDCl_3$) S24
Figure S23. HMBC spectrum of **3** (500 MHz, $CDCl_3$) S25
Figure S24. NOESY spectrum of **3** (500 MHz, $CDCl_3$) S26
Figure S25. ^{1}H NMR spectrum of pseudosporamicin C (**4**) (500 MHz, $CDCl_3$) S27
Figure S26. ^{13}C NMR spectrum of **4** (125 MHz, $CDCl_3$) S28
Figure S27. ^{1}H-^{1}H COSY spectrum of **4** (500 MHz, $CDCl_3$) S29
Figure S28. HSQC spectrum of **4** (500 MHz, $CDCl_3$) S30
Figure S29. HMBC spectrum of **4** (500 MHz, $CDCl_3$) S31
Figure S30. NOESY spectrum of **4** (500 MHz, $CDCl_3$) S32
Figure S31. COSY, key HMBC and NOESY correlations of **3** and **4** S33

Figure S1. ^{1}H NMR spectrum of pseudosporamide (**1**) (500 MHz, DMSO-d_6).

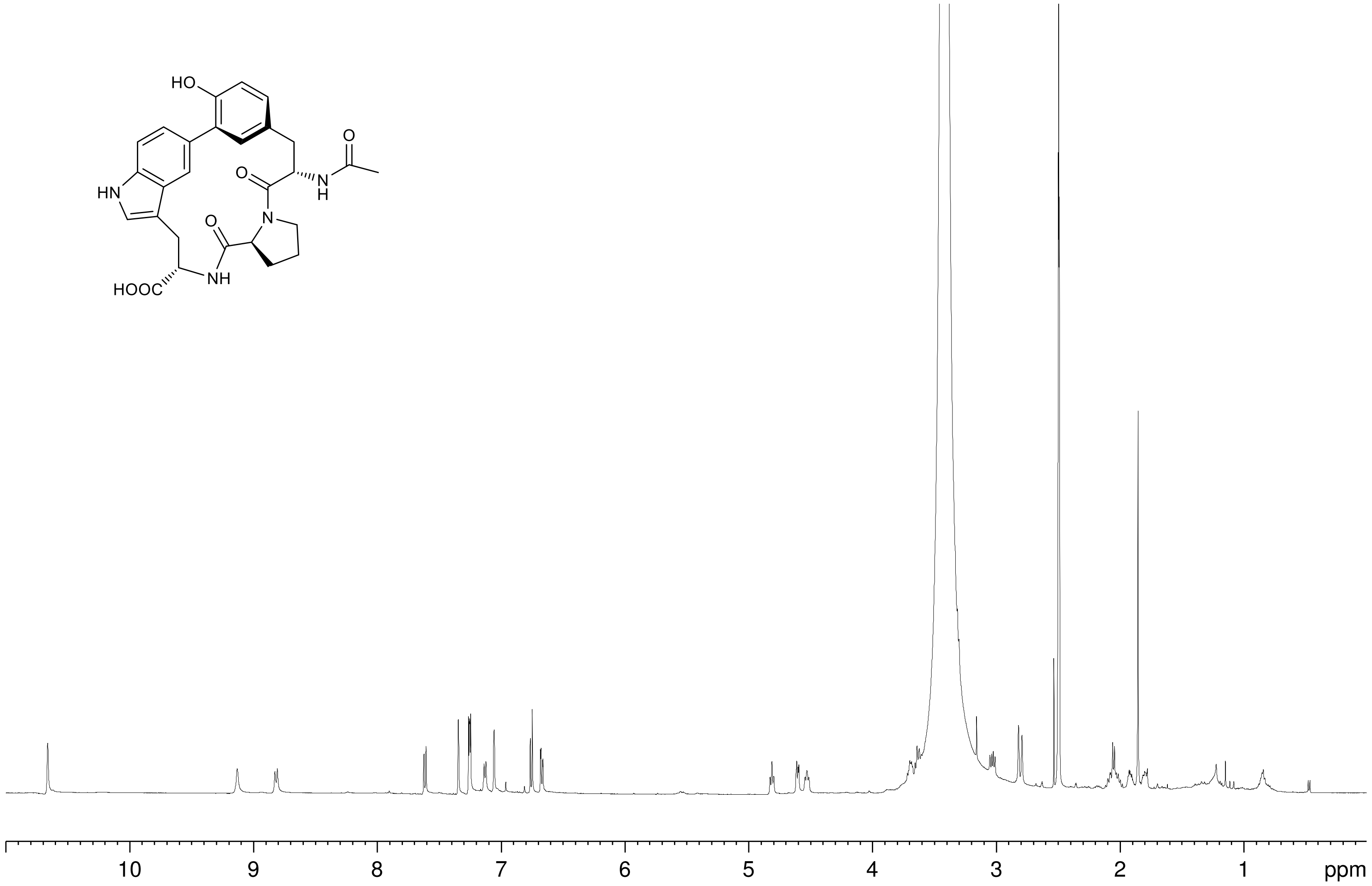

Figure S2. ^{13}C NMR spectrum of **1** (125 MHz, DMSO-d_6).

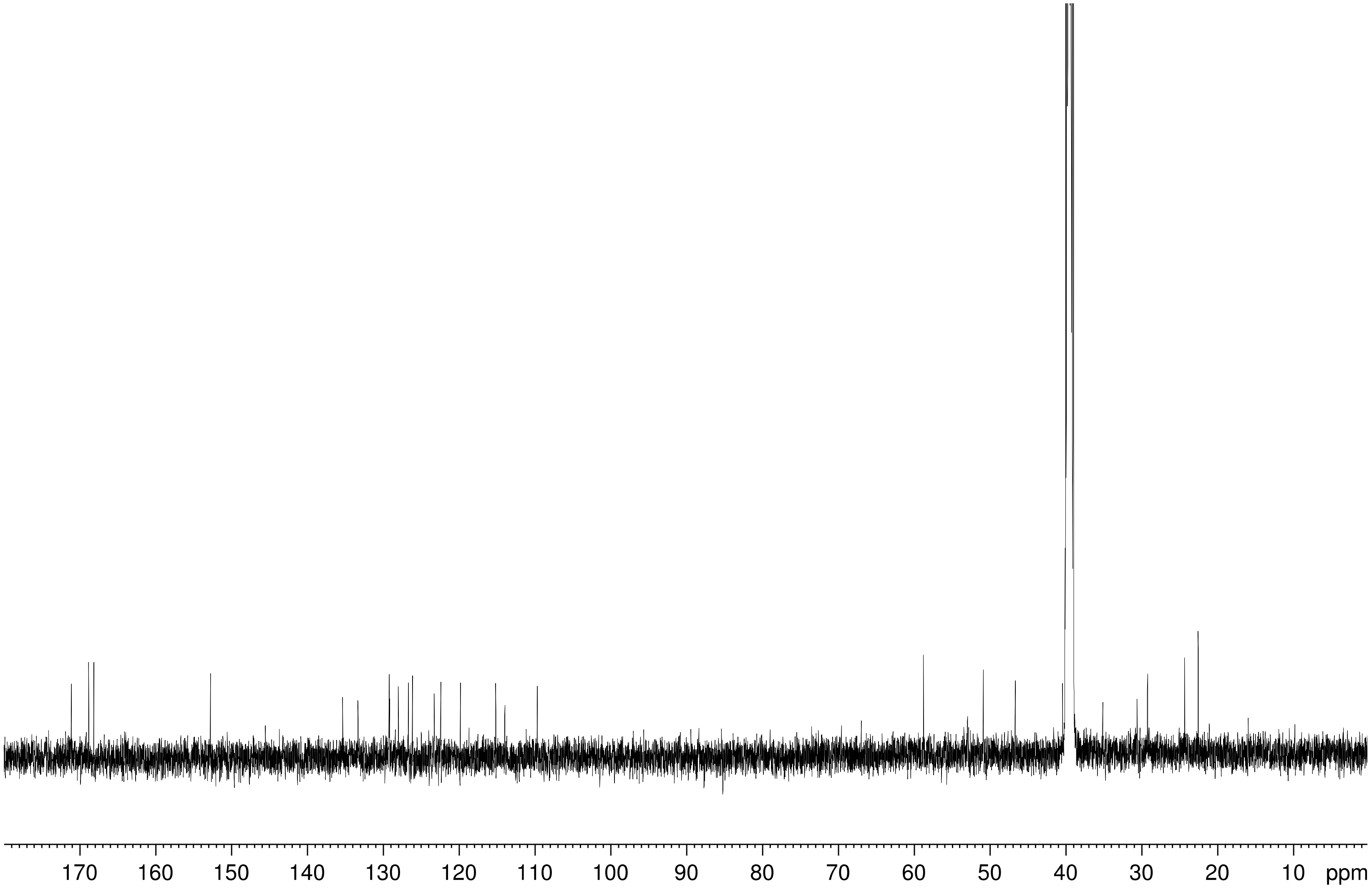

Figure S3. DEPT135 spectrum of **1** (125 MHz, DMSO-d_6).

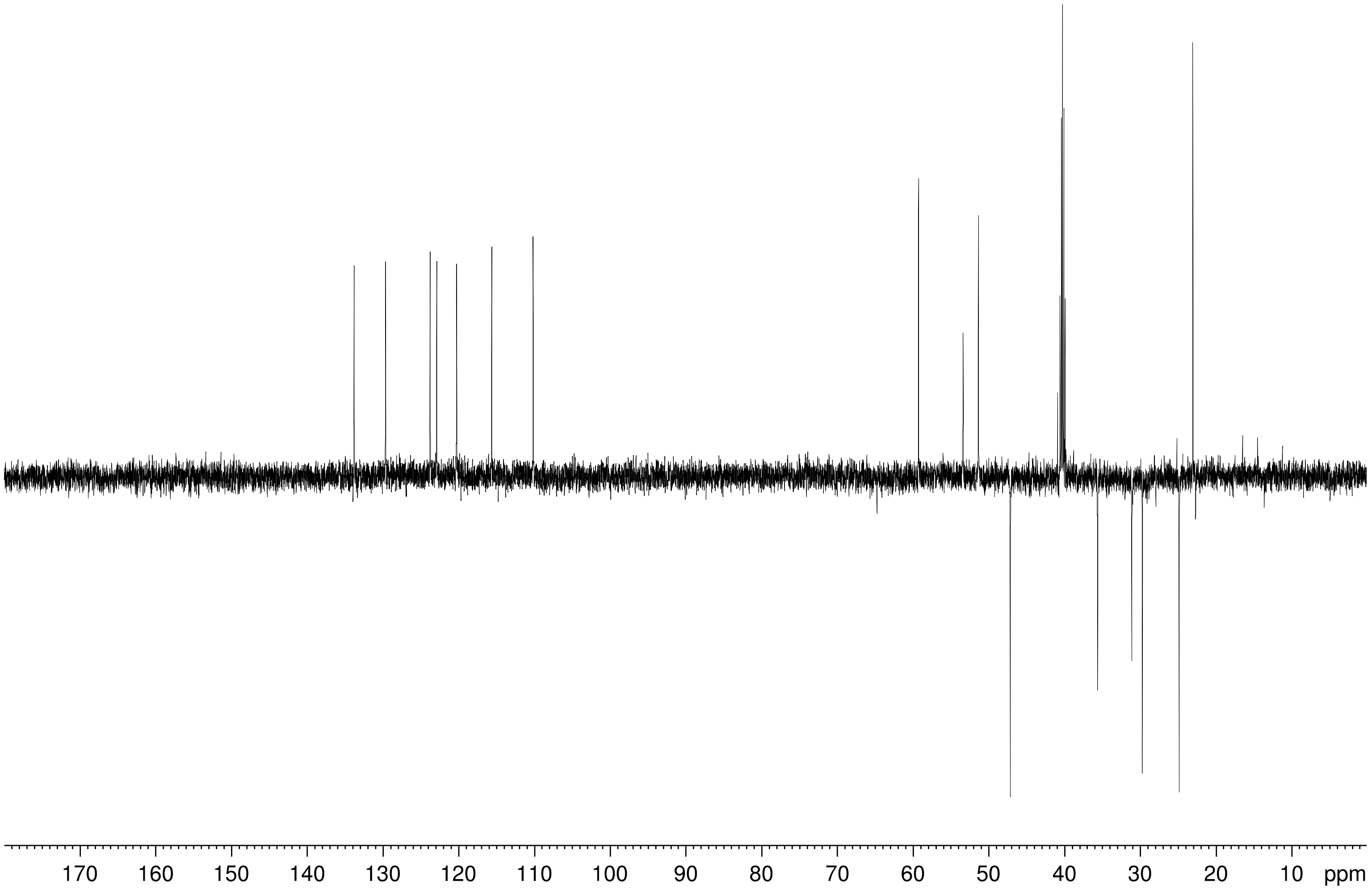

Figure S4. ^{1}H-^{1}H COSY spectrum of **1** (500 MHz, DMSO-d_6).

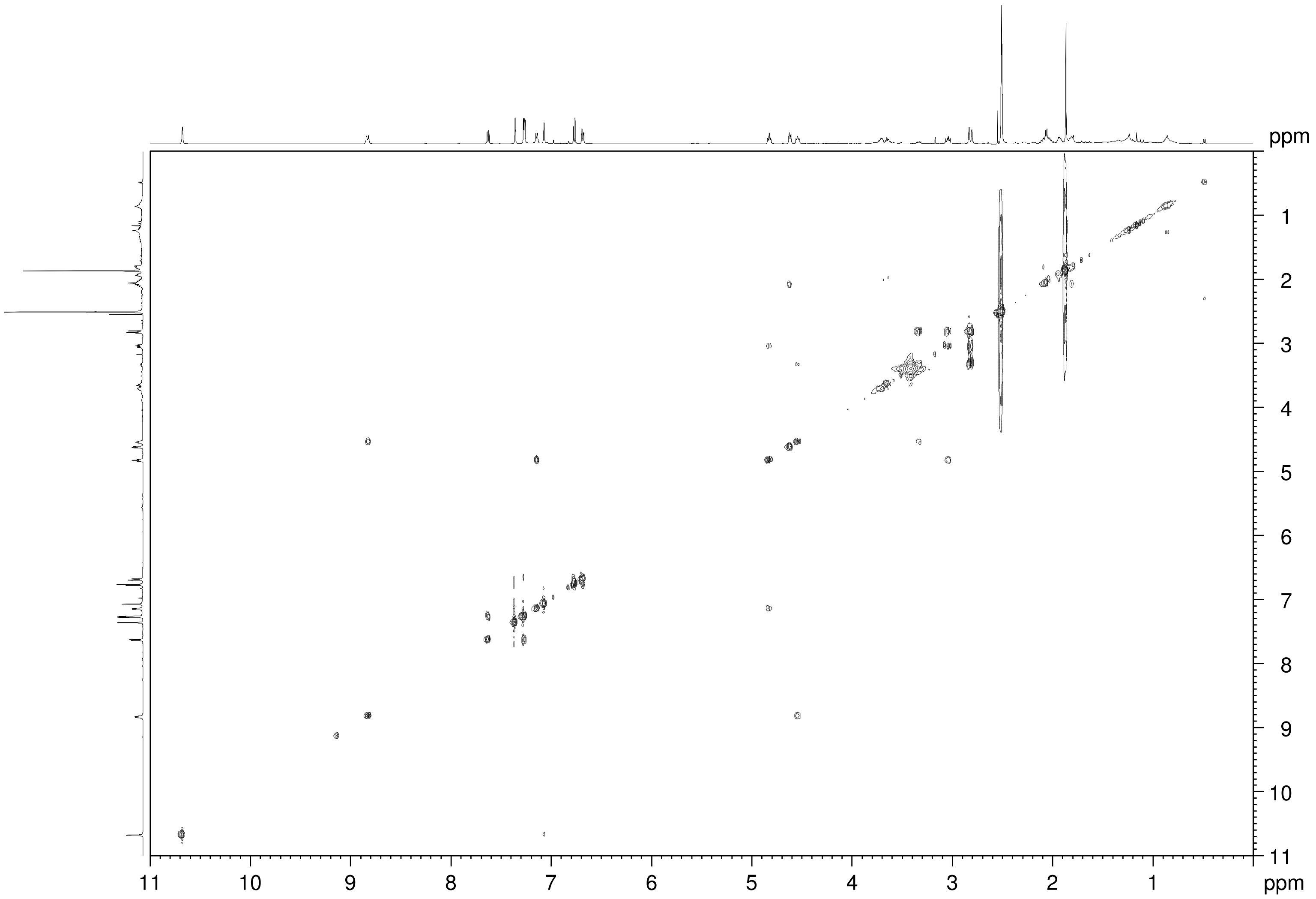

Figure S5. HSQC spectrum of **1** (500 MHz, DMSO-d_6).

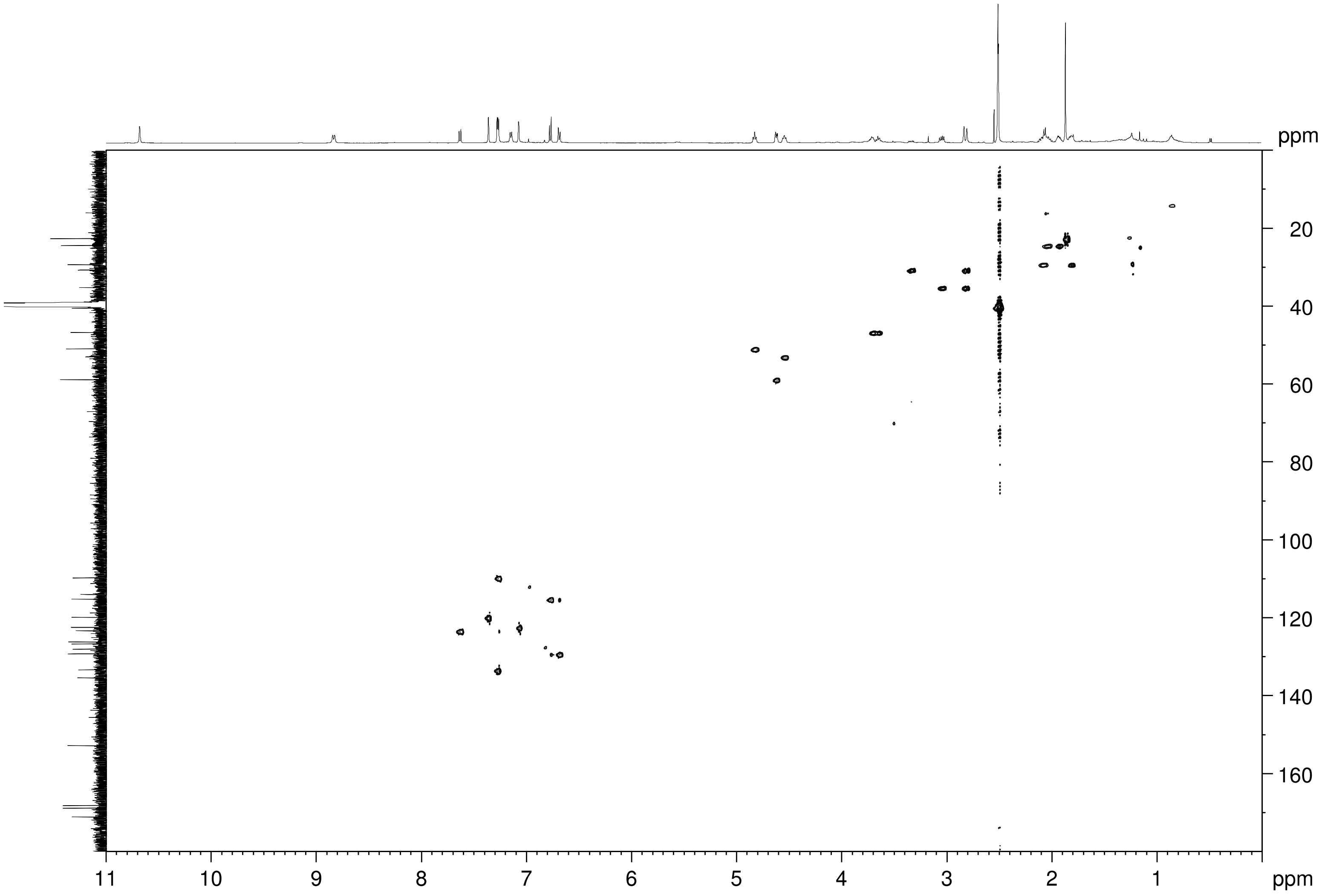

Figure S6. HMBC spectrum of **1** (500 MHz, DMSO-d_6).

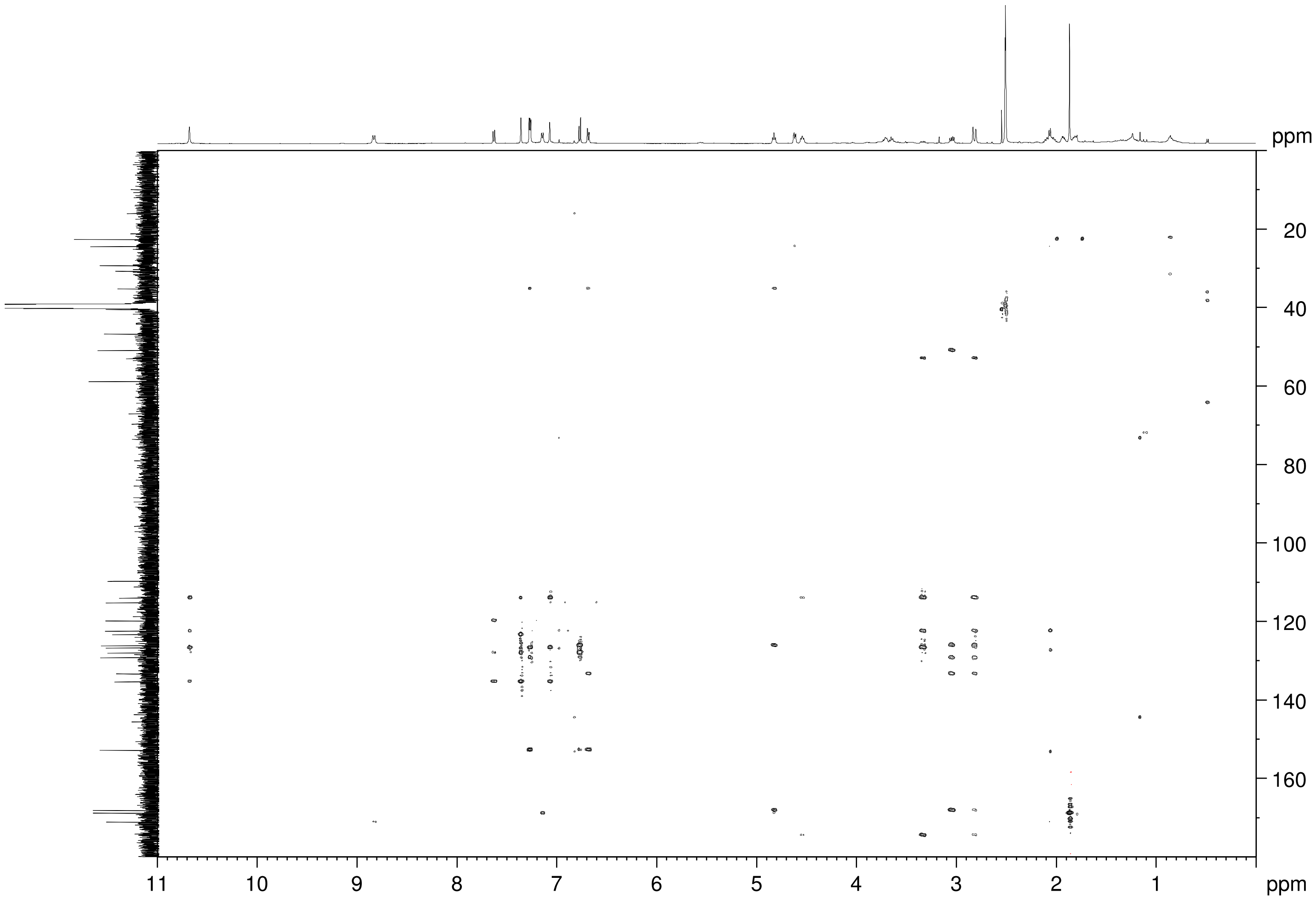

Figure S7. ROESY spectrum of **1** (500 MHz, DMSO-d_6).

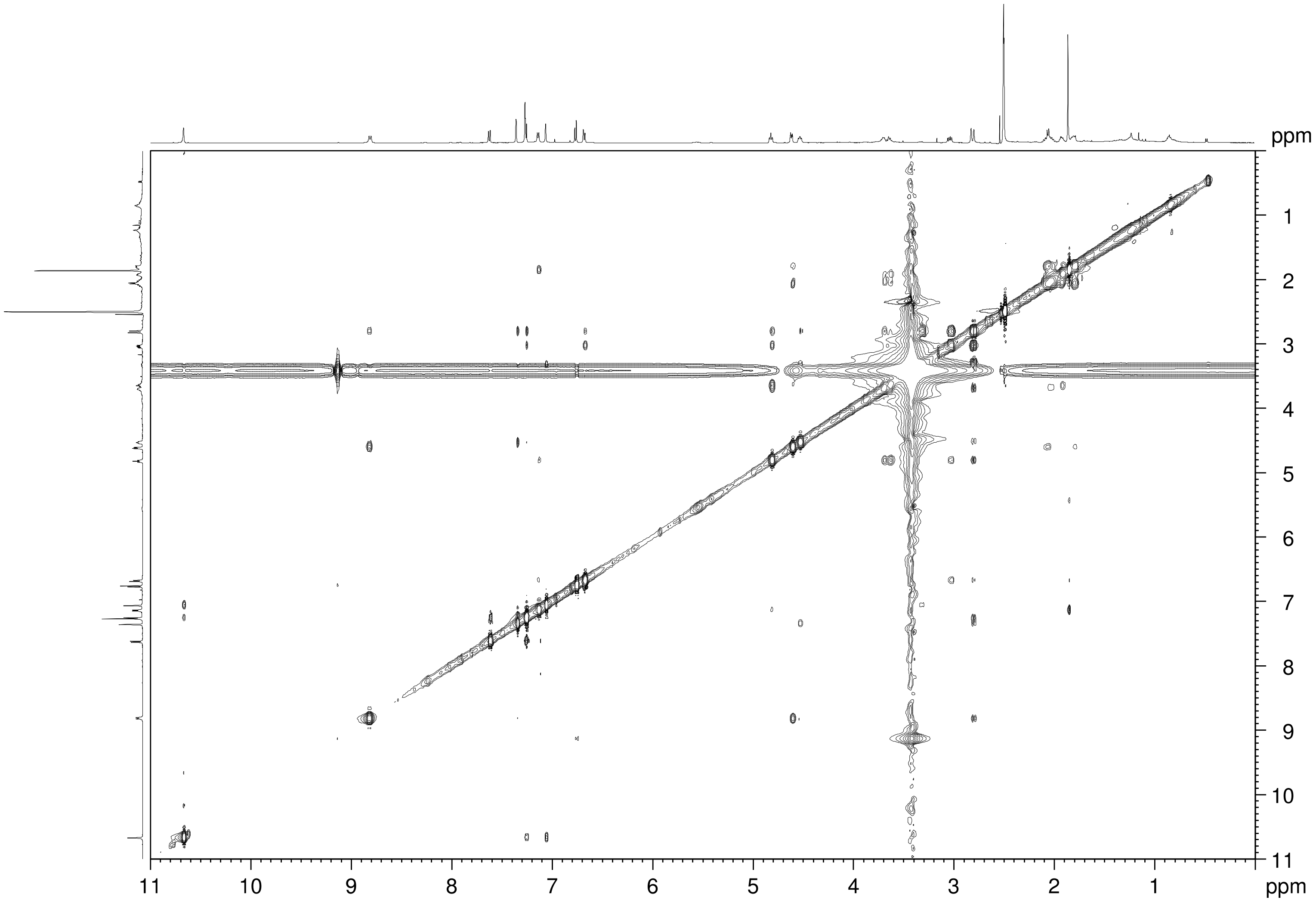

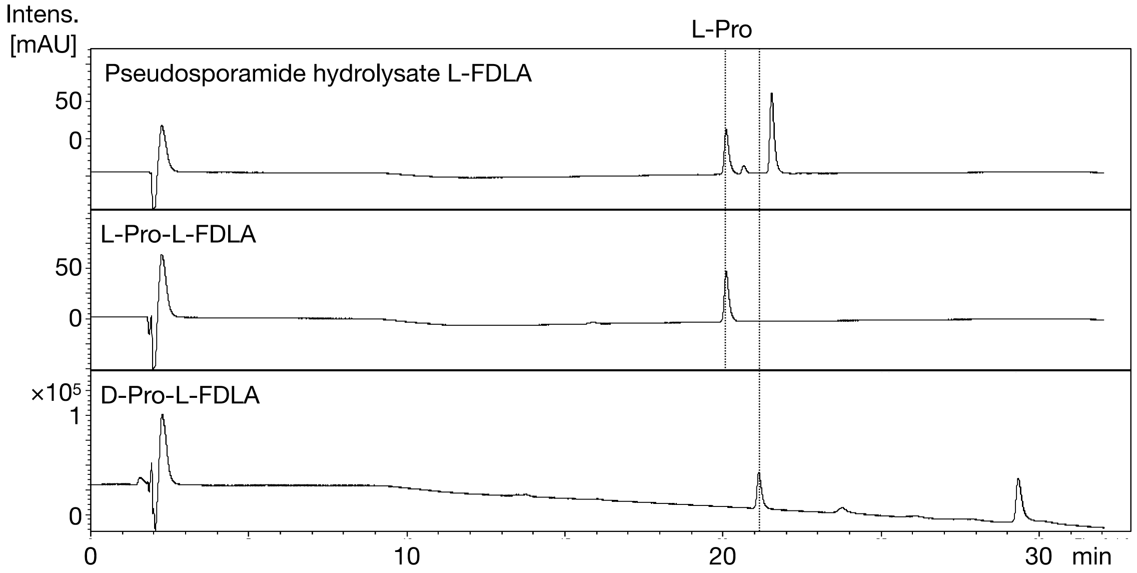

Figure S8. HPLC chromatogram of hydrolysate from **1** and comparison to standard amino acids, derivatized with Marfey's reagent (FDLA).

Column: Cosmosil AR-II (Nacalai Tesque, 4.6×250 mm)
Solvent: MeCN-0.1% HCO_2H
Gradient MeCN (%): 0-30 min: 15-80%, 30-35 min: 80%
Flow rate: 1.0 mL/min
Absorbance: 340 nm

Figure S9. ^{1}H NMR spectrum of **5a** (500 MHz, DMSO-d_6).

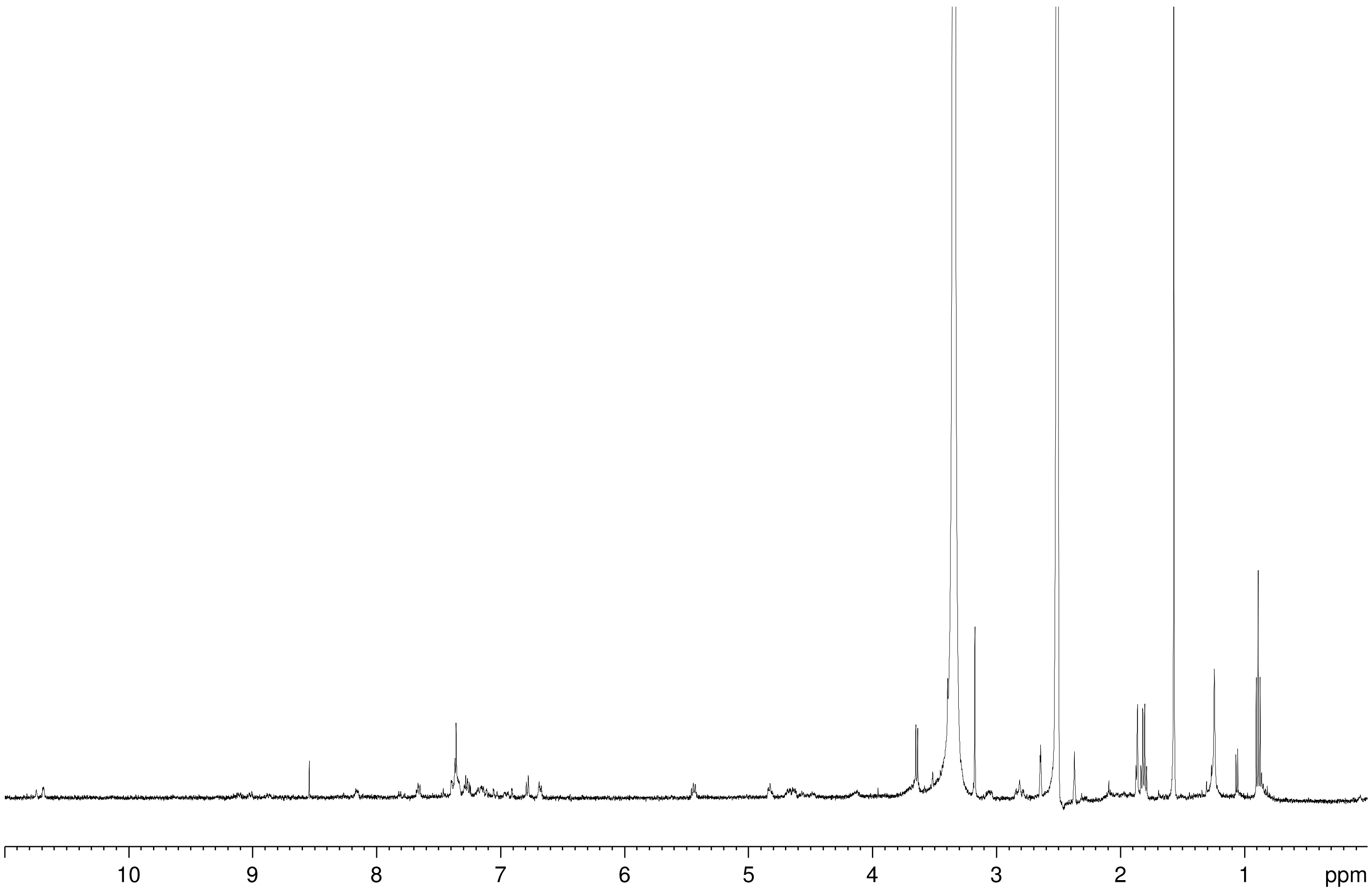

Figure S10. ^{1}H-^{1}H COSY spectrum of **5a** (500 MHz, DMSO-d_6).

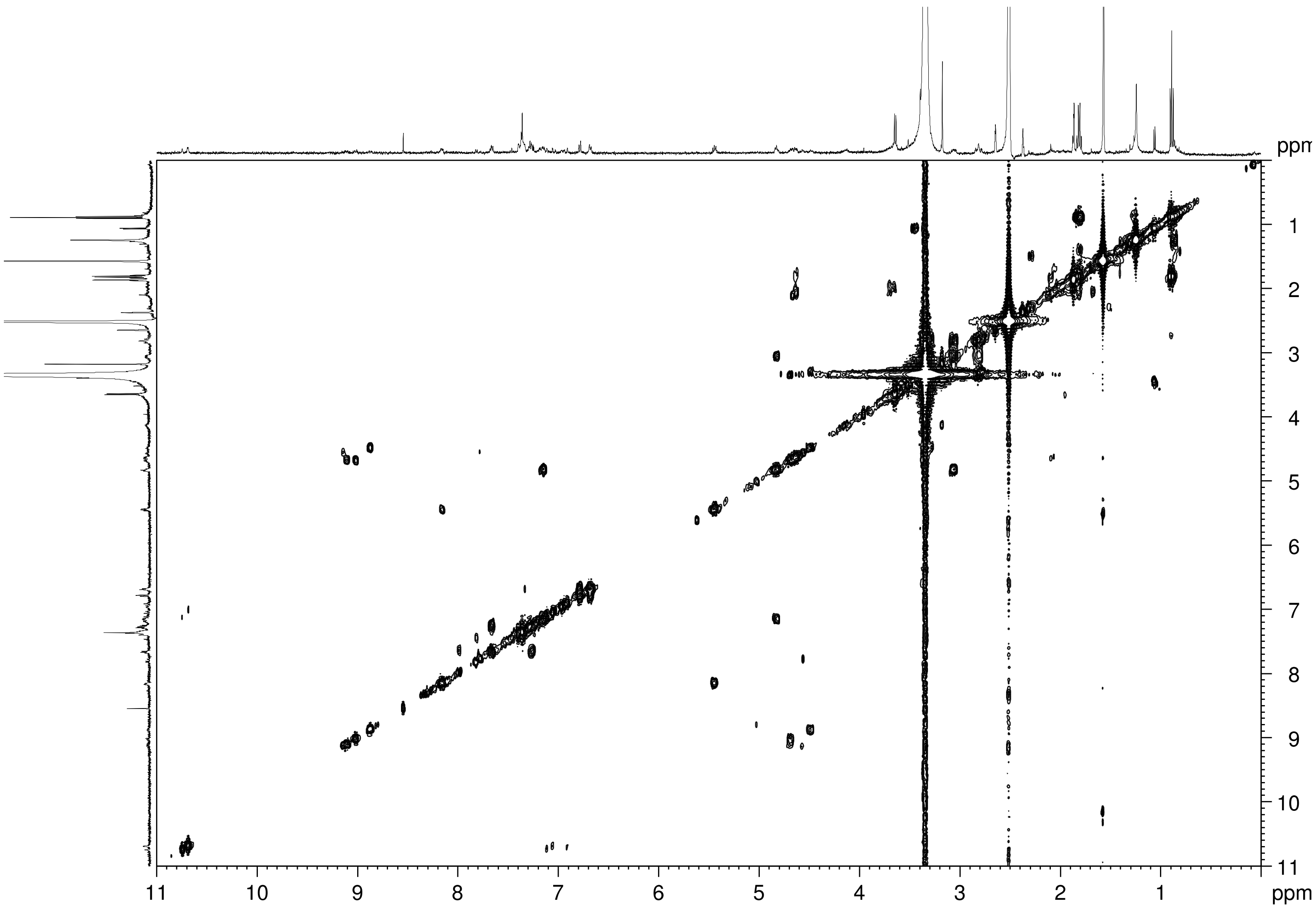

Figure S11. ^{1}H NMR spectrum of **5b** (500 MHz, DMSO-d_6).

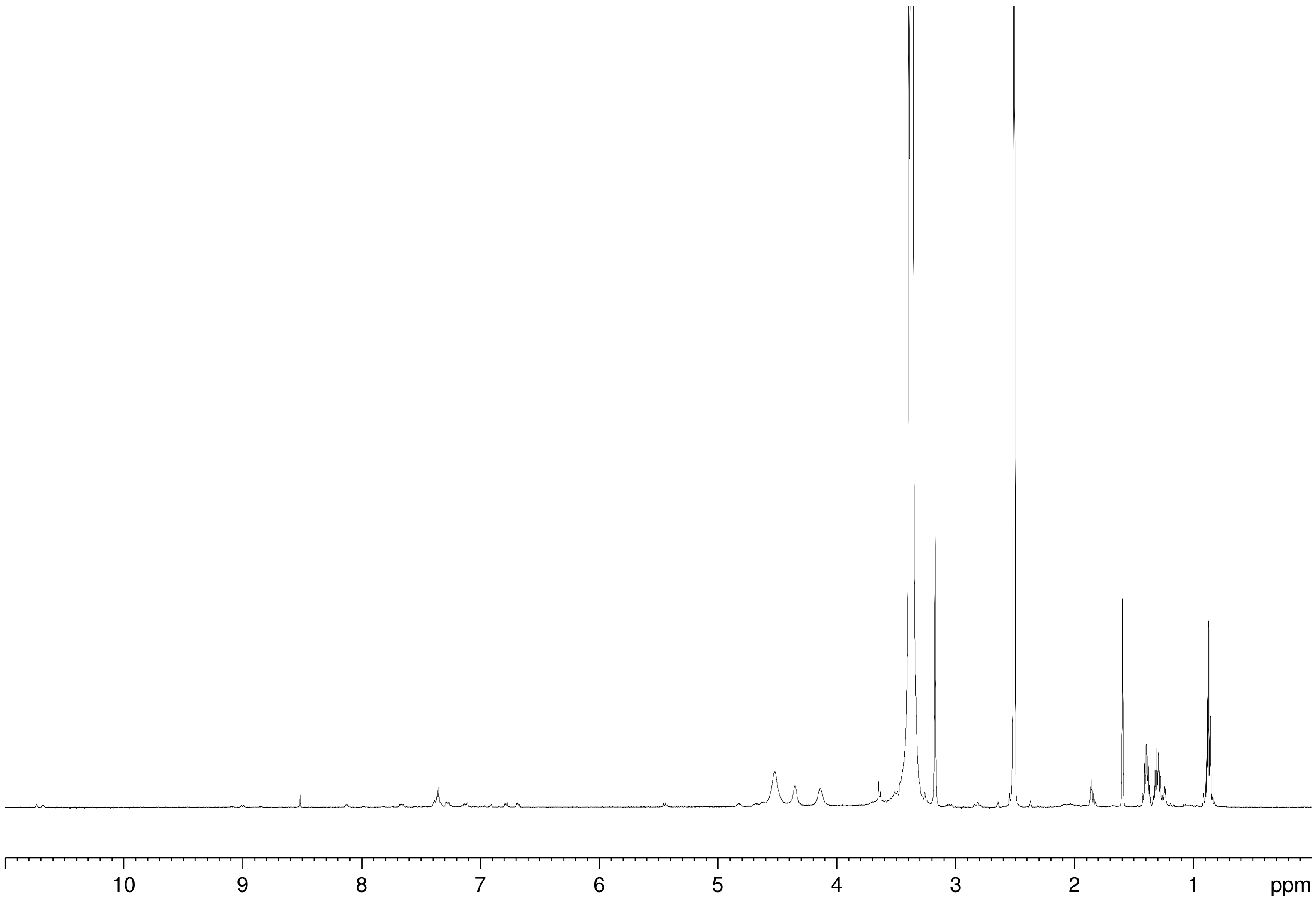

Figure S12. ^{1}H-^{1}H COSY spectrum of **5b** (500 MHz, DMSO-d_6).

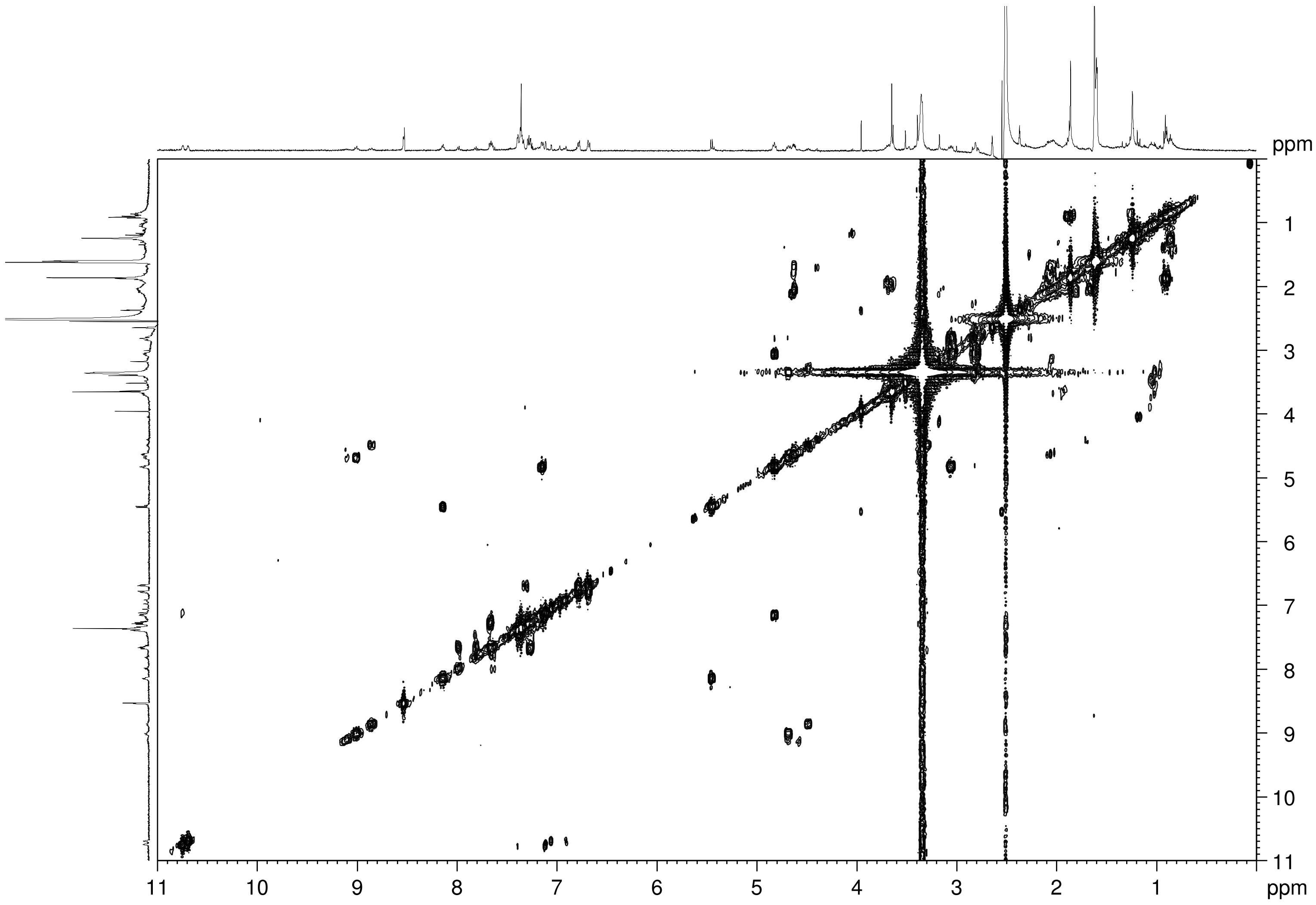

Figure S13. ^{1}H NMR spectrum of pseudosporamicin A (**2**) (500 MHz, $CDCl_3$).

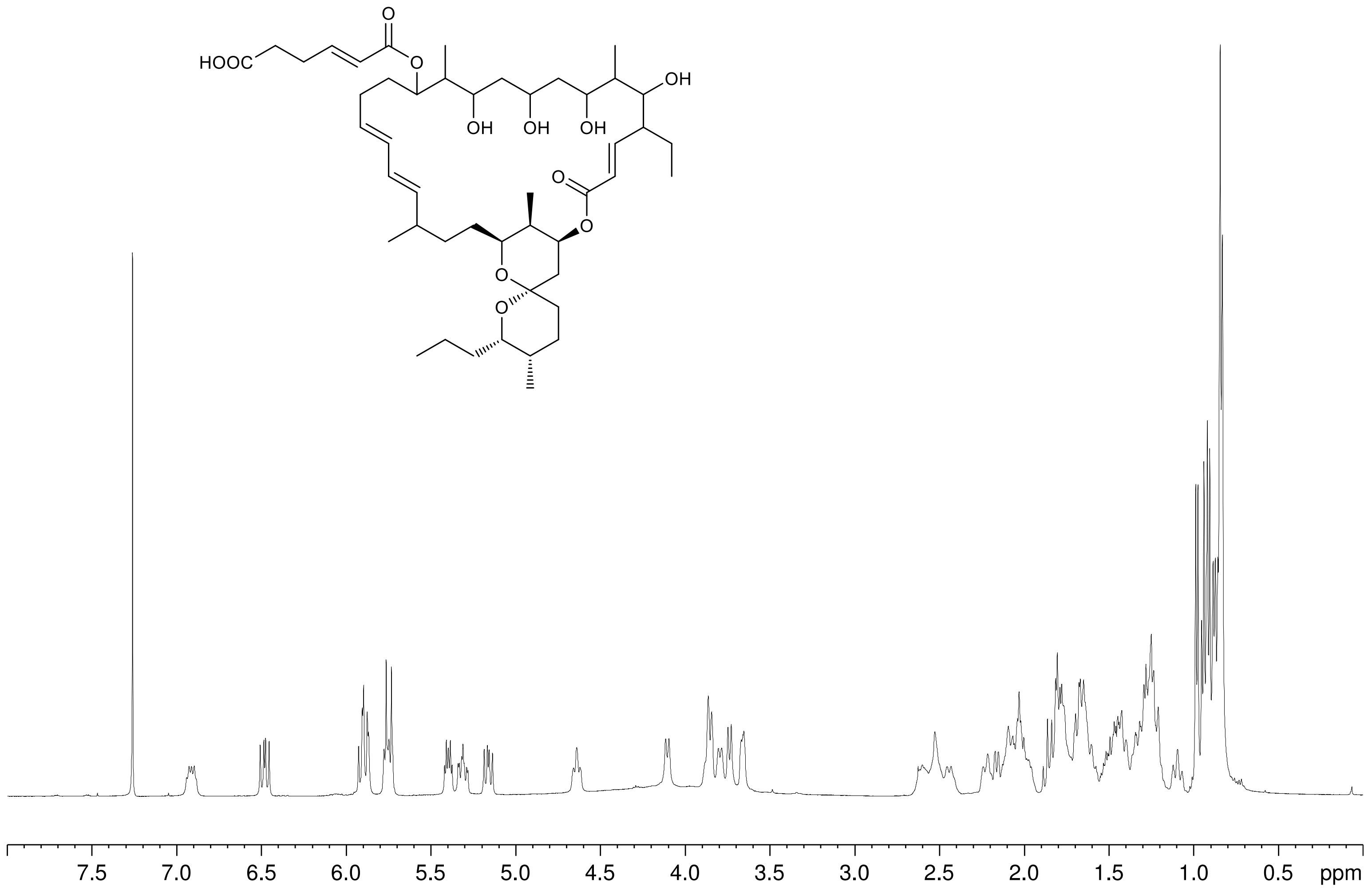

Figure S14. ^{13}C NMR spectrum of **2** (125 MHz, $CDCl_3$).

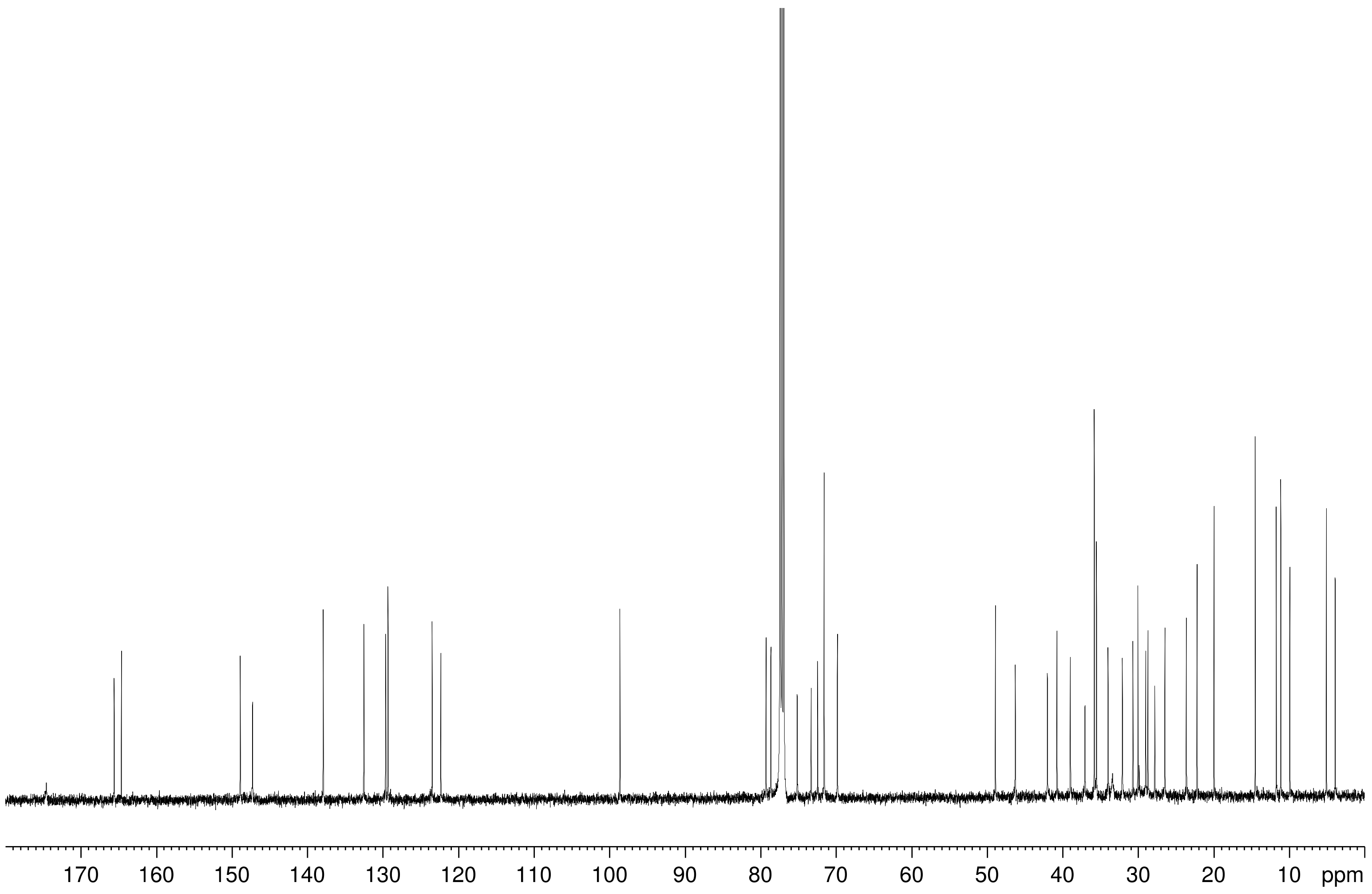

Figure S15. ^{1}H-^{1}H COSY spectrum of **2** (500 MHz, $CDCl_3$).

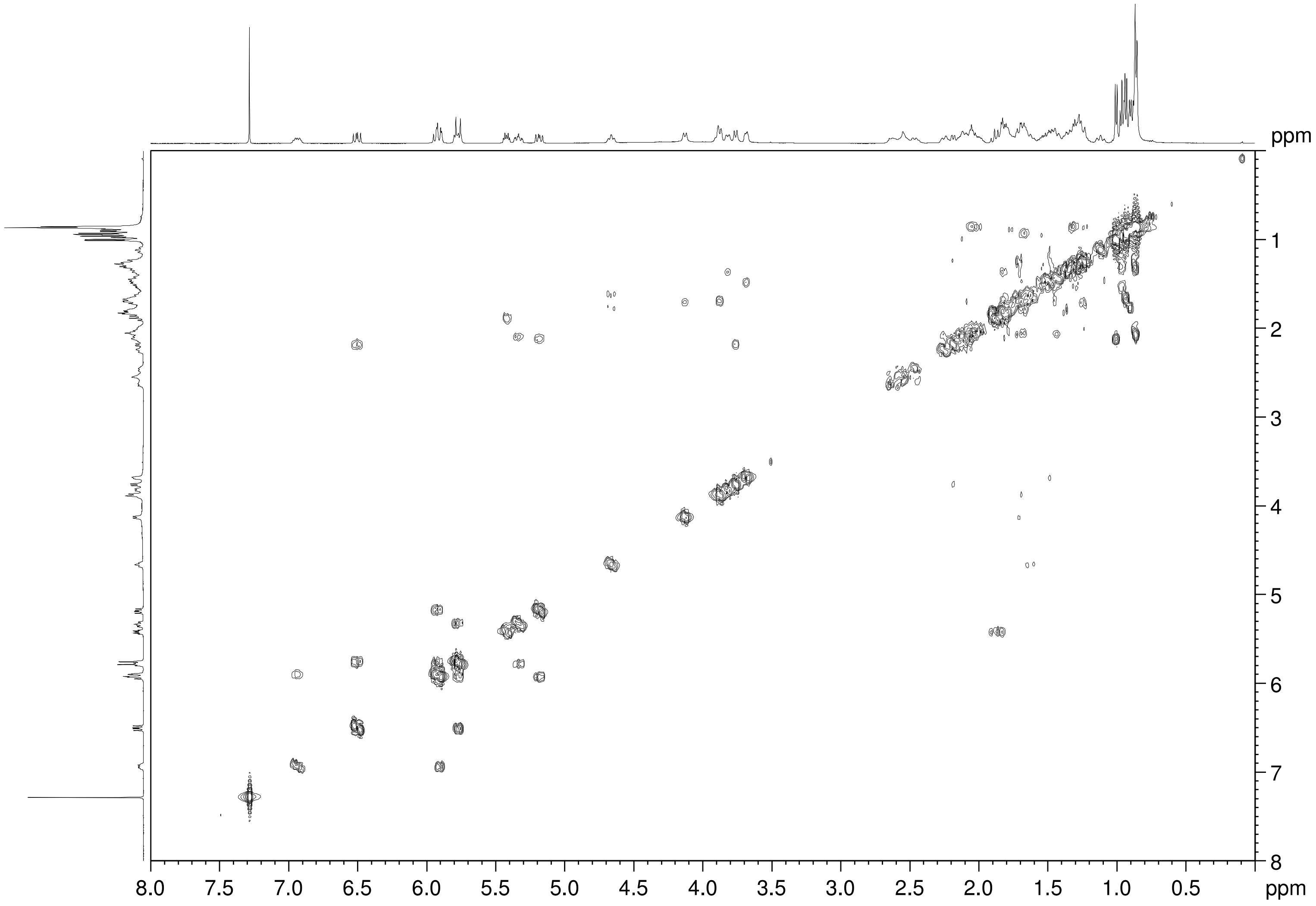

Figure S16. HSQC spectrum of **2** (500 MHz, $CDCl_3$).

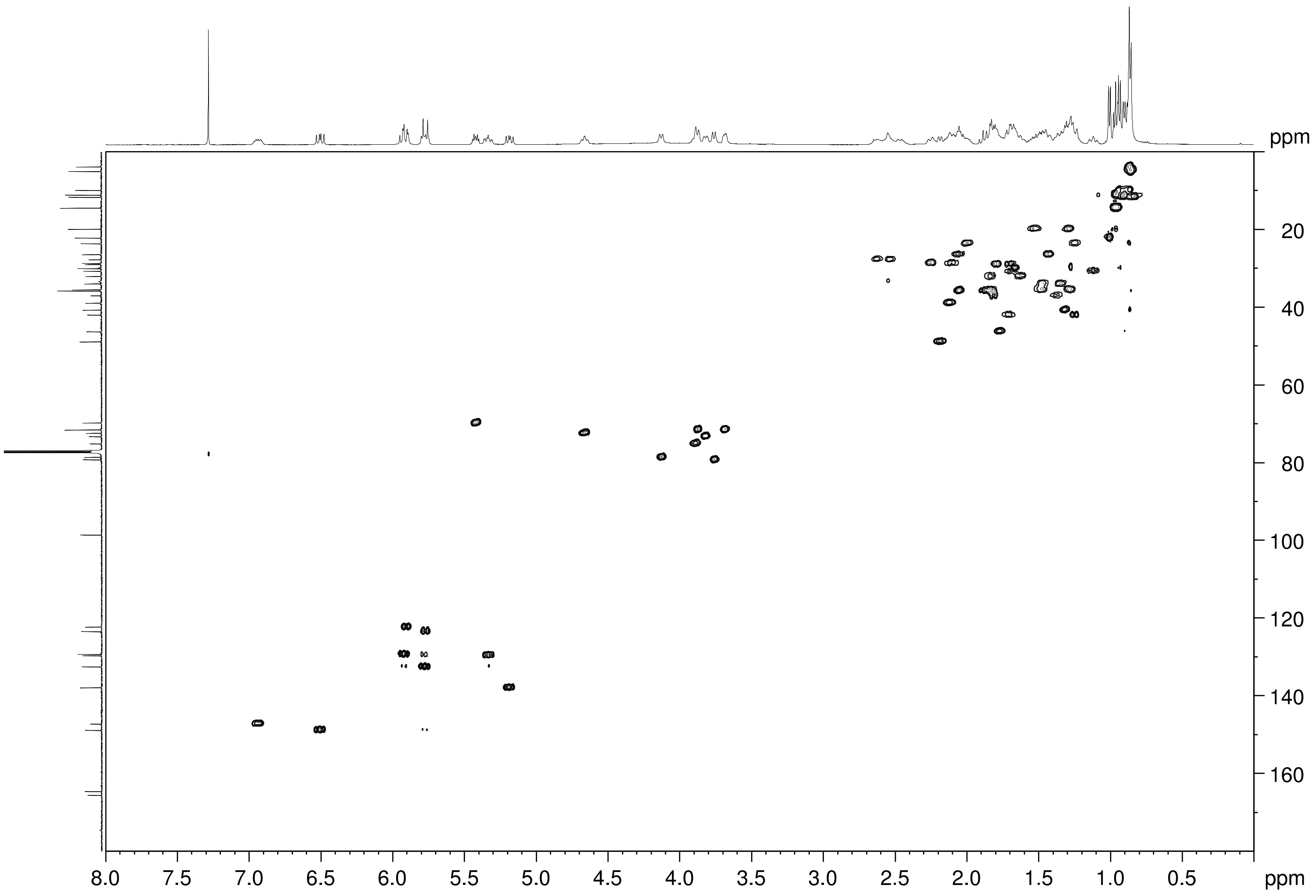

Figure S17. HMBC spectrum of **2** (500 MHz, $CDCl_3$).

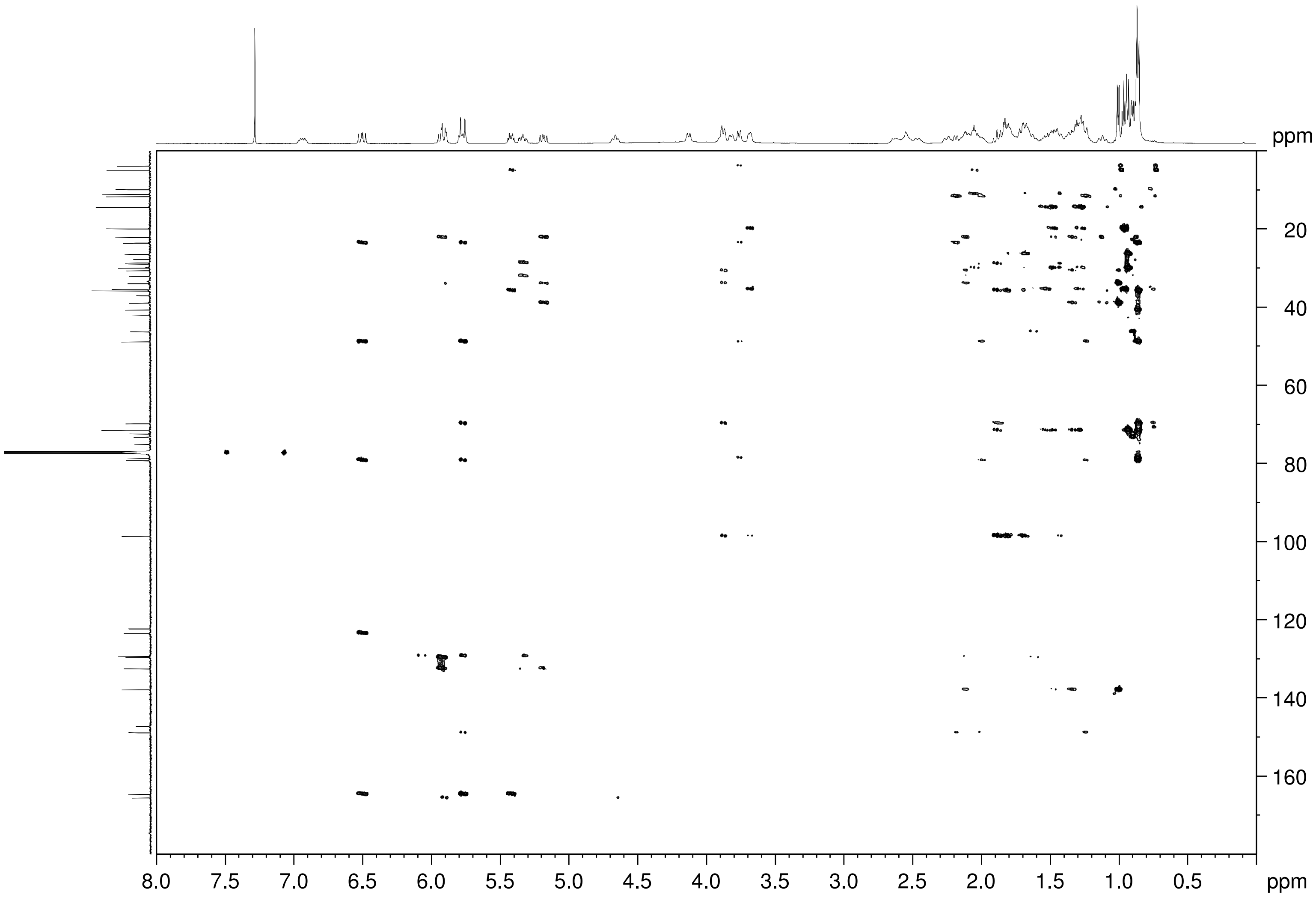

Figure S18. NOESY spectrum of **2** (500 MHz, $CDCl_3$).

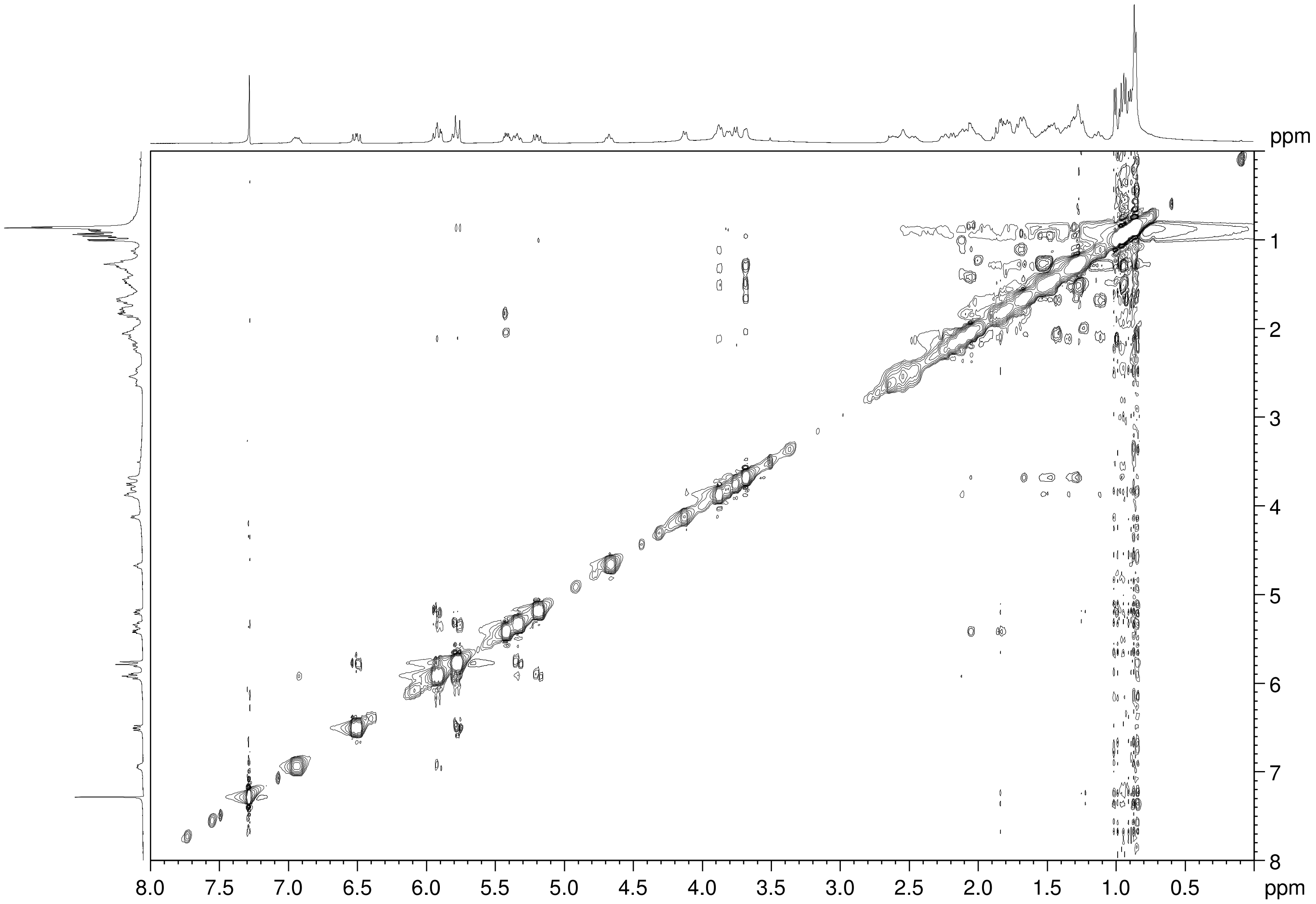

Figure S19. ^{1}H NMR spectrum of pseudosporamicin B (**3**) (500 MHz, $CDCl_3$).

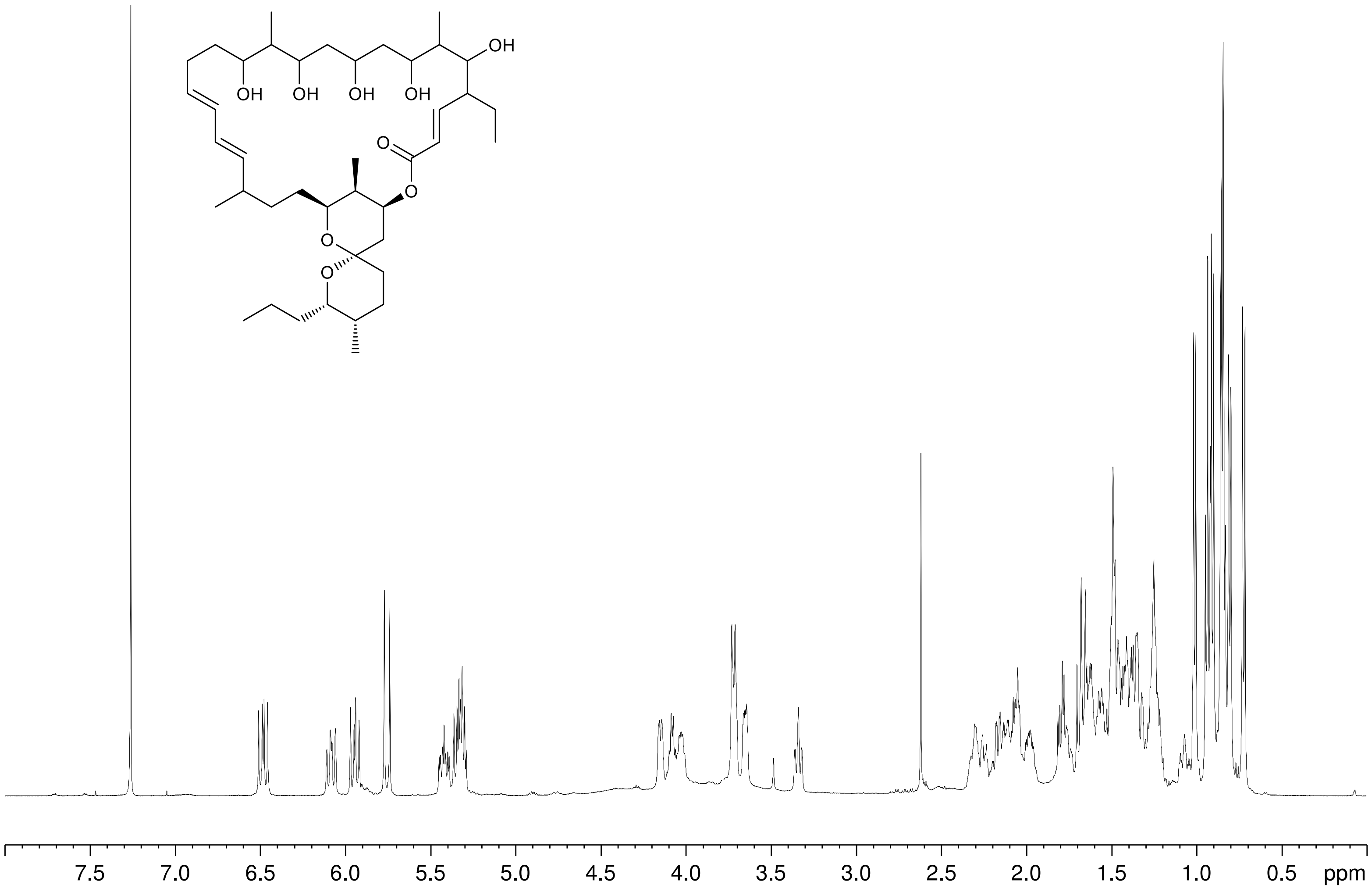

Figure S20. ^{13}C NMR spectrum of **3** (125 MHz, $CDCl_3$).

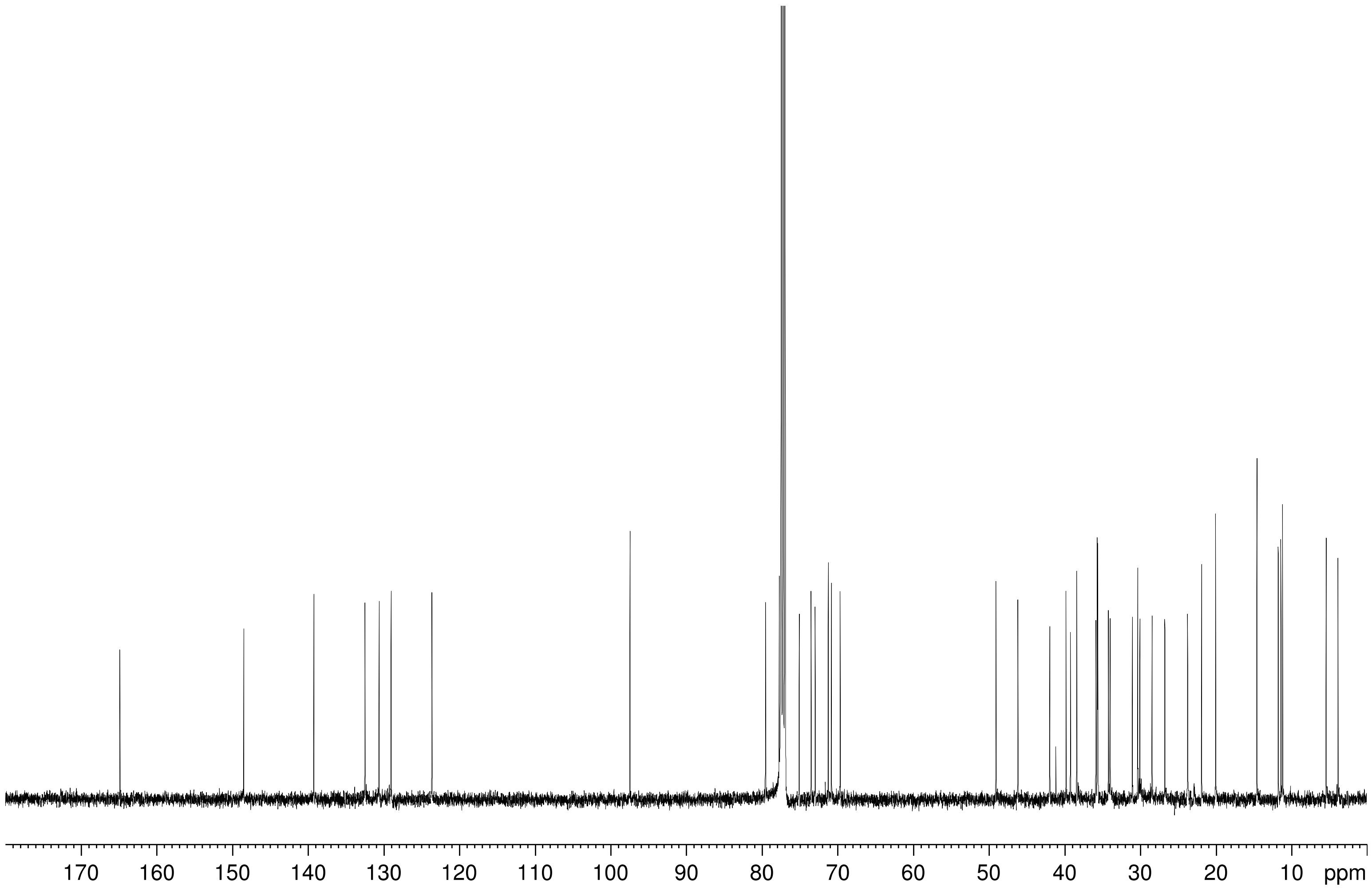

Figure S21. ^{1}H-^{1}H COSY spectrum of **3** (500 MHz, $CDCl_3$).

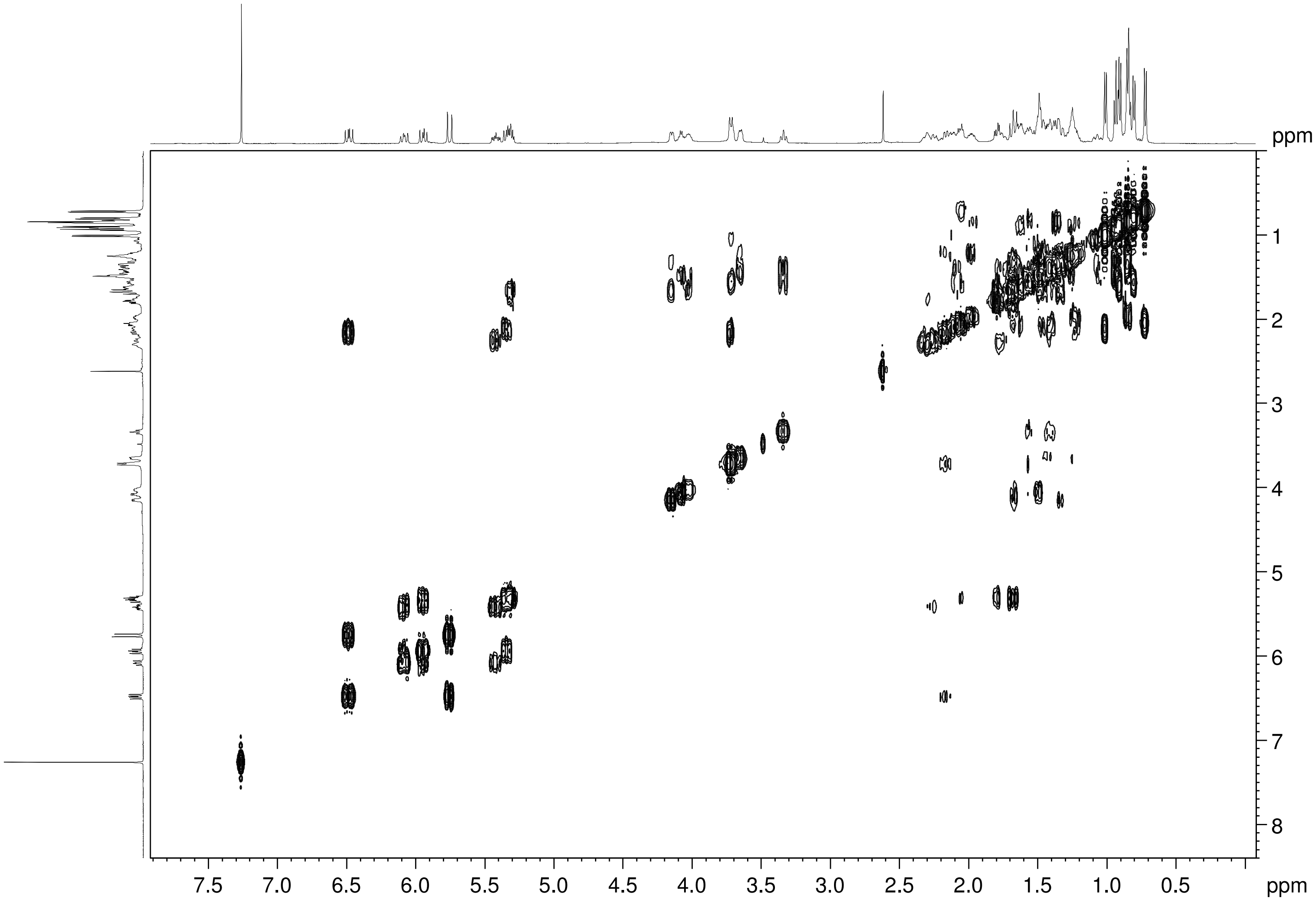

Figure S22. HSQC spectrum of **3** (500 MHz, $CDCl_3$).

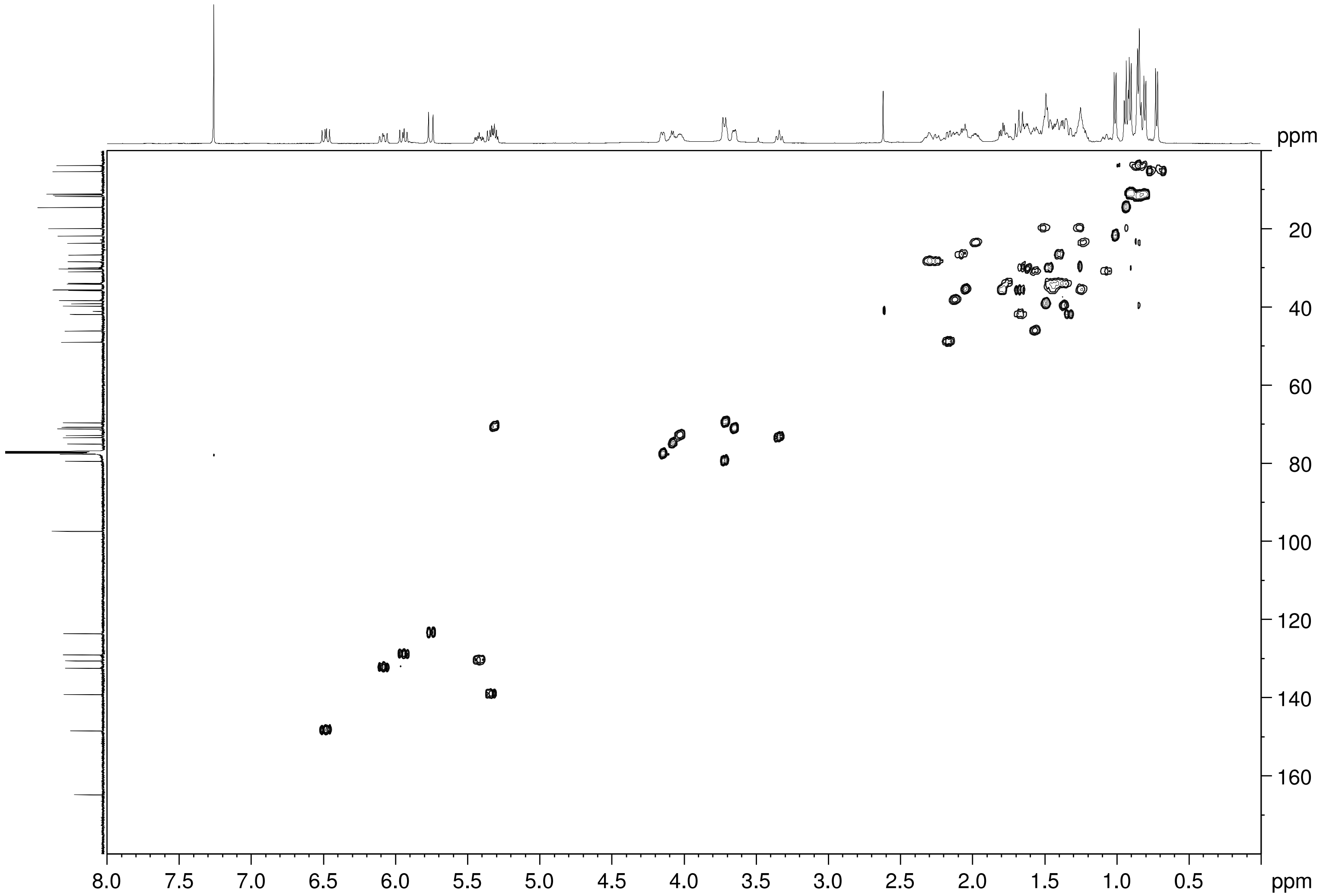

Figure S23. HMBC spectrum of **3** (500 MHz, $CDCl_3$).

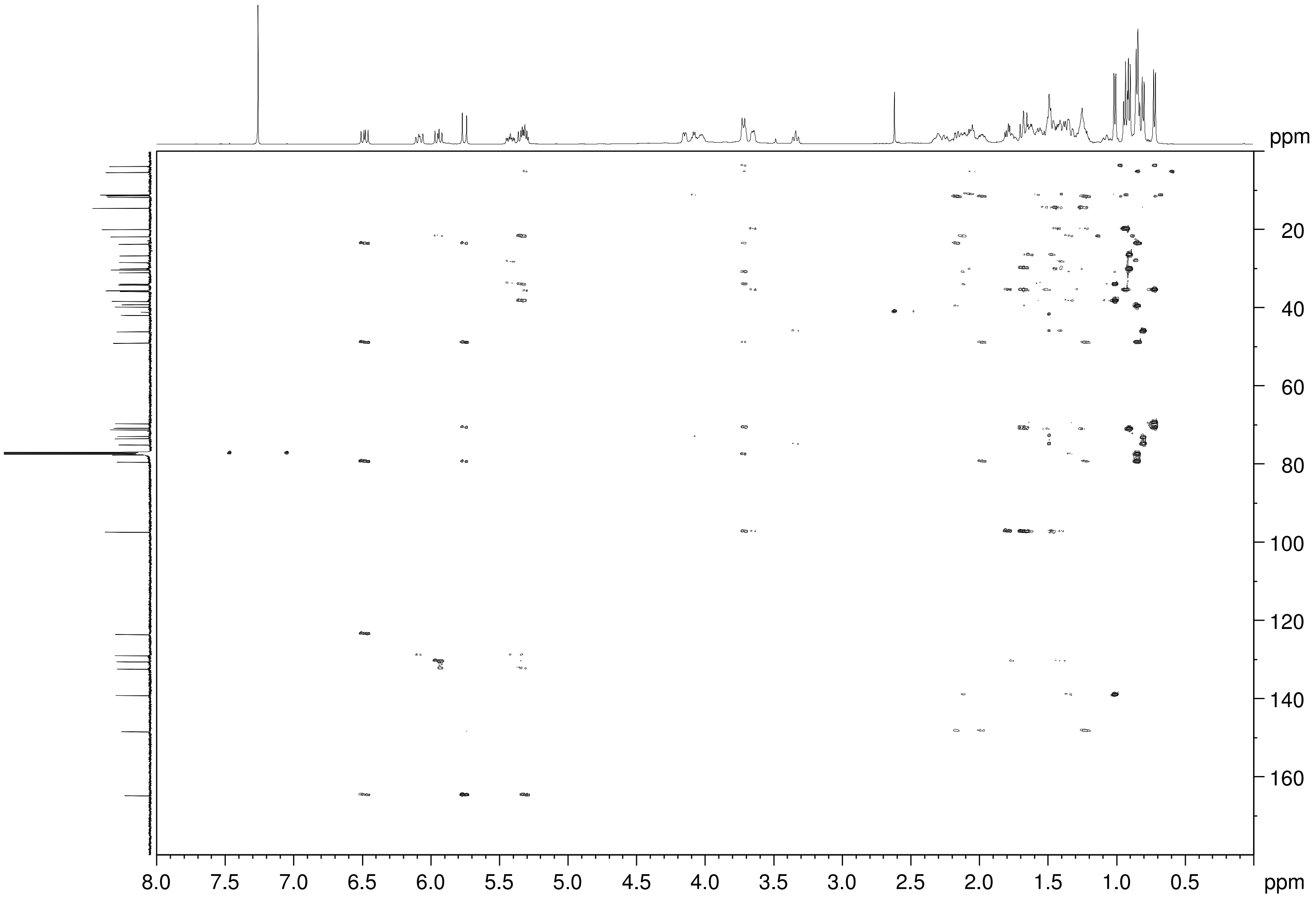

Figure S24. NOESY spectrum of **3** (500 MHz, $CDCl_3$).

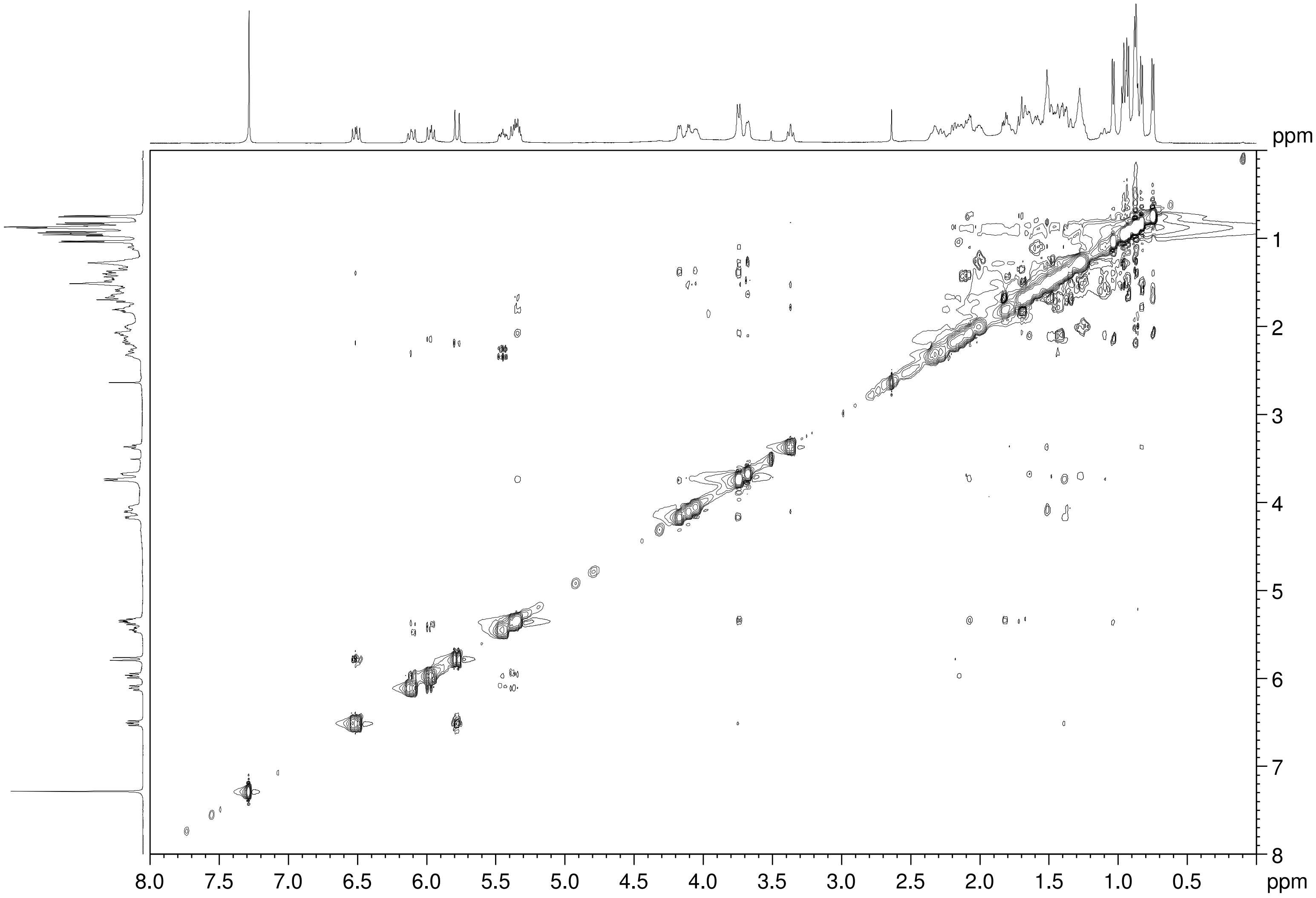

Figure S25. ^{1}H NMR spectrum of pseudosporamicin C (**4**) (500 MHz, $CDCl_3$).

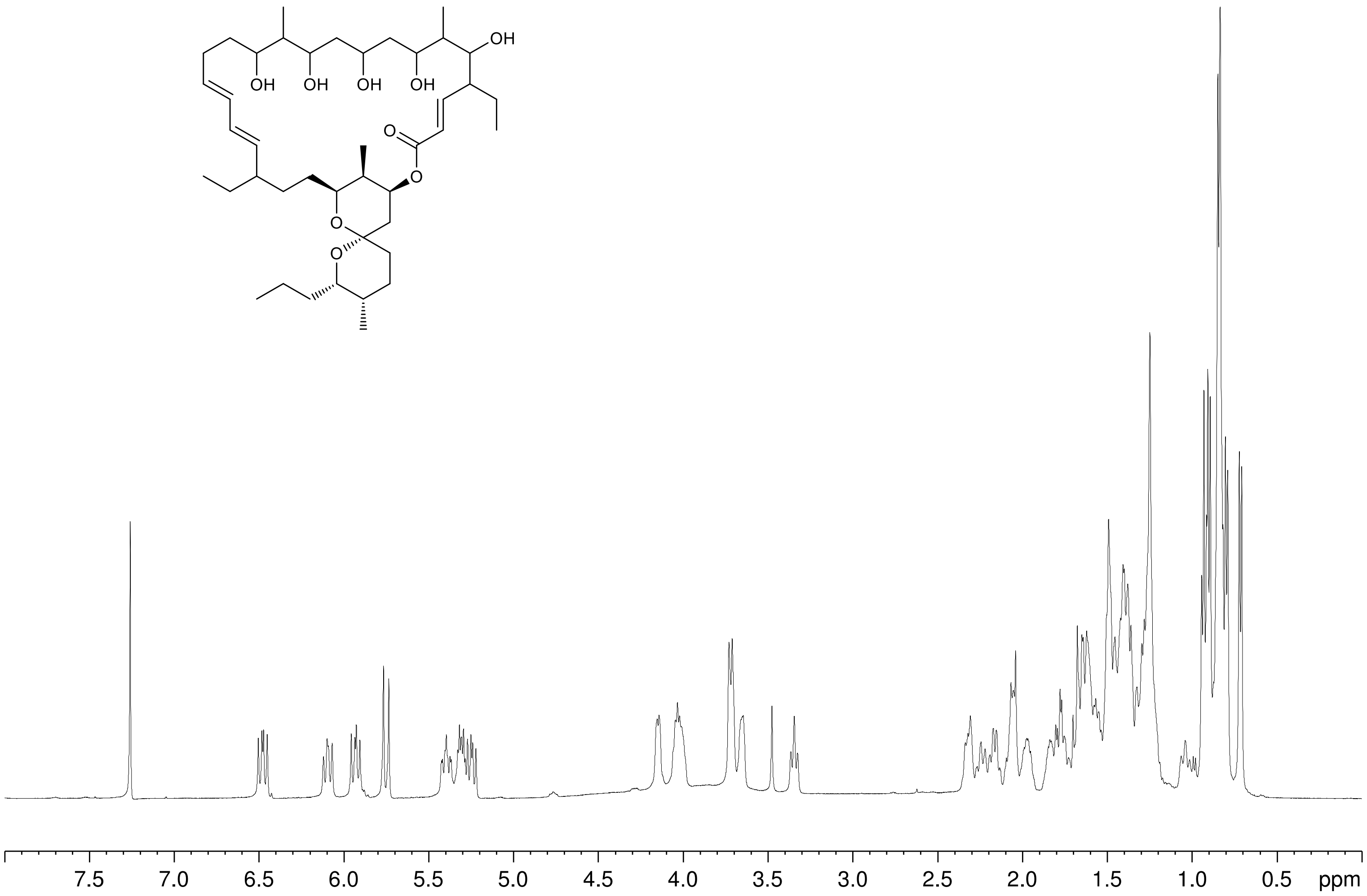

Figure S26. ^{13}C NMR spectrum of **4** (125 MHz, $CDCl_3$).

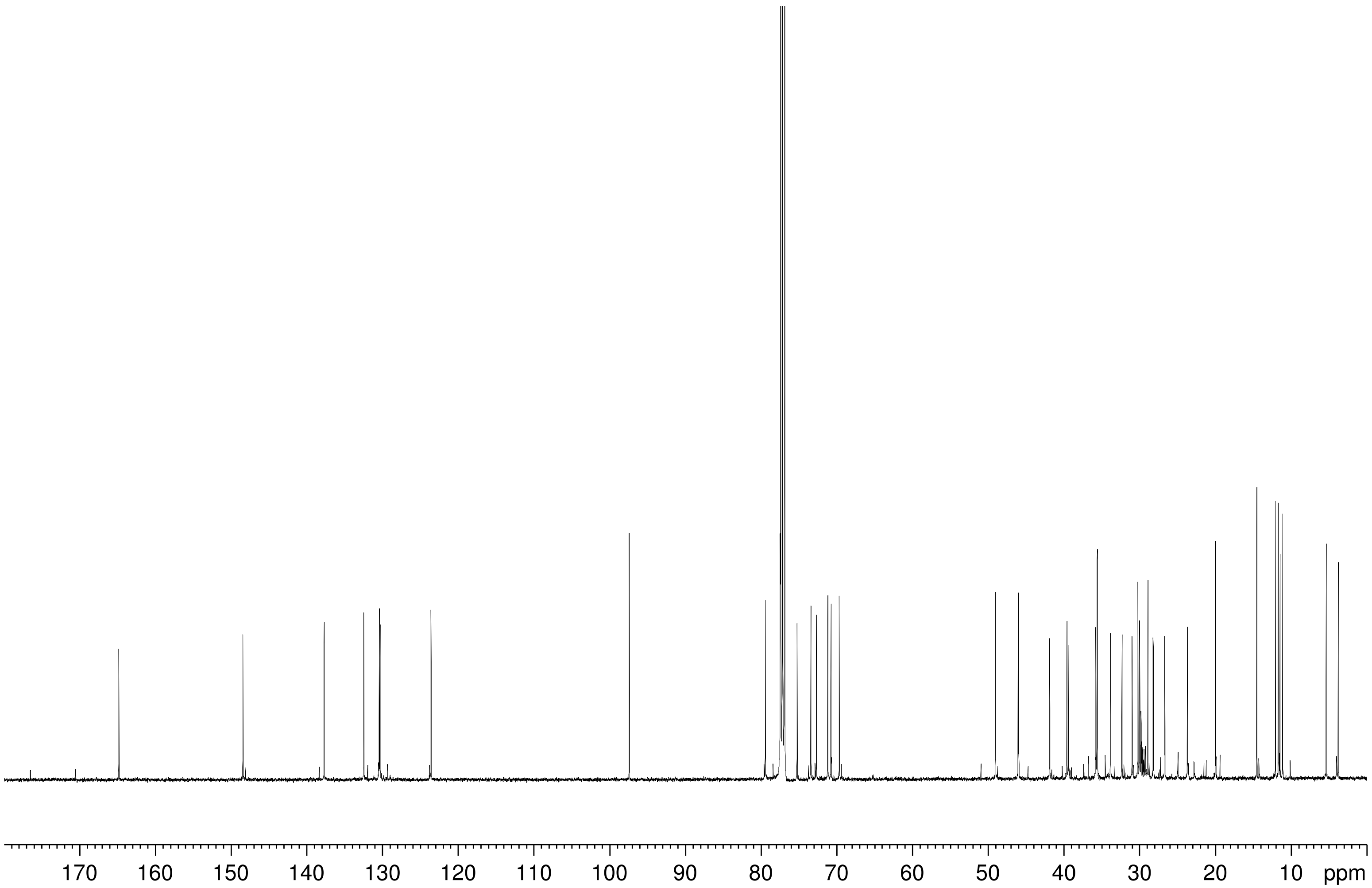

Figure S27. ^{1}H-^{1}H COSY spectrum of **4** (500 MHz, $CDCl_3$).

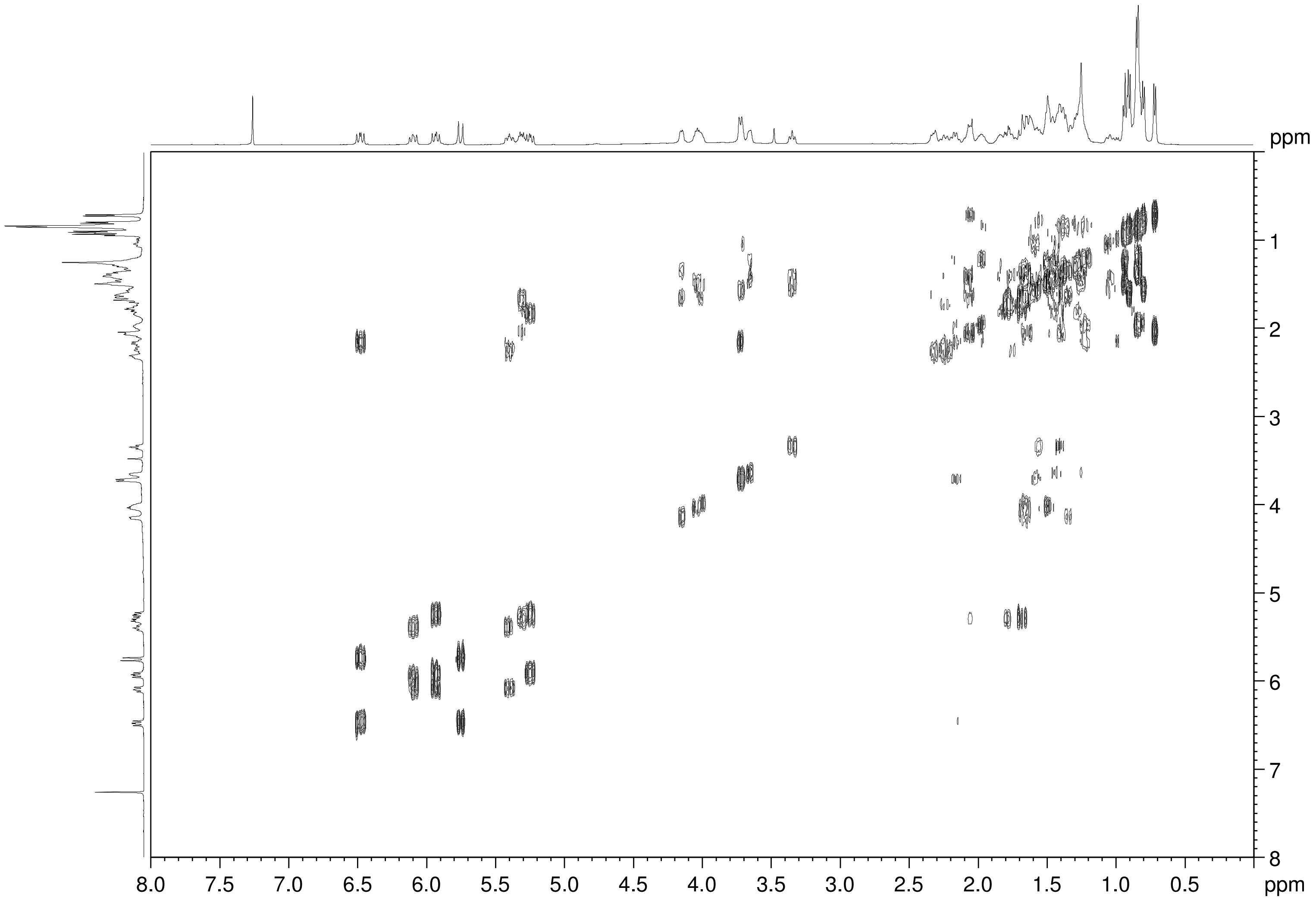

Figure S28. HSQC spectrum of **4** (500 MHz, $CDCl_3$).

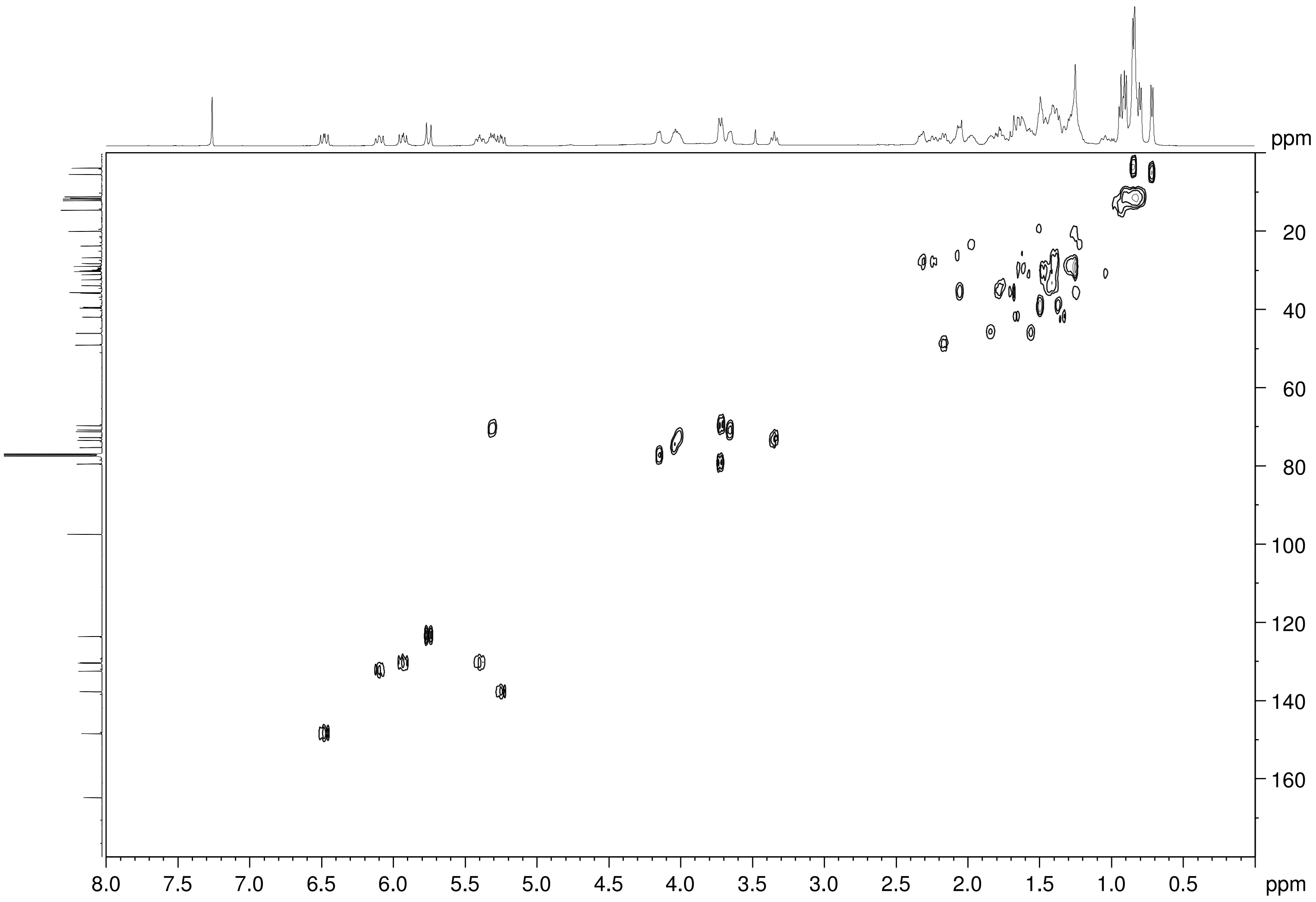

Figure S29. HMBC spectrum of **4** (500 MHz, $CDCl_3$).

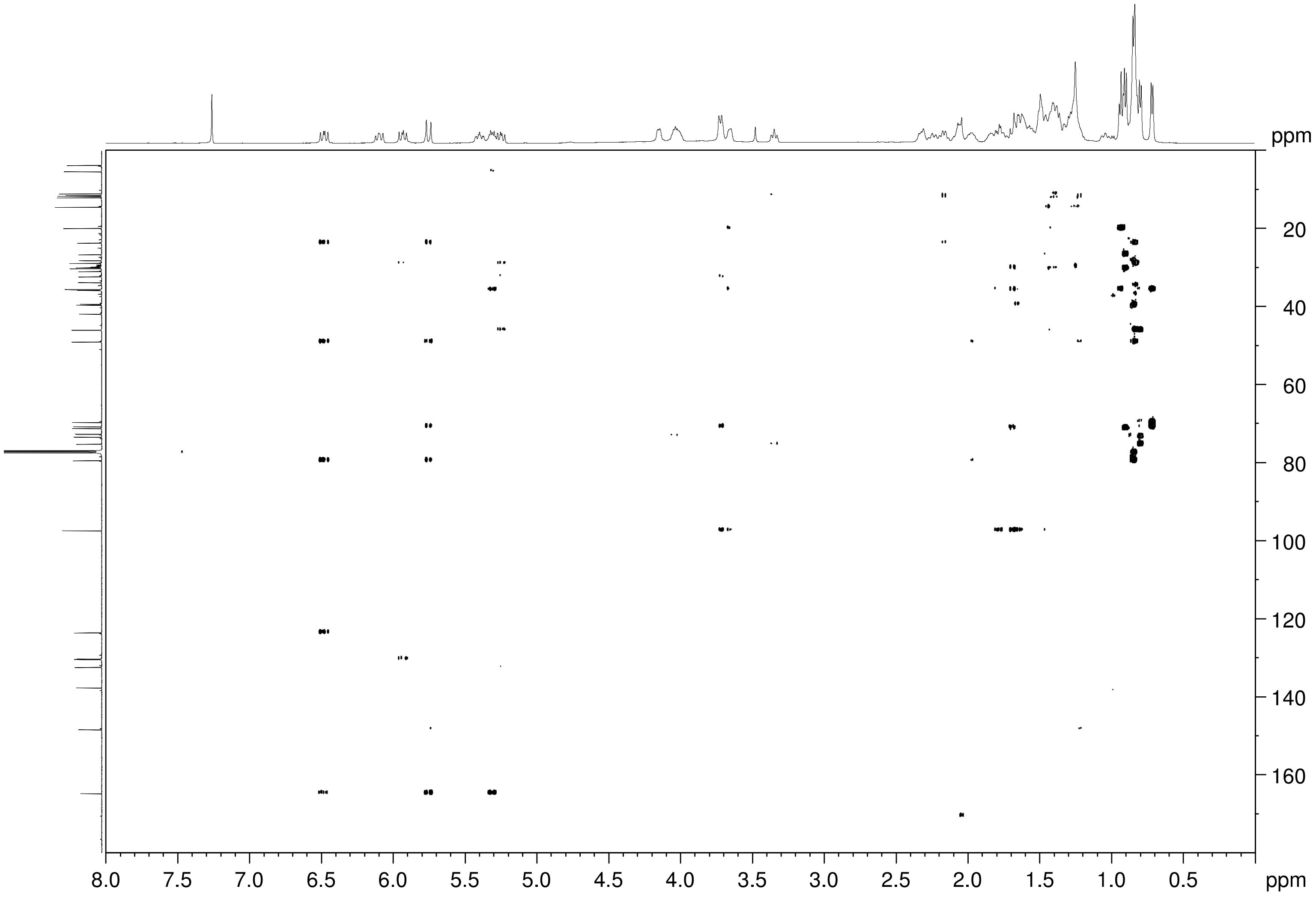

Figure S30. NOESY spectrum of **4** (500 MHz, $CDCl_3$).

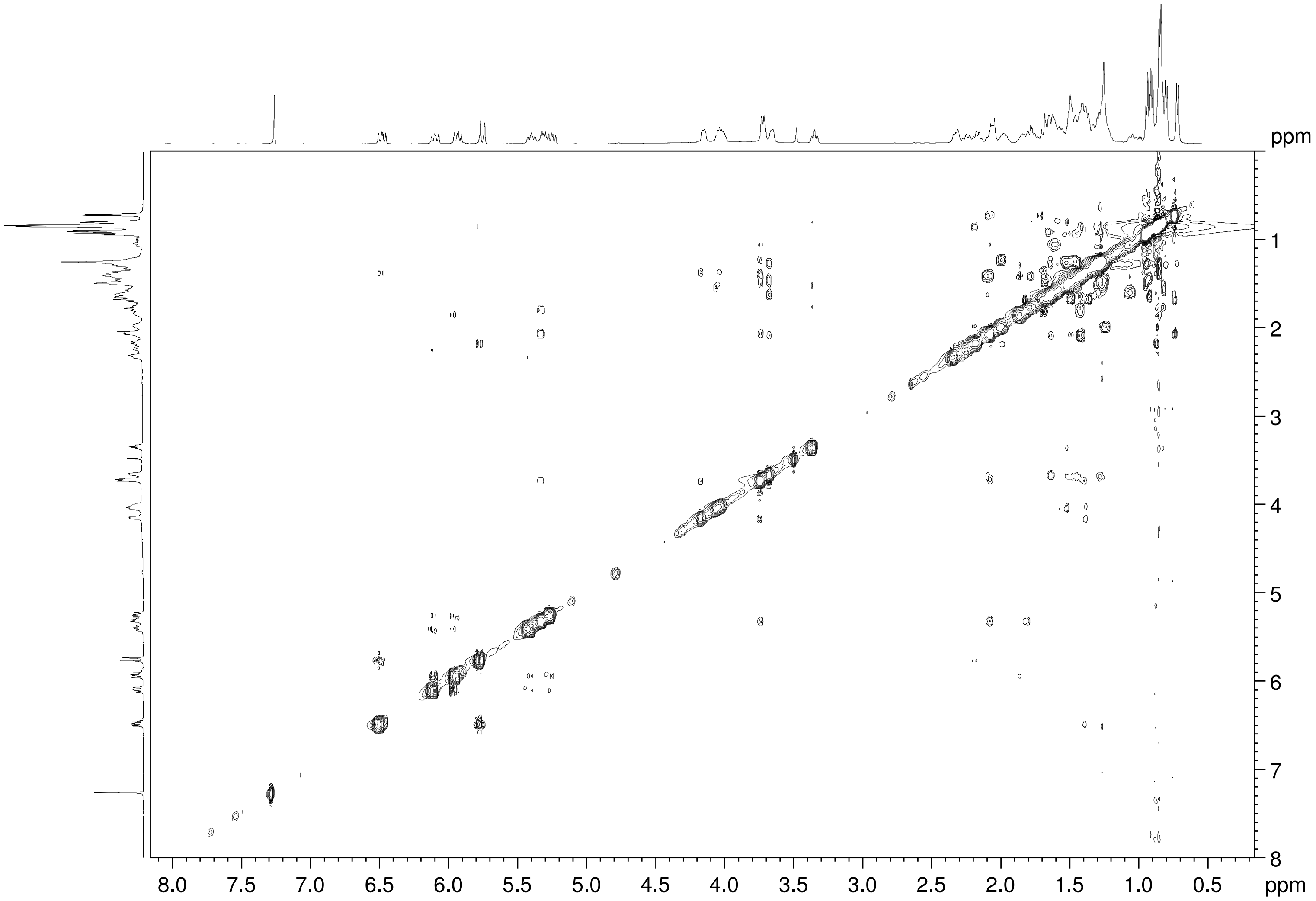

Figure S31. COSY, key HMBC and NOESY correlations of **3** and **4**.

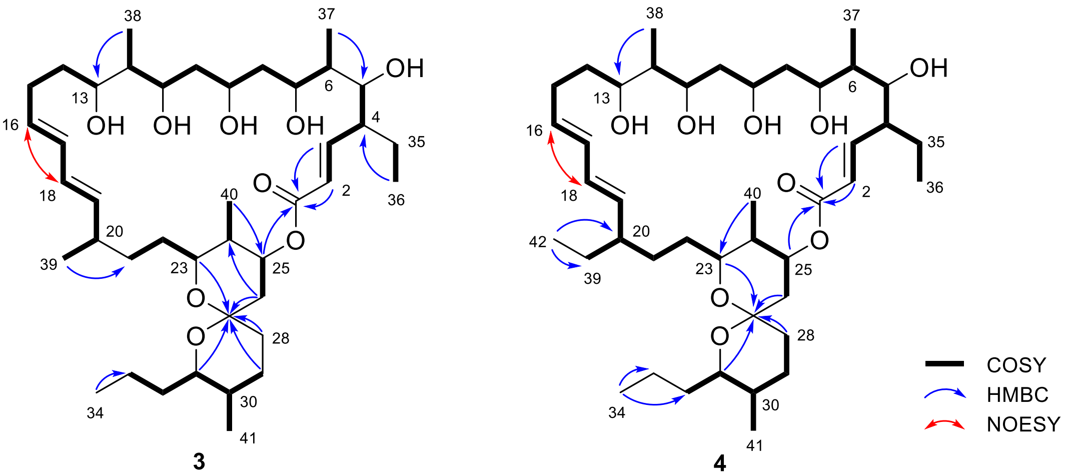

3 **4**
