# Supplementary material for: A cyclopeptide and three oligomycin-class polyketides produced by an underexplored actinomycete of the genus Pseudosporangium
Source: Beilstein J Org Chem. 2020 May 25;16:1100–10. doi: 10.3762/bjoc.16.97 (PMC7277628; doi:10.3762/bjoc.16.97)
Supplement: File 2 — Copies of NMR chemical shifts calculation for compound 1. [file Beilstein_J_Org_Chem-16-1100-s002.pdf]

BEILSTEIN JOURNAL OF ORGANIC CHEMISTRY

Supporting Information

for

A cyclopeptide and three oligomycin-class polyketides produced by an underexplored actinomycete of the genus *Pseudosporangium*

Shun Saito, Kota Atsumi, Tao Zhou, Keisuke Fukaya, Daisuke Urabe, Naoya Oku, Md. Rokon Ul Karim, Hisayuki Komaki and Yasuhiro Igarashi

Beilstein J. Org. Chem. **2020,** *16,* 1100–1110. doi:10.3762/bjoc.16.97

Copies of NMR chemical shifts calculation for compound 1

License and Terms: This is a supporting information file under the terms of the Creative Commons Attribution License (http://creativecommons.org/licenses/by/4.0). Please note that the reuse, redistribution and reproduction in particular requires that the authors and source are credited.
The license is subject to the *Beilstein Journal of Organic Chemistry* terms and conditions: (https://www.beilstein-journals.org/bjoc)

Table of contents

Table S1. Cartesian coordinates of the optimized structures of **1a** and **1b** S3

Table S2. DFT-calculated NMR chemical shifts of structures **1a** and **1b** for **1** S29

Table S1. Cartesian coordinates of the optimized structures of **1a** and **1b**.
Structure, energy and Cartesian coordinate of **1a-1**:

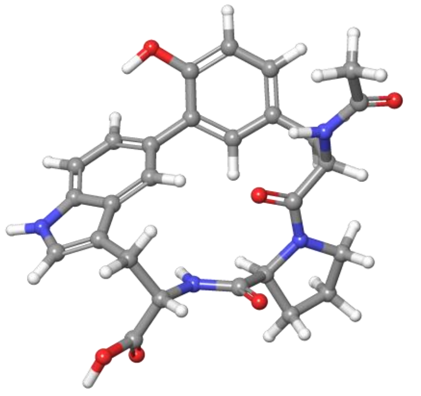

1a-1 (Boltzmann pop.: 62% for DMSO, 61% for MeCN)

M06-2X/6-311+G(d,p)-PCM(DMSO)//B3LYP/6-31G(d):		
Gibbs Free Energy (a.u.)	=	−1715.564661
M06-2X/6-311+G(d,p)-PCM(DMSO):		
Electronic energy (a.u.)	=	−1716.026621
M06-2X/6-311+G(d,p)-PCM(MeCN)//B3LYP/6-31G(d):		
Gibbs Free Energy (a.u.)	=	−1715.564250
M06-2X/6-311+G(d,p)-PCM(MeCN):		
Electronic energy (a.u.)	=	−1716.026210
B3LYP/6-31G(d):		
Zero-point correction (a.u.)	=	0.527591
Thermal correction to Energy (a.u.)	=	0.560389
Thermal correction to Enthalpy (a.u.)	=	0.561333
Thermal correction to Gibbs Free Energy (a.u.)	=	0.461960

O	2.844623	−1.313658	−4.080812
C	4.059239	−1.269240	−2.054917
C	2.942741	−1.237372	0.107367
C	2.439261	0.301364	2.136368
C	4.345012	1.837388	1.939455
O	4.953113	1.280799	2.850999
N	0.071086	−0.209793	2.651668
C	−2.037653	0.855491	1.715601
C	−3.477259	1.626989	−0.139195
O	−5.740241	2.066945	−0.748036
C	−2.946786	2.012696	−1.551231
C	−3.880089	0.540057	−3.446266
O	−2.110181	1.899024	2.348016
C	−0.873949	−1.605110	4.312297
O	0.565606	1.062746	0.837653
C	1.630413	−1.363867	−1.968665
C	−0.561349	−0.227213	−2.161657
C	−2.221453	−0.943225	−3.792466
C	−0.138988	−2.106594	−3.652980
H	4.964459	−1.234911	−2.653390
H	2.413143	−1.881328	2.101576
H	2.639862	0.338234	3.211137
H	5.868035	2.753005	0.743377
H	5.129471	3.828899	1.929274
H	−2.559162	−0.256249	0.062224
H	−6.634459	1.676157	−0.744232
H	−3.577532	2.821092	−1.934409
H	−4.020956	−0.978525	−4.931532
H	−2.288492	0.041730	4.151795
H	−1.037565	−1.690570	5.390391
H	1.281262	−1.382806	3.964559
H	−0.227545	0.436083	−1.369049
H	0.507611	−2.922895	−3.965711

C	2.827423	−1.327098	−2.713319
C	4.112090	−1.203306	−0.664124
C	2.980868	−1.078681	1.612309
N	3.115433	1.424333	1.525602
C	4.916485	3.039020	1.202338
C	0.941210	0.448276	1.839059
C	−1.341267	−0.399147	2.272959
N	−2.651014	0.642410	0.514391
C	−4.920211	1.127997	−0.210371
O	−5.315646	0.042220	0.145004
C	−2.902882	0.879215	−2.541386
N	−3.481510	−0.551743	−4.194620
C	−1.997950	−0.852045	3.589452
C	0.373466	−0.774659	3.982364
C	1.727956	−1.357669	−0.572584
C	0.290003	−1.244834	−2.608769
C	−1.826562	−0.065291	−2.744733
C	−1.388187	−1.967732	−4.251355
H	1.929469	−1.218413	−4.396782
H	5.077915	−1.116924	−0.171743
H	4.011644	−1.148392	1.972572
H	2.622778	1.868044	0.760791
H	4.252600	3.435217	0.428118
H	−1.391618	−1.204404	1.525308
H	−3.457441	2.518767	0.496036
H	−1.942371	2.425222	−1.408066
H	−4.840287	1.005150	−3.624570
H	−2.891240	−1.457618	3.413640
H	−0.768793	−2.618415	3.906751
H	0.517671	0.034768	4.709738
H	0.802036	−1.416385	−0.010823
H	−1.703350	−2.643225	−5.042395

Structure, energy and Cartesian coordinate of **1a-2**:

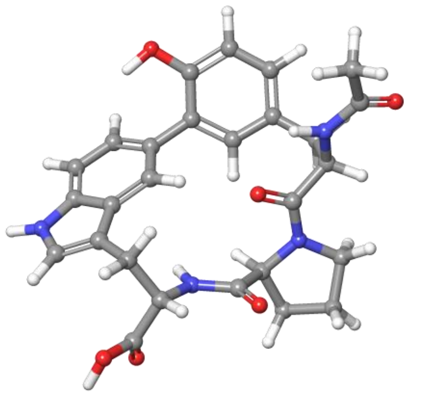

1a-2 (Boltzmann pop.: 25% for DMSO, 25% for MeCN)

M06-2X/6-311+G(d,p)-PCM(DMSO)//B3LYP/6-31G(d):		
Gibbs Free Energy (a.u.)	=	−1715.563792
M06-2X/6-311+G(d,p)-PCM(DMSO):		
Electronic energy (a.u.)	=	−1716.025602
M06-2X/6-311+G(d,p)-PCM(MeCN)//B3LYP/6-31G(d):		
Gibbs Free Energy (a.u.)	=	−1715.563391
M06-2X/6-311+G(d,p)-PCM(MeCN):		
Electronic energy (a.u.)	=	−1716.025201
B3LYP/6-31G(d):		
Zero-point correction (a.u.)	=	0.527658
Thermal correction to Energy (a.u.)	=	0.560453
Thermal correction to Enthalpy (a.u.)	=	0.561398
Thermal correction to Gibbs Free Energy (a.u.)	=	0.461810

O	2.825783	−1.312807	−4.092657
C	4.060179	−1.256462	−2.078981
C	2.965857	−1.243998	0.095289
C	2.482136	0.318676	2.110097
C	4.384163	1.858809	1.907022
O	4.984427	1.321358	2.834836
N	0.119144	−0.224662	2.611352
C	−2.039289	0.717508	1.715957
C	−3.496032	1.552211	−0.105164
O	−5.764768	2.009426	−0.679274
C	−2.987703	1.946355	−1.523161
C	−3.914552	0.451242	−3.402490
O	−2.162791	1.705251	2.427216
C	−1.085799	−0.683245	4.569111
O	0.609285	1.032222	0.786909
C	1.633889	−1.386705	−1.968625
C	−0.571364	−0.269095	−2.148331
C	−2.240657	−1.012333	−3.757402
C	−0.145282	−2.154442	−3.631089
H	4.958651	−1.208245	−2.686593
H	4.047503	−1.142179	1.953856
H	2.685876	0.358526	3.183730
H	5.168499	3.850049	1.866951
H	4.301022	3.430015	0.367373
H	−2.495242	−0.292403	−0.015975
H	−6.663607	1.630465	−0.651506
H	−3.636194	2.741810	−1.903153
H	−4.051623	−1.078217	−4.876465
H	−2.965267	−0.983392	3.451284
H	−1.418519	0.327886	4.821936
H	0.830984	−1.609917	4.070098
H	−0.234395	0.402962	−1.364553
H	0.506444	−2.965979	−3.945529

C	2.822604	−1.330355	−2.725163
C	4.126364	−1.191031	−0.688688
C	3.014999	−1.071246	1.598643
N	3.159149	1.434859	1.489350
C	4.960007	3.047585	1.152645
C	0.986886	0.447834	1.805299
C	−1.244968	−0.526016	2.150788
N	−2.655212	0.556038	0.508807
C	−4.945291	1.065218	−0.148154
O	−5.346802	−0.011483	0.225802
C	−2.932831	0.809005	−2.509705
N	−3.509281	−0.638800	−4.149118
C	−1.898830	−1.210635	3.377854
C	0.340354	−0.627593	4.010795
C	1.746686	−1.384203	−0.573652
C	0.285313	−1.282589	−2.596136
C	−1.845151	−0.123049	−2.719154
C	−1.401257	−2.030859	−4.218407
H	1.905971	−1.233414	−4.399254
H	5.095905	−1.089727	−0.206524
H	2.442990	−1.859654	2.105836
H	2.675997	1.860775	0.708661
H	5.914457	2.753853	0.704650
H	−1.182536	−1.220992	1.304183
H	−3.459583	2.435558	0.541408
H	−1.989013	2.376568	−1.392044
H	−4.882984	0.902178	−3.572450
H	−1.791289	−2.297894	3.289927
H	−1.163162	−1.316387	5.457927
H	0.968946	0.100860	4.528573
H	0.828270	−1.461049	−0.002386
H	−1.717465	−2.714042	−5.002382

Structure, energy and Cartesian coordinate of **1a-3**:

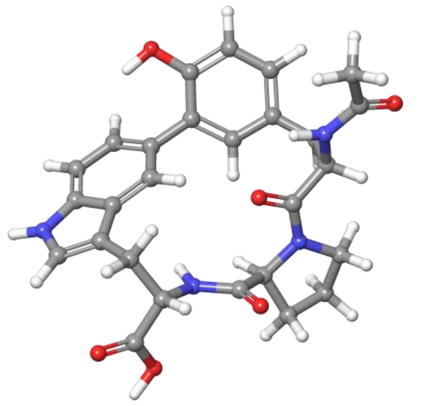

1a-3 (Boltzmann pop.: 8.3% for DMSO, 8.4% for MeCN)

M06-2X/6-311+G(d,p)-PCM(DMSO)//B3LYP/6-31G(d):		
Gibbs Free Energy (a.u.)	=	−1715.562771
M06-2X/6-311+G(d,p)-PCM(DMSO):		
Electronic energy (a.u.)	=	−1716.025409
M06-2X/6-311+G(d,p)-PCM(MeCN)//B3LYP/6-31G(d):		
Gibbs Free Energy (a.u.)	=	−1715.562372
M06-2X/6-311+G(d,p)-PCM(MeCN):		
Electronic energy (a.u.)	=	−1716.025010
B3LYP/6-31G(d):		
Zero-point correction (a.u.)	=	0.527896
Thermal correction to Energy (a.u.)	=	0.560544
Thermal correction to Enthalpy (a.u.)	=	0.561488
Thermal correction to Gibbs Free Energy (a.u.)	=	0.462638

O	2.858912	−1.303206	−4.108272
C	4.078641	−1.210388	−2.085633
C	2.966040	−1.166235	0.078839
C	2.417479	0.371106	2.099960
C	4.281760	1.957275	1.906823
O	4.902014	1.416502	2.819648
N	0.061247	−0.210397	2.602338
C	−2.076556	0.823900	1.700706
C	−3.455628	1.676322	−0.163146
O	−5.347304	0.469966	0.721486
C	−2.929607	2.012771	−1.580499
C	−3.906578	0.470335	−3.401237
O	−2.192780	1.832951	2.380407
C	−0.842276	−1.678351	4.219756
O	0.523653	1.093895	0.804993
C	1.652434	−1.339341	−1.993052
C	−0.543718	−0.219928	−2.190015
C	−2.233327	−0.996052	−3.761762
C	−0.132977	−2.132059	−3.641785
H	4.982306	−1.170550	−2.686104
H	4.034661	−1.027482	1.941480
H	2.612716	0.415747	3.175643
H	4.148279	3.555558	0.399173
H	5.779950	2.910951	0.709246
H	−2.444572	−0.171857	−0.060763
H	−6.315615	0.395485	0.622023
H	−3.560269	2.811688	−1.980040
H	−4.063535	−1.082455	−4.848301
H	−2.324902	−0.090264	4.093528
H	−1.012481	−1.801959	5.293138
H	1.305612	−1.349350	3.912087
H	−0.198578	0.466230	−1.422066
H	0.515678	−2.948767	−3.949218

C	2.846589	−1.296373	−2.740919
C	4.133155	−1.123929	−0.695761
C	3.001658	−0.993729	1.582479
N	3.064818	1.511148	1.489461
C	4.823032	3.173533	1.171111
C	0.916241	0.478970	1.800080
C	−1.343502	−0.430074	2.210901
N	−2.679567	0.644298	0.487703
C	−4.954279	1.361153	−0.214139
O	−5.731939	1.880298	−0.987195
C	−2.906611	0.849082	−2.537621
N	−3.511362	−0.634014	−4.133876
C	−1.990454	−0.952457	3.506998
C	0.370055	−0.785312	3.926670
C	1.752656	−1.314094	−0.597539
C	0.306906	−1.242983	−2.626107
C	−1.822831	−0.084747	−2.748399
C	−1.397068	−2.020384	−4.215608
H	1.941074	−1.228279	−4.422029
H	5.098627	−1.015751	−0.207003
H	2.461837	−1.811498	2.078282
H	2.560326	1.944576	0.726534
H	5.019135	3.966582	1.899282
H	−1.366251	−1.206282	1.431958
H	−3.358738	2.565117	0.473728
H	−1.919077	2.418270	−1.463454
H	−4.882465	0.911697	−3.548951
H	−2.855263	−1.589911	3.303607
H	−0.688719	−2.673775	3.786204
H	0.464097	0.015968	4.671184
H	0.828653	−1.380867	−0.033757
H	−1.721412	−2.718273	−4.983206

Structure, energy and Cartesian coordinate of **1a-4**:

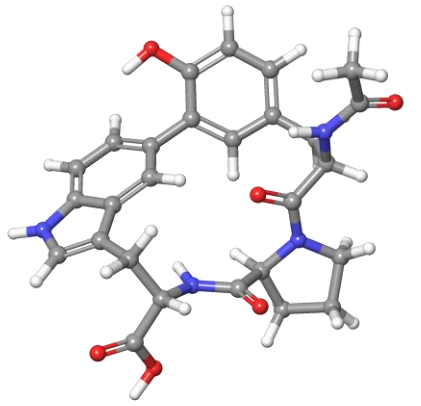

1a-4 (Boltzmann pop.: 3.8% for DMSO, 3.9% for MeCN)

M06-2X/6-311+G(d,p)-PCM(DMSO)//B3LYP/6-31G(d):		
Gibbs Free Energy (a.u.)	=	−1715.562031
M06-2X/6-311+G(d,p)-PCM(DMSO):		
Electronic energy (a.u.)	=	−1716.024512
M06-2X/6-311+G(d,p)-PCM(MeCN)//B3LYP/6-31G(d):		
Gibbs Free Energy (a.u.)	=	−1715.561641
M06-2X/6-311+G(d,p)-PCM(MeCN):		
Electronic energy (a.u.)	=	−1716.024122
B3LYP/6-31G(d):		
Zero-point correction (a.u.)	=	0.527920
Thermal correction to Energy (a.u.)	=	0.560574
Thermal correction to Enthalpy (a.u.)	=	0.561518
Thermal correction to Gibbs Free Energy (a.u.)	=	0.462481

O	2.851292	−1.278222	−4.120827
C	4.082704	−1.192848	−2.105191
C	2.984222	−1.181970	0.067244
C	2.457750	0.373107	2.078342
C	4.325600	1.954988	1.884235
O	4.933742	1.428772	2.813336
N	0.105442	−0.227589	2.567430
C	−2.070417	0.693147	1.693128
C	−3.487089	1.583296	−0.131284
O	−5.353899	0.356870	0.773690
C	−2.977248	1.947284	−1.548297
C	−3.929070	0.403739	−3.378429
O	−2.219326	1.658198	2.429677
C	−1.092210	−0.739047	4.516996
O	0.570290	1.057499	0.757602
C	1.658582	−1.352489	−1.998622
C	−0.551535	−0.251476	−2.181806
C	−2.237761	−1.037711	−3.752100
C	−0.125851	−2.153290	−3.643125
H	4.981813	−1.136637	−2.711171
H	2.472887	−1.805462	2.078014
H	2.656007	0.417693	3.152982
H	4.211956	3.529541	0.350130
H	5.838091	2.883495	0.684875
H	−2.422937	−0.230689	−0.106233
H	−6.321936	0.268006	0.684110
H	−3.628421	2.734271	−1.938344
H	−4.070063	−1.143142	−4.833058
H	−2.964598	−1.056071	3.392608
H	−1.445265	0.260906	4.785894
H	0.843662	−1.618966	4.006735
H	−0.209565	0.435049	−1.412849
H	0.530158	−2.961854	−3.956389

C	2.847393	−1.286584	−2.753460
C	4.145396	−1.117894	−0.715008
C	3.025780	−1.003798	1.570298
N	3.111927	1.504574	1.460799
C	4.878254	3.157231	1.134078
C	0.960601	0.470894	1.770153
C	−1.253418	−0.544386	2.101741
N	−2.687380	0.547402	0.482943
C	−4.981779	1.248210	−0.170169
O	−5.773169	1.753029	−0.938747
C	−2.932017	0.791549	−2.515445
N	−3.521122	−0.690040	−4.119063
C	−1.894088	−1.262512	3.316379
C	0.333063	−0.646199	3.961228
C	1.768773	−1.341688	−0.603598
C	0.308110	−1.264864	−2.624403
C	−1.834960	−0.126581	−2.735100
C	−1.392193	−2.050957	−4.213354
H	1.930528	−1.213706	−4.428219
H	5.112700	−1.000831	−0.231909
H	4.058851	−1.048190	1.928100
H	2.620831	1.923449	0.681307
H	5.071403	3.960996	1.851177
H	−1.180253	−1.222229	1.242160
H	−3.394720	2.460733	0.522143
H	−1.975803	2.375211	−1.432462
H	−4.911790	0.831975	−3.519325
H	−1.764906	−2.345897	3.210483
H	−1.157549	−1.388165	5.395196
H	0.946160	0.087207	4.490468
H	0.850175	−1.428177	−0.034136
H	−1.711800	−2.748162	−4.983557

Structure, energy and Cartesian coordinate of **1a-5**:

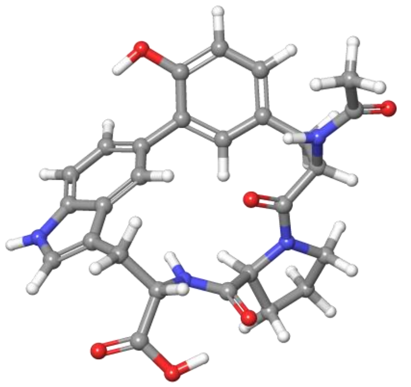

1a-5 (Boltzmann pop.: 0.9% for DMSO, 1.0% for MeCN)

M06-2X/6-311+G(d,p)-PCM(DMSO)//B3LYP/6-31G(d):		
Gibbs Free Energy (a.u.)	=	−1715.560700
M06-2X/6-311+G(d,p)-PCM(DMSO):		
Electronic energy (a.u.)	=	−1716.025481
M06-2X/6-311+G(d,p)-PCM(MeCN)//B3LYP/6-31G(d):		
Gibbs Free Energy (a.u.)	=	−1715.560336
M06-2X/6-311+G(d,p)-PCM(MeCN):		
Electronic energy (a.u.)	=	−1716.025117
B3LYP/6-31G(d):		
Zero-point correction (a.u.)	=	0.528633
Thermal correction to Energy (a.u.)	=	0.560881
Thermal correction to Enthalpy (a.u.)	=	0.561825
Thermal correction to Gibbs Free Energy (a.u.)	=	0.464781

O	2.828712	−1.349894	−4.137280
C	4.045807	−1.216340	−2.111642
C	2.924531	−1.148491	0.049292
C	2.339193	0.333759	2.113284
C	4.189406	1.945357	2.050518
O	4.783703	1.369377	2.958615
N	−0.029427	−0.302845	2.511595
C	−2.192282	0.709429	1.632337
C	−3.200335	1.957018	−0.247603
O	−5.052287	1.900532	1.320227
C	−2.859713	2.099679	−1.745609
C	−3.996058	0.369500	−3.298489
O	−2.563861	1.538678	2.473503
C	−0.909104	−1.998559	3.900392
O	0.465057	1.134413	0.831940
C	1.621284	−1.340613	−2.027520
C	−0.543938	−0.185720	−2.290775
C	−2.330590	−1.107687	−3.664036
C	−0.221813	−2.234925	−3.563991
H	4.952771	−1.186515	−2.707626
H	3.986401	−1.017865	1.917231
H	2.507390	0.333519	3.194824
H	5.720893	2.973247	0.962265
H	4.889141	3.965042	2.159371
H	−2.180329	0.099624	−0.315459
H	−4.235714	1.914446	1.881586
H	−3.538906	2.853205	−2.152954
H	−4.252790	−1.326349	−4.556717
H	−2.511498	−0.526034	3.869461
H	−1.129234	−2.263683	4.938282
H	1.218450	−1.469977	3.790668
H	−0.151469	0.564847	−1.610227
H	0.407716	−3.080991	−3.828406

C	2.816471	−1.313920	−2.771319
C	4.094354	−1.114243	−0.722131
C	2.953602	−1.000805	1.556454
N	2.993083	1.504036	1.570357
C	4.739843	3.200191	1.390984
C	0.843822	0.448700	1.785392
C	−1.402904	−0.549536	2.025909
N	−2.557165	0.776998	0.335305
C	−4.722191	1.928709	0.019943
O	−5.559234	1.953487	−0.853589
C	−2.931590	0.842454	−2.571354
N	−3.640221	−0.791346	−3.960669
C	−2.071919	−1.277934	3.206301
C	0.238769	−0.988346	3.791554
C	1.714302	−1.295421	−0.632334
C	0.272470	−1.261493	−2.658341
C	−1.854069	−0.096719	−2.782610
C	−1.518003	−2.169759	−4.072278
H	1.909012	−1.305071	−4.451363
H	5.058647	−1.005616	−0.231193
H	2.436750	−1.845264	2.031659
H	2.503535	1.973533	0.819411
H	4.092900	3.603347	0.606069
H	−1.341229	−1.208610	1.148332
H	−2.806658	2.831534	0.287829
H	−1.844985	2.508622	−1.814102
H	−4.994171	0.775450	−3.382735
H	−2.866907	−1.949997	2.871430
H	−0.650314	−2.919188	3.364070
H	0.212607	−0.264436	4.616484
H	0.787140	−1.356918	−0.073257
H	−1.886858	−2.937622	−4.747264

Structure, energy and Cartesian coordinate of **1a-6**:

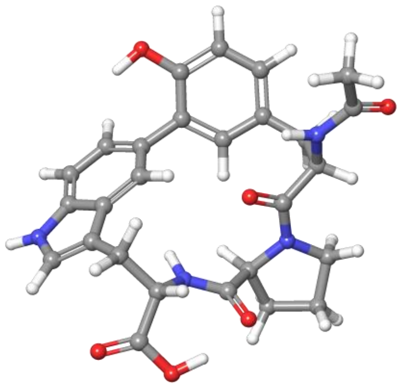

1a-6 (Boltzmann pop.: 0.6% for DMSO, 0.6% for MeCN)

M06-2X/6-311+G(d,p)-PCM(DMSO)//B3LYP/6-31G(d):		
Gibbs Free Energy (a.u.)	=	−1715.560277
M06-2X/6-311+G(d,p)-PCM(DMSO):		
Electronic energy (a.u.)	=	−1716.024731
M06-2X/6-311+G(d,p)-PCM(MeCN)//B3LYP/6-31G(d):		
Gibbs Free Energy (a.u.)	=	−1715.559920
M06-2X/6-311+G(d,p)-PCM(MeCN):		
Electronic energy (a.u.)	=	−1716.024374
B3LYP/6-31G(d):		
Zero-point correction (a.u.)	=	0.528592
Thermal correction to Energy (a.u.)	=	0.560874
Thermal correction to Enthalpy (a.u.)	=	0.561818
Thermal correction to Gibbs Free Energy (a.u.)	=	0.464454

O	2.836298	−1.329072	−4.158155
C	4.056101	−1.210872	−2.133375
C	2.939089	−1.175207	0.030694
C	2.371559	0.333119	2.075617
C	4.228470	1.935907	1.989455
O	4.812029	1.381436	2.917462
N	0.009313	−0.301760	2.489153
C	−2.194380	0.586084	1.631574
C	−3.245051	1.854440	−0.219206
O	−5.093415	1.684389	1.344154
C	−2.904825	2.032962	−1.714109
C	−4.005991	0.310061	−3.295075
O	−2.609030	1.358856	2.507446
C	−1.195368	−1.056760	4.354940
O	0.498824	1.085897	0.765762
C	1.632614	−1.357564	−2.046219
C	−0.544906	−0.216794	−2.295334
C	−2.322039	−1.140051	−3.679506
C	−0.203095	−2.248865	−3.591485
H	4.961452	−1.166889	−2.730942
H	3.997581	−1.051068	1.902104
H	2.545260	0.342677	3.155825
H	4.959275	3.946486	2.049441
H	4.146109	3.563416	0.509832
H	−2.151304	0.046387	−0.332432
H	−4.277939	1.704246	1.908090
H	−3.600574	2.777417	−2.109980
H	−4.241287	−1.372641	−4.574408
H	−3.033324	−1.335403	3.171229
H	−1.600356	−0.108280	4.719779
H	0.785159	−1.795252	3.800989
H	−0.157975	0.530136	−1.607697
H	0.434205	−3.086133	−3.865103

C	2.826156	−1.310749	−2.791837
C	4.106845	−1.122153	−0.742996
C	2.966548	−1.019277	1.537175
N	3.031945	1.489680	1.513331
C	4.792618	3.167753	1.298916
C	0.879350	0.438740	1.744482
C	−1.326993	−0.636881	1.963815
N	−2.557946	0.687183	0.337006
C	−4.764935	1.768070	0.046063
O	−5.602322	1.798313	−0.826992
C	−2.948139	0.787757	−2.561278
N	−3.635157	−0.836298	−3.972996
C	−1.954909	−1.490831	3.092538
C	0.232172	−0.846106	3.840352
C	1.729440	−1.329444	−0.650829
C	0.281652	−1.281599	−2.674344
C	−1.857048	−0.134429	−2.785317
C	−1.499195	−2.189132	−4.100164
H	1.915617	−1.289703	−4.470088
H	5.071137	−1.008029	−0.253259
H	2.429233	−1.844299	2.023892
H	2.556617	1.936648	0.739967
H	5.766923	2.916833	0.867946
H	−1.204346	−1.227370	1.047739
H	−2.883293	2.732935	0.332653
H	−1.899643	2.466476	−1.772185
H	−5.009767	0.703160	−3.372413
H	−1.778769	−2.551888	2.882949
H	−1.243593	−1.795548	5.160031
H	0.803464	−0.141706	4.450045
H	0.804751	−1.407239	−0.090289
H	−1.860893	−2.952170	−4.784435

Structure, energy and Cartesian coordinate of **1b-1**:

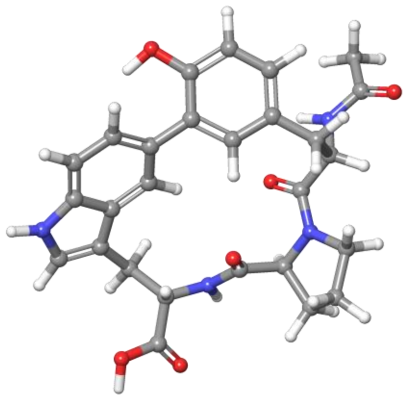

1b-1 (Boltzmann pop.: 45% for DMSO, 46% for MeCN)

M06-2X/6-311+G(d,p)-PCM(DMSO)//B3LYP/6-31G(d):		
Gibbs Free Energy (a.u.)	=	−1715.565298
M06-2X/6-311+G(d,p)-PCM(DMSO):		
Electronic energy (a.u.)	=	−1716.026218
M06-2X/6-311+G(d,p)-PCM(MeCN)//B3LYP/6-31G(d):		
Gibbs Free Energy (a.u.)	=	−1715.564875
M06-2X/6-311+G(d,p)-PCM(MeCN):		
Electronic energy (a.u.)	=	−1716.025795
B3LYP/6-31G(d):		
Zero-point correction (a.u.)	=	0.527662
Thermal correction to Energy (a.u.)	=	0.560595
Thermal correction to Enthalpy (a.u.)	=	0.561539
Thermal correction to Gibbs Free Energy (a.u.)	=	0.460920

O	2.556032	1.344852	−4.278981
C	3.829894	1.240121	−2.296792
C	2.781234	1.164055	−0.104334
C	2.740415	−0.416249	1.953204
C	4.955258	−1.454656	1.690789
O	5.440134	−0.781586	2.597714
N	0.394276	−0.673587	2.708218
C	−1.740408	−0.475421	1.468517
C	−3.698938	−0.652654	−0.019598
O	−6.029075	−0.182862	−0.129095
C	−3.606456	−1.224523	−1.487322
C	−4.396001	0.333803	−3.385760
O	−1.521810	0.694378	1.175983
C	−0.865007	0.341268	4.425527
O	1.054093	−1.708701	0.808158
C	1.403146	1.344713	−2.127521
C	−0.936793	0.512544	−2.273802
C	−2.521661	1.500334	−3.838531
C	−0.280997	2.318903	−3.763961
H	4.714281	1.225843	−2.926570
H	2.137735	1.677988	1.883439
H	3.003166	−0.381522	3.015309
H	5.198796	−3.025428	0.166990
H	6.215907	−3.183961	1.623286
H	−2.943672	−2.131981	1.278364
H	−6.887390	−0.411896	0.275815
H	−2.744495	−1.900940	−1.501366
H	−4.349340	1.854949	−4.875990
H	−2.672050	−0.830755	3.918661
H	−1.305791	1.213965	3.934475
H	1.182952	−0.398859	4.660594
H	−0.685245	−0.204030	−1.498193
H	0.469068	3.036139	−4.086915

C	2.576949	1.316618	−2.909930
C	3.927963	1.164355	−0.910500
C	2.887259	1.040895	1.398335
N	3.652289	−1.342433	1.313566
C	5.776355	−2.474560	0.915434
C	1.328621	−0.986345	1.769779
C	−0.959638	−1.208053	2.575014
N	−2.722830	−1.225561	0.885550
C	−5.091485	−0.882741	0.547659
O	−5.358597	−1.627037	1.466800
C	−3.442720	−0.165302	−2.534484
N	−3.849882	1.329515	−4.175880
C	−1.584099	−0.936093	3.963902
C	0.571854	0.139862	3.922921
C	1.537433	1.267113	−0.733203
C	0.047248	1.388972	−2.739505
C	−2.230551	0.568230	−2.806725
C	−1.552579	2.381032	−4.325857
H	1.632518	1.258229	−4.571330
H	4.910216	1.105702	−0.447511
H	3.870458	1.384243	1.735920
H	3.260420	−1.883640	0.553196
H	6.602652	−1.958272	0.416502
H	−0.911037	−2.275402	2.336577
H	−3.499139	0.421310	−0.049164
H	−4.488004	−1.836165	−1.708672
H	−5.437421	0.060724	−3.489042
H	−1.354661	−1.773002	4.633716
H	−0.908045	0.491916	5.507977
H	1.066002	1.088393	3.699561
H	0.633341	1.295765	−0.129817
H	−1.782148	3.107485	−5.101101

Structure, energy and Cartesian coordinate of **1b-2**:

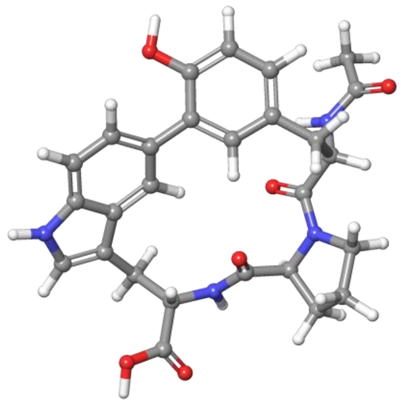

1b-2 (Boltzmann pop.: 19% for DMSO, 18% for MeCN)

M06-2X/6-311+G(d,p)-PCM(DMSO)//B3LYP/6-31G(d):		
Gibbs Free Energy (a.u.)	=	−1715.564477
M06-2X/6-311+G(d,p)-PCM(DMSO):		
Electronic energy (a.u.)	=	−1716.024846
M06-2X/6-311+G(d,p)-PCM(MeCN)//B3LYP/6-31G(d):		
Gibbs Free Energy (a.u.)	=	−1715.564007
M06-2X/6-311+G(d,p)-PCM(MeCN):		
Electronic energy (a.u.)	=	−1716.024376
B3LYP/6-31G(d):		
Zero-point correction (a.u.)	=	0.527308
Thermal correction to Energy (a.u.)	=	0.560405
Thermal correction to Enthalpy (a.u.)	=	0.561350
Thermal correction to Gibbs Free Energy (a.u.)	=	0.460369

O	2.414790	1.090007	−4.435208
C	3.793430	1.003233	−2.459265
C	2.768750	1.018207	−0.262736
C	2.732542	−0.442378	1.887740
C	4.898986	−1.535813	1.476853
O	5.489514	−0.789085	2.254702
N	0.432507	−0.556530	2.799878
C	−1.725530	−0.291432	1.625136
C	−3.725695	−0.429033	0.189396
O	−6.005822	0.241098	0.066283
C	−3.704144	−1.147513	−1.215043
C	−4.510772	0.237023	−3.237337
O	−1.421670	0.830826	1.236703
C	−0.683165	0.659080	4.486162
O	0.929112	−1.724395	0.926816
C	1.365352	1.130072	−2.284902
C	−1.004509	0.391602	−2.267730
C	−2.608953	1.267189	−3.875063
C	−0.330513	1.972049	−3.985353
H	4.687109	0.967041	−3.081520
H	2.155734	1.651739	1.698373
H	3.062441	−0.357646	2.928251
H	6.123358	−3.291924	1.429631
H	6.395540	−2.183373	0.086174
H	−3.062261	−1.854783	1.596891
H	−6.874572	0.117444	0.494181
H	−2.861899	−1.848127	−1.186812
H	−4.456427	1.579197	−4.890766
H	−2.584025	−0.415300	4.130353
H	−1.084811	1.524579	3.950800
H	1.319322	−0.203544	4.696057
H	−0.756669	−0.247871	−1.426265
H	0.450379	2.562562	−4.449715
C	2.538199	1.078643	−3.067705
C	3.908995	0.968302	−1.071306
C	2.891152	0.977522	1.243497
N	3.561779	−1.442052	1.246235
C	5.620242	−2.636446	0.712073
C	1.291476	−0.962007	1.826648
C	−0.958761	−1.003089	2.755933
N	−2.787099	−0.996326	1.137038
C	−5.123052	−0.486555	0.785536
O	−5.441326	−1.134610	1.760160
C	−3.544966	−0.207383	−2.370070
N	−3.955895	1.121343	−4.145626
C	−1.504820	−0.593689	4.143604
C	0.714613	0.324713	3.945546
C	1.521194	1.109879	−0.889829
C	−0.003267	1.159096	−2.868413
C	−2.315882	0.442083	−2.759666
C	−1.619303	2.035177	−4.497908
H	3.300956	1.008875	−4.820717
H	4.894701	0.914551	−0.615604
H	3.883681	1.326926	1.544046
H	3.087105	−2.025409	0.568188
H	4.959346	−3.237556	0.080332
H	−0.993905	−2.084873	2.591851
H	−3.433773	0.614860	0.053208
H	−4.609393	−1.752317	−1.340982
H	−5.564889	−0.001823	−3.276614
H	−1.305003	−1.396348	4.863060
H	−0.670427	0.886308	5.555969
H	1.260587	1.219464	3.637656
H	0.626550	1.180174	−0.276797
H	−1.846245	2.668363	−5.352010

Structure, energy and Cartesian coordinate of **1b-3**:

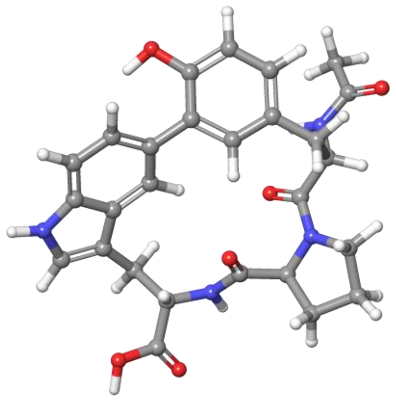

1b-3 (Boltzmann pop.: 18% for DMSO, 18% for MeCN)

M06-2X/6-311+G(d,p)-PCM(DMSO)//B3LYP/6-31G(d):		
Gibbs Free Energy (a.u.)	=	−1715.564404
M06-2X/6-311+G(d,p)-PCM(DMSO):		
Electronic energy (a.u.)	=	−1716.025649
M06-2X/6-311+G(d,p)-PCM(MeCN)//B3LYP/6-31G(d):		
Gibbs Free Energy (a.u.)	=	−1715.563985
M06-2X/6-311+G(d,p)-PCM(MeCN):		
Electronic energy (a.u.)	=	−1716.025230
B3LYP/6-31G(d):		
Zero-point correction (a.u.)	=	0.527693
Thermal correction to Energy (a.u.)	=	0.560583
Thermal correction to Enthalpy (a.u.)	=	0.561528
Thermal correction to Gibbs Free Energy (a.u.)	=	0.461245

O	2.765105	1.233358	−4.222504
C	3.953321	1.215400	−2.185446
C	2.812620	1.181113	−0.038707
C	2.610275	−0.373989	2.020320
C	4.732013	−1.538866	1.595673
O	5.334144	−0.857243	2.423125
N	0.254756	−0.325435	2.811160
C	−1.831508	−0.258894	1.457973
C	−3.704374	−0.675504	−0.084706
O	−6.035763	−0.389604	−0.463377
C	−3.415845	−1.369550	−1.474400
C	−4.186898	0.002207	−3.525403
O	−1.566265	0.831442	0.966136
C	−0.617895	0.181150	4.927153
O	0.783587	−1.604648	1.027267
C	1.519955	1.283073	−2.122943
C	−0.785360	0.381714	−2.301251
C	−2.335386	1.201601	−3.992060
C	−0.127136	2.103575	−3.886100
H	4.863898	1.195237	−2.776610
H	2.135839	1.752796	1.930148
H	2.920261	−0.367272	3.070610
H	6.100748	−2.132141	0.055900
H	4.735270	−3.258843	0.224182
H	−3.060582	−1.914455	1.495676
H	−6.913905	−0.662846	−0.135637
H	−2.495370	−1.948470	−1.343668
H	−4.130605	1.410768	−5.122362
H	−2.279036	0.833281	3.696374
H	−0.832917	0.974031	5.649553
H	1.500870	0.401610	4.370808
H	−0.546194	−0.269822	−1.467115
H	0.609397	2.826548	−4.227190

C	2.726736	1.251949	−2.853532
C	3.993033	1.175251	−0.794834
C	2.860817	1.071454	1.468119
N	3.404726	−1.377718	1.345098
C	5.427581	−2.613536	0.773460
C	1.145082	−0.817179	1.906340
C	−1.144891	−0.756816	2.739174
N	−2.804806	−1.084505	0.975784
C	−5.129089	−0.971080	0.352737
O	−5.440755	−1.676845	1.288474
C	−3.252359	−0.398040	−2.603642
N	−3.642511	0.957632	−4.365973
C	−1.798958	−0.107447	3.987305
C	0.503897	0.561763	3.954469
C	1.595950	1.253151	−0.721671
C	0.189412	1.256482	−2.788474
C	−2.056502	0.357122	−2.885026
C	−1.375970	2.081152	−4.500912
H	1.858154	1.109140	−4.551007
H	4.954358	1.133229	−0.287870
H	3.852720	1.358670	1.829464
H	2.915260	−1.920956	0.644875
H	6.041366	−3.224656	1.440830
H	−1.182711	−1.852354	2.779029
H	−3.577830	0.402956	−0.207045
H	−4.208229	−2.090538	−1.704548
H	−5.214261	−0.313150	−3.646761
H	−2.561138	−0.753510	4.431123
H	−0.337479	−0.720688	5.483638
H	0.421552	1.614019	3.651049
H	0.666290	1.288962	−0.159192
H	−1.596461	2.744760	−5.333059

Structure, energy and Cartesian coordinate of **1b-4**:

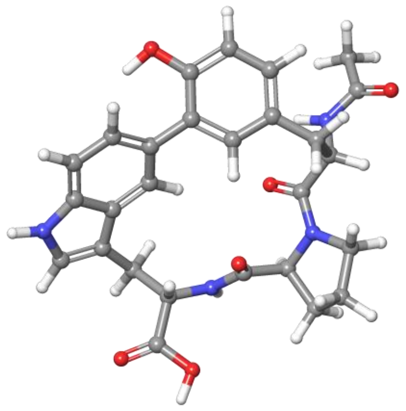

1b-4 (Boltzmann pop.: 12% for DMSO, 12% for MeCN)

M06-2X/6-311+G(d,p)-PCM(DMSO)//B3LYP/6-31G(d):		
Gibbs Free Energy (a.u.)	=	−1715.564034
M06-2X/6-311+G(d,p)-PCM(DMSO):		
Electronic energy (a.u.)	=	−1716.025054
M06-2X/6-311+G(d,p)-PCM(MeCN)//B3LYP/6-31G(d):		
Gibbs Free Energy (a.u.)	=	−1715.563604
M06-2X/6-311+G(d,p)-PCM(MeCN):		
Electronic energy (a.u.)	=	−1716.024624
B3LYP/6-31G(d):		
Zero-point correction (a.u.)	=	0.527791
Thermal correction to Energy (a.u.)	=	0.560674
Thermal correction to Enthalpy (a.u.)	=	0.561618
Thermal correction to Gibbs Free Energy (a.u.)	=	0.461020

O	2.651547	1.561923	−4.186830
C	3.871906	1.333605	−2.181742
C	2.765484	1.129133	−0.026094
C	2.656322	−0.586811	1.917330
C	4.845286	−1.647332	1.550213
O	5.354405	−1.051067	2.496612
N	0.318590	−0.855796	2.690714
C	−1.817349	−0.504831	1.493664
C	−3.658835	−0.531617	−0.142334
O	−5.225418	−0.431814	1.678290
C	−3.560949	−1.105032	−1.589937
C	−4.335461	0.504710	−3.462338
O	−1.615130	0.695138	1.348661
C	−0.893898	0.064994	4.492609
O	0.918788	−1.735414	0.695650
C	1.441858	1.442765	−2.070353
C	−0.891898	0.624642	−2.303096
C	−2.448217	1.675606	−3.856763
C	−0.207219	2.488070	−3.705619
H	4.772489	1.351794	−2.788025
H	2.079156	1.515918	1.977681
H	2.929778	−0.634828	2.975953
H	6.110333	−3.360696	1.334375
H	6.440093	−2.067593	0.181869
H	−2.867281	−2.186828	0.947118
H	−6.180164	−0.341071	1.860319
H	−2.694491	−1.776288	−1.617711
H	−4.266076	2.081700	−4.893087
H	−2.743747	−0.994134	3.905418
H	−1.307692	0.986317	4.072942
H	1.129251	−0.760537	4.650761
H	−0.655194	−0.125916	−1.554637
H	0.546754	3.219990	−3.983571

C	2.635902	1.454041	−2.821999
C	3.933123	1.171178	−0.800871
C	2.833025	0.904220	1.467011
N	3.538125	−1.488309	1.204628
C	5.638171	−2.616644	0.685937
C	1.228809	−1.106288	1.711264
C	−1.051667	−1.340724	2.532753
N	−2.783880	−1.188560	0.811667
C	−5.112440	−0.475253	0.329435
O	−6.073583	−0.435378	−0.406886
C	−3.392289	−0.031061	−2.623437
N	−3.773119	1.522336	−4.214955
C	−1.661279	−1.147297	3.941306
C	0.531846	−0.145538	3.963217
C	1.540045	1.281843	−0.679768
C	0.101444	1.518795	−2.711894
C	−2.174773	0.704094	−2.857088
C	−1.468097	2.572171	−4.290479
H	1.736213	1.493725	−4.508597
H	4.902355	1.075943	−0.316916
H	3.812299	1.211223	1.847905
H	3.131611	−1.952835	0.402529
H	5.032410	−3.128579	−0.067849
H	−1.035014	−2.390924	2.222647
H	−3.311230	0.508673	−0.147642
H	−4.449195	−1.704713	−1.807279
H	−5.380261	0.248906	−3.573321
H	−1.463767	−2.039616	4.546847
H	−0.924555	0.132704	5.583863
H	1.057024	0.799978	3.808560
H	0.621138	1.283388	−0.098503
H	−1.681415	3.329799	−5.040123

Structure, energy and Cartesian coordinate of **1b-5**:

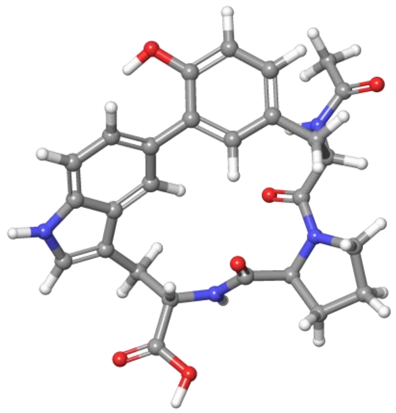

1b-5 (Boltzmann pop.: 4.5% for DMSO, 4.5% for MeCN)

M06-2X/6-311+G(d,p)-PCM(DMSO)//B3LYP/6-31G(d):		
Gibbs Free Energy (a.u.)	=	−1715.563120
M06-2X/6-311+G(d,p)-PCM(DMSO):		
Electronic energy (a.u.)	=	−1716.024105
M06-2X/6-311+G(d,p)-PCM(MeCN)//B3LYP/6-31G(d):		
Gibbs Free Energy (a.u.)	=	−1715.562695
M06-2X/6-311+G(d,p)-PCM(MeCN):		
Electronic energy (a.u.)	=	−1716.023680
B3LYP/6-31G(d):		
Zero-point correction (a.u.)	=	0.527772
Thermal correction to Energy (a.u.)	=	0.560644
Thermal correction to Enthalpy (a.u.)	=	0.561588
Thermal correction to Gibbs Free Energy (a.u.)	=	0.460985

O	2.785366	1.266813	−4.226947	C	2.717188	1.289693	−2.859490
C	3.930364	1.289612	−2.165878	C	3.943165	1.249180	−0.774832
C	2.747480	1.221191	−0.043047	C	2.768888	1.097036	1.463158
C	2.555390	−0.366033	1.991466	N	3.347971	−1.346490	1.280589
C	4.691532	−1.461560	1.465062	C	5.386003	−2.481450	0.575256
O	5.304677	−0.777957	2.282432	C	1.094653	−0.824606	1.897827
N	0.225425	−0.395264	2.852587	C	−1.174062	−0.828747	2.785313
C	−1.881648	−0.248599	1.552103	N	−2.817289	−1.063751	0.981364
C	−3.619237	−0.623428	−0.146799	C	−5.111598	−0.562744	0.179767
O	−5.352488	−0.245028	1.473042	O	−6.000605	−0.739314	−0.624644
C	−3.368036	−1.419705	−1.463593	C	−3.223835	−0.513711	−2.650869
C	−4.161834	−0.159041	−3.586218	N	−3.626652	0.774682	−4.459170
O	−1.670305	0.898967	1.179477	C	−1.805149	−0.258099	4.083087
C	−0.604896	0.008944	5.005665	C	0.487774	0.448886	4.025026
O	0.715508	−1.568809	0.987799	C	1.544373	1.263566	−0.751223
C	1.494897	1.288956	−2.154248	C	0.178663	1.214039	−2.843550
C	−0.780563	0.325423	−2.349936	C	−2.041408	0.252911	−2.950505
C	−2.327711	1.058033	−4.084657	C	−1.382157	1.947020	−4.603007
C	−0.143993	2.021622	−3.968961	H	1.890723	1.109358	−4.574552
H	4.852957	1.295081	−2.738458	H	4.894378	1.230811	−0.247804
H	2.011265	1.746627	1.918655	H	3.743544	1.413019	1.845980
H	2.886930	−0.375048	3.034634	H	2.855810	−1.867391	0.565358
H	6.158859	−2.988579	1.157823	H	4.700679	−3.222742	0.152477
H	5.879853	−1.957232	−0.250766	H	−1.205717	−1.925051	2.760351
H	−2.881454	−2.025205	1.286590	H	−3.306410	0.416635	−0.300108
H	−6.323270	−0.180246	1.552214	H	−4.179811	−2.135439	−1.621133
H	−2.443113	−1.991980	−1.328222	H	−5.186948	−0.487064	−3.689458
H	−4.123362	1.202367	−5.224754	H	−2.539805	−0.943961	4.513256
H	−2.314616	0.683194	3.852572	H	−0.296712	−0.910798	5.516302
H	−0.814345	0.766762	5.766284	H	0.381197	1.510588	3.765828
H	1.496554	0.288044	4.411379	H	0.603809	1.277530	−0.206247
H	−0.535944	−0.303227	−1.499027	H	−1.605995	2.582984	−5.455612
H	0.575094	2.758726	−4.317011				

Structure, energy and Cartesian coordinate of **1b-6**:

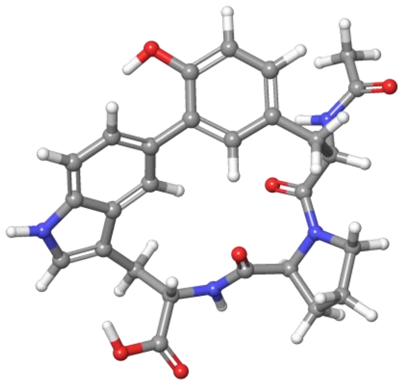

1b-6 (Boltzmann pop.: 1.4% for DMSO, 1.4% for MeCN)

M06-2X/6-311+G(d,p)-PCM(DMSO)//B3LYP/6-31G(d):		
Gibbs Free Energy (a.u.)	=	−1715.562032
M06-2X/6-311+G(d,p)-PCM(DMSO):		
Electronic energy (a.u.)	=	−1716.024399
M06-2X/6-311+G(d,p)-PCM(MeCN)//B3LYP/6-31G(d):		
Gibbs Free Energy (a.u.)	=	−1715.561608
M06-2X/6-311+G(d,p)-PCM(MeCN):		
Electronic energy (a.u.)	=	−1716.023975
B3LYP/6-31G(d):		
Zero-point correction (a.u.)	=	0.527919
Thermal correction to Energy (a.u.)	=	0.560642
Thermal correction to Enthalpy (a.u.)	=	0.561587
Thermal correction to Gibbs Free Energy (a.u.)	=	0.462367

O	2.552250	1.438834	−4.222867
C	3.785904	1.316326	−2.217619
C	2.698261	1.161135	−0.048205
C	2.679148	−0.470246	1.979178
C	4.908573	−1.430474	1.577446
O	5.425149	−0.737433	2.451220
N	0.372774	−0.804377	2.825696
C	−1.724966	−0.541975	1.562041
C	−3.602722	−0.585397	−0.002520
O	−5.872406	0.194830	−0.349075
C	−3.530497	−1.271224	−1.417268
C	−4.394731	0.168525	−3.412188
O	−1.398213	0.589486	1.220525
C	−0.859375	0.258045	4.534563
O	0.965987	−1.744962	0.855832
C	1.355097	1.368167	−2.093974
C	−0.963084	0.496170	−2.250499
C	−2.551756	1.390094	−3.871059
C	−0.335774	2.276198	−3.777730
H	4.681813	1.334944	−2.830695
H	2.003019	1.603694	1.938911
H	2.978019	−0.452672	3.031994
H	6.246577	−3.094374	1.424047
H	6.471919	−1.867296	0.178163
H	−3.119948	−2.058095	1.436783
H	−5.361590	0.559062	−1.098542
H	−2.632735	−1.898556	−1.417281
H	−4.369585	1.629570	−4.962209
H	−2.706384	−0.841482	4.014320
H	−1.263083	1.146632	4.039588
H	1.156421	−0.562031	4.783537
H	−0.706996	−0.195604	−1.454667
H	0.397102	3.006638	−4.110146

C	2.543594	1.381489	−2.853935
C	3.859921	1.206498	−0.832423
C	2.782543	1.004251	1.453551
N	3.579725	−1.370762	1.288787
C	5.716003	−2.417649	0.747490
C	1.270305	−1.058458	1.834872
C	−0.997900	−1.295227	2.689683
N	−2.768753	−1.218654	0.993753
C	−5.054740	−0.510633	0.468507
O	−5.478141	−1.057271	1.457510
C	−3.435483	−0.262599	−2.523679
N	−3.867884	1.158518	−4.225532
C	−1.624234	−0.991883	4.070905
C	0.572232	0.001302	4.042551
C	1.465659	1.257886	−0.698166
C	0.007122	1.382002	−2.725145
C	−2.247676	0.506039	−2.805384
C	−1.598568	2.286665	−4.364209
H	1.643569	1.307908	−4.542000
H	4.834112	1.155427	−0.351902
H	3.748328	1.374184	1.811724
H	3.165395	−1.908100	0.537552
H	5.109088	−3.006671	0.053296
H	−0.990203	−2.364976	2.456819
H	−3.207240	0.430966	−0.095468
H	−4.384494	−1.942635	−1.563593
H	−5.410391	−0.176636	−3.557935
H	−1.435843	−1.833860	4.746933
H	−0.905315	0.413009	5.616183
H	1.104536	0.929930	3.824791
H	0.552551	1.253344	−0.108920
H	−1.831985	2.984443	−5.163916

Structure, energy and Cartesian coordinate of **1b-7**:

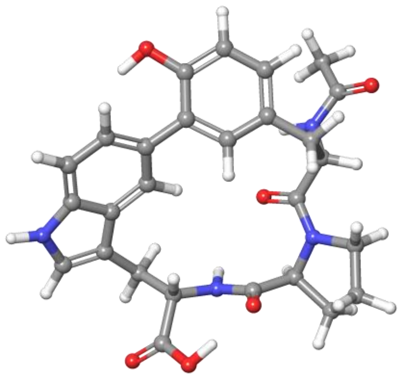

1b-7 (Boltzmann pop.: 0.3% for DMSO, 0.3% for MeCN)

M06-2X/6-311+G(d,p)-PCM(DMSO)//B3LYP/6-31G(d):		
Gibbs Free Energy (a.u.)	=	−1715.560566
M06-2X/6-311+G(d,p)-PCM(DMSO):		
Electronic energy (a.u.)	=	−1716.024374
M06-2X/6-311+G(d,p)-PCM(MeCN)//B3LYP/6-31G(d):		
Gibbs Free Energy (a.u.)	=	−1715.560202
M06-2X/6-311+G(d,p)-PCM(MeCN):		
Electronic energy (a.u.)	=	−1716.024010
B3LYP/6-31G(d):		
Zero-point correction (a.u.)	=	0.528836
Thermal correction to Energy (a.u.)	=	0.561149
Thermal correction to Enthalpy (a.u.)	=	0.562093
Thermal correction to Gibbs Free Energy (a.u.)	=	0.463808

O	2.659791	1.421307	−4.319721
C	3.847151	1.303936	−2.284778
C	2.707200	1.159830	−0.142446
C	2.488807	−0.519684	1.818870
C	4.575814	−1.652489	1.187467
O	5.222497	−1.031986	2.027901
N	0.222790	−0.609028	2.819434
C	−2.133819	−0.418304	2.037912
C	−3.462188	−0.421644	−0.047740
O	−5.034481	0.619829	1.502748
C	−3.444620	−1.208983	−1.378115
C	−4.337476	0.136548	−3.416895
O	−2.471676	0.741908	2.319080
C	−0.662769	0.176159	4.864380
O	0.565430	−1.587768	0.807098
C	1.414251	1.370830	−2.222827
C	−0.881488	0.470104	−2.366373
C	−2.494699	1.310362	−3.987917
C	−0.277242	2.207536	−3.951711
H	4.757879	1.312849	−2.875827
H	2.012611	1.613715	1.849756
H	2.849592	−0.587653	2.849960
H	4.505981	−3.339576	−0.216489
H	5.990764	−3.200586	0.766900
H	−2.200980	−1.958273	0.728132
H	−4.135130	0.882159	1.830796
H	−2.571650	−1.876037	−1.387288
H	−4.351945	1.553510	−5.007442
H	−2.547351	−0.933417	4.582465
H	−1.186364	1.090142	4.573728
H	1.372029	−0.581903	4.606118
H	−0.610750	−0.205114	−1.559377
H	0.442782	2.938929	−4.309810

C	2.620983	1.374531	−2.953140
C	3.888274	1.189471	−0.898090
C	2.741940	0.960110	1.354663
N	3.230719	−1.489111	1.039673
C	5.221235	−2.641441	0.229432
C	1.014491	−0.944654	1.763025
C	−1.131391	−1.184666	2.912862
N	−2.658126	−1.093896	0.991484
C	−4.919350	−0.171371	0.430497
O	−5.885654	−0.627776	−0.136096
C	−3.358066	−0.279457	−2.552100
N	−3.822018	1.079626	−4.292780
C	−1.474300	−1.047966	4.411767
C	0.644742	0.046158	4.073646
C	1.496579	1.275846	−0.825106
C	0.076516	1.353868	−2.871148
C	−2.172232	0.457103	−2.900428
C	−1.548094	2.187876	−4.524605
H	1.755722	1.300977	−4.657463
H	4.849196	1.115534	−0.394367
H	3.730638	1.225006	1.739783
H	2.715488	−1.941479	0.295185
H	5.713218	−2.087720	−0.578239
H	−1.091450	−2.229360	2.589379
H	−3.009247	0.566038	−0.198729
H	−4.341896	−1.830198	−1.429130
H	−5.377864	−0.153595	−3.469470
H	−1.139827	−1.950236	4.936356
H	−0.487413	0.197674	5.943693
H	1.111688	1.013984	3.873852
H	0.568916	1.292193	−0.259719
H	−1.794155	2.859722	−5.342677

Table S2. DFT-calculated NMR chemical shifts of structures **1a** and **1b** for **1**.

^{13}C NMR data in DMSO-d_6, 125 MHz [ppm]

No.	Experimental	**1a**	**1b**	**|Exp. – 1a|**	**|Exp. – 1b|**
1	168.1	168.1	167.0	0.0	1.1
2	50.9	56.5	55.6	5.6	4.7
3	35.1	39.0	37.8	3.9	2.7
4	126.1	125.9	127.1	0.2	1.0
5	133.3	132.6	132.3	0.7	1.0
6	128.0	126.4	127.3	1.6	0.7
7	152.8	150.6	150.1	2.2	2.7
8	115.2	111.9	112.4	3.3	2.8
9	129.2	130.0	129.8	0.8	0.6
10	168.8	165.2	165.2	3.6	3.6
11	22.5	24.4	24.6	1.9	2.1
1’	171.1	167.2	169.1	3.9	2.0
2’	58.8	66.9	64.3	8.1	5.5
3’	29.2	34.0	33.1	4.8	3.9
4’	24.3	28.9	28.7	4.6	4.4
5’	46.7	49.9	49.2	3.2	2.5
1”	174.5	172.1	173.7	2.4	0.8
2”	53.0	55.9	56.2	2.9	3.2
3”	30.6	31.1	36.2	0.5	5.6
4”	114.0	109.8	114.4	4.2	0.4
5”	126.7	129.9	127.1	3.2	0.4
6”	119.8	122.6	121.1	2.8	1.3
7”	129.2	128.8	127.8	0.4	1.4
8”	123.3	118.2	118.7	5.1	4.6
9”	109.7	109.5	109.3	0.2	0.4
10”	135.3	133.3	133.4	2.0	1.9
11”	122.4	124.6	122.6	2.2	0.2
			MAE*	2.7	2.3

* MAE: Mean Absolute Error

^{1}H NMR data in DMSO-d_6, 500 MHz [ppm]

No.	Experimental	**1a**	**1b**	**|Exp. – 1a|**	**|Exp. – 1b|**
2	4.82	4.94	4.85	0.12	0.03
3*	2.80, 3.04	3.09, 3.16	3.09, 3.29	–	–
5	7.27	7.19	7.76	0.08	0.49
8	6.77	7.22	7.24	0.45	0.47
9	6.68	7.27	7.27	0.59	0.59
11	1.86	1.77	1.84	0.09	0.02
2’	4.62	3.86	4.50	0.76	0.12
3’ *	1.81, 2.06	2.01, 2.13	1.94, 2.30	–	–
4’ *	1.92, 2.02	2.16, 2.06	2.25, 2.03	–	–
5’ *	3.63, 3.69	3.73, 3.87	3.90, 3.71	–	–
2”	4.53	5.36	4.92	0.83	0.39
3” *	2.80, 3.33	3.44, 3.68	3.51, 3.16	–	–
6”	7.36	8.60	7.81	1.24	0.45
8”	7.62	7.73	7.84	0.11	0.22
9”	7.26	7.91	7.85	0.65	0.59
11”	7.07	7.65	7.61	0.58	0.54
2-NH*	7.15	6.22	6.22	0.93	0.93
2”-NH*	8.81	6.09	6.52	2.72	2.29
10”-NH*	10.70	8.36	8.24	2.34	2.46
			MAE**	0.50	0.36

* These values are not included in the calculation of MAE and DP4+.

** MAE: Mean Absolute Error

DP4+ analysis

	**1a**	**1b**
DP4+ (H data)	0.00%	100.00%
DP4+ (C data)	0.00%	100.00%
DP4+ (all data)	0.00%	100.00%
